# Sustainable Synthesis of Chiral Tetrahydrofurans through the Selective Dehydration of Pentoses

**DOI:** 10.1002/chem.201503510

**Published:** 2015-09-25

**Authors:** Robert W Foster, Christopher J Tame, Dejan-Krešimir Bučar, Helen C Hailes, Tom D Sheppard

**Affiliations:** [a] Department of Chemistry, University College London, Christopher Ingold Laboratories 20 Gordon Street, London, WC1H 0AJ (UK) E-mail: h.c.hailes@ucl.ac.uk tom.sheppard@chem.ucl.ac.uk Homepage: http://www.tomsheppard.eu; [b] GlaxoSmithKline, Medicines Research Centre Gunnels Wood Road, Stevenage, Herts, SG1 2NY (UK)

**Keywords:** arabinose, biomass, hydrazines, cyclization, tetrahydrofurans

## Abstract

l-Arabinose is an abundant resource available as a waste product of the sugar beet industry. Through use of a hydrazone-based strategy, l-arabinose was selectively dehydrated to form a chiral tetrahydrofuran on a multi-gram scale without the need for protecting groups. This approach was extended to other biomass-derived reducing sugars and the mechanism of the key cyclization investigated. This methodology was applied to the synthesis of a range of functionalized chiral tetrahydrofurans, as well as a formal synthesis of 3*R*-3-hydroxymuscarine.

The effective use of biomass, and in particular that generated as waste,[1] is essential to reduce the global dependence on petrochemical resources for the manufacture of valuable compounds, fuels and materials.[2] The majority of biomass is made up of carbohydrates, which are an abundant source of pentoses and hexoses.[3] For example, the refinement of sugar beet generates beet pulp as a major waste product, and this is a rich source of l-arabinose.[4] A variety of techniques has been developed to convert these biomass resources into valuable small molecules, such as the dehydration of pentoses under forcing acidic conditions to give furfural (Scheme 1), which can then be converted into various alcohols, alkenes, and heterocycles.[5] However, the majority of compounds prepared from pentoses and hexoses in this fashion are either achiral[6] or racemic mixtures where the stereochemistry of the chiral precursors is lost.[7] Using these products as intermediates in the synthesis of more complex targets may therefore require the reintroduction of stereocenters using asymmetric catalysis[8] or resolutions.[9]

**Scheme 1:**
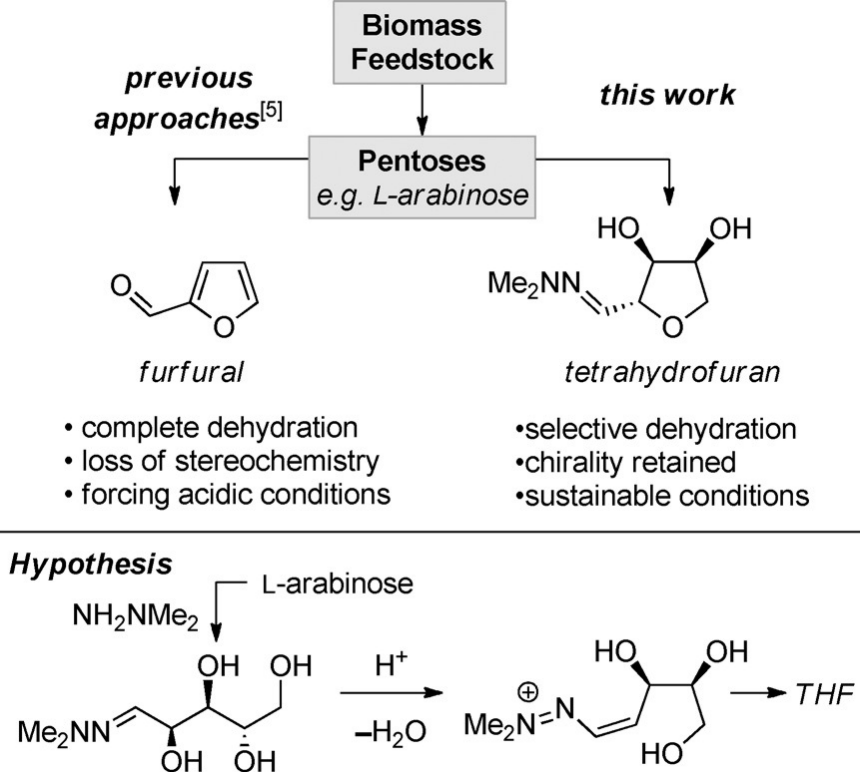
The preparation of furfural and THFs from biomass feedstock.

The tetrahydrofuran (THF) is a privileged scaffold within medicinal chemistry[10] and the stereoselective synthesis of chiral THFs has been a major area of recent research.[11] An attractive approach is to utilize the inherent chirality present in single isomer biomass-derived carbohydrates.[12] However, existing methods often require the selective conversion of a primary alcohol into an alkyl sulfonate or halide[13] and/or the use of protecting groups,[14] both of which are detrimental to the economy of a synthetic route.[15] Herein we describe the application of *N,N*-dimethylhydrazine[16] for the selective dehydration of biomass-derived reducing sugars to prepare chiral THFs under mildly acidic conditions (Scheme 1).[17]

Treating l-arabinose **1 a** with *N,N*-dimethylhydrazine and Amberlyst® 15 acidic resin in methanol at room temperature gave hydrazone **2 a** in 99 % yield (Table 1, entry 1). Stirring hydrazone **2 a** in methanol at 40 °C for 16 h with 20 mol % TFA resulted in 100 % conversion of **2 a**. Analysis of the crude ^1^H NMR spectrum indicated the formation of THF **3 a** as a 75:25 mixture of diastereoisomers and purification by flash column chromatography gave a mixture of the two stereoisomers in 67 % yield. The reaction was scaled up from a 6.7 mmol scale to a 104 mmol scale without any significant drop in yield, giving 11.9 g of THF **3 a**. The major diastereoisomer was isolated by recrystallization and the stereochemistry was confirmed by single-crystal X-ray diffraction (Figure 1). Both steps were conducted in a sustainable solvent[18] (methanol) without the need for either pre-drying of the solvent or for a drying agent in the reaction.

**Table 1:** Two-step synthesis of THFs 3 from sugars 1

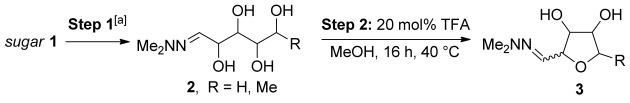					
Entry	Sugar1	Step 1yield [%]	THF3^[b]^	Step 2yield [%]	d.r.^[c]^
1	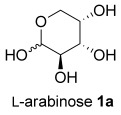	99	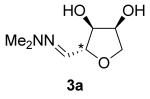	67 (66)^[d]^	75:25
2	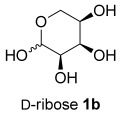	98	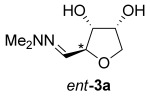	59	75:25
3	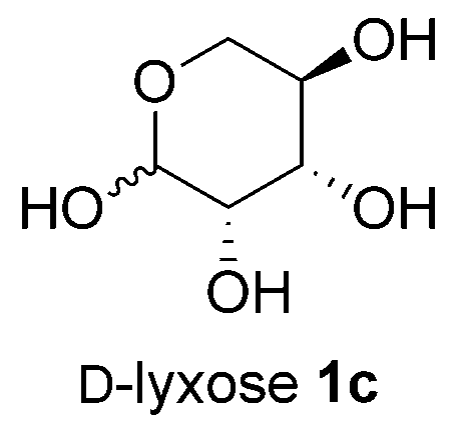	98	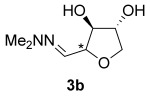	66	55:45
4	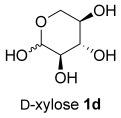	not isolated	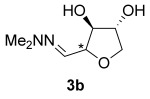	61^[e]^	55:45
5	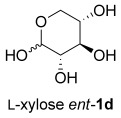	not isolated	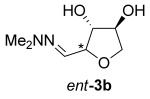	57^[e]^	55:45
6	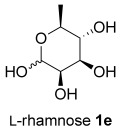	99	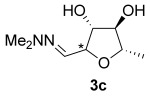	69	60:40

[a] Reagents and Conditions: NH_2_NMe_2_ (2.0 equiv), Amberlyst® 15, MeOH, 24 h, RT. [b] Reaction conducted on a 6.0–6.7 mmol scale unless otherwise stated. [c] Determined by analysis of the crude ^1^H NMR spectra. [d] Reaction conducted using 20.0 g (104 mmol) of hydrazone **2 a**. [e] Yield over two steps from xylose.

**Figure 1:**
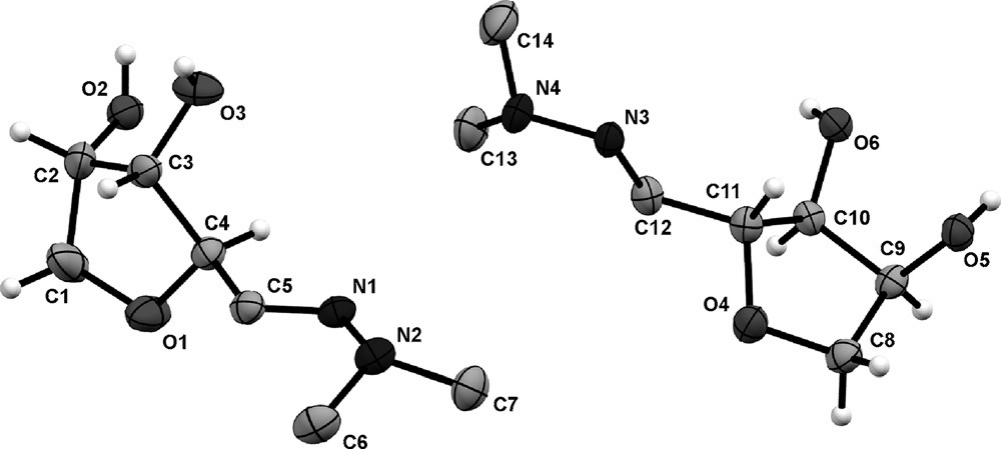
ORTEP of the asymmetric unit in the crystal structure of hydrazone *anti*-3 a. The thermal ellipsoids are shown at a 50 % probability level. Only hydrogen atoms belonging to the cyclic core are shown for clarity.[19]

The same reaction conditions were used to prepare the enantiomeric THF *ent*-**3 a** from d-ribose (Table 1, entry 2) in a 58 % yield over two steps. It is noteworthy that the diastereoselectivity of this reaction was comparable with that observed for the cyclization of arabinose-derived hydrazone **2 a**. The methodology was also extended to d-lyxose (Table 1, entry 3), with the corresponding hydrazone prepared in 98 % yield. The TFA-mediated cyclization step gave THF **3 b** in 66 % yield as a 55:45 mixture of diastereoisomers. THF **3 b** could also be prepared from d-xylose in 61 % yield over two steps, again as a 55:45 mixture of diastereoisomers (entry 4). This is a particularly important result as d-xylose is one of the major components of biomass.[3] Xylose is naturally available in both enantiomers and using l-xylose it was possible to access *ent*-**3 b** in a comparable yield (entry 5). The methodology was extended to deoxy sugar l-rhamnose, another constituent of sugar beet pulp, to give THF **3 c** in 69 % yield as a 60:40 mixture of diastereoisomers (entry 6).

Recrystallization of hydrazone **3 a** yielded the major *anti*-diastereoisomer in high purity. Reducing hydrazone *anti*-**3 a** using hydrogen, a palladium catalyst and Boc_2_O gave carbamate **4** in 60 % yield as a single stereoisomer (Scheme 2).

**Scheme 2:**
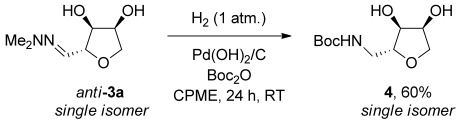
Reduction of hydrazone *anti*-3 a.

Treatment of THF **3 a** (d.r.=75:25) with Amberlyst® 15 acidic resin in water at room temperature resulted in rapid hydrolysis of the hydrazone to give hydrolyzed product **5** (Scheme 3).[20] Reduction of compound **5** with NaBH_4_ in methanol gave triol **6** as an 85:15 mixture of diastereoisomers in 98 % yield over two steps from hydrazone **3 a.** Reductive amination of intermediate **5** using *n*-butylamine, acetic acid, and hydrogen/palladium, followed by trapping of the intermediate amine with Boc_2_O, gave carbamate **7** in 65 % yield from hydrazone **3 a** as an 80:20 mixture of diastereoisomers. Compound **5** was also converted to alkene **8** using trimethyl phosphonoacetate in 73 % yield over two steps with excellent *E*-selectivity. Finally, treating compound **5** with Amberlyst® 15 in methanol resulted in the formation of dimethyl acetal **9** in 74 % yield over two steps from **3 a** as a 65:35 mixture of stereoisomers.

**Scheme 3:**
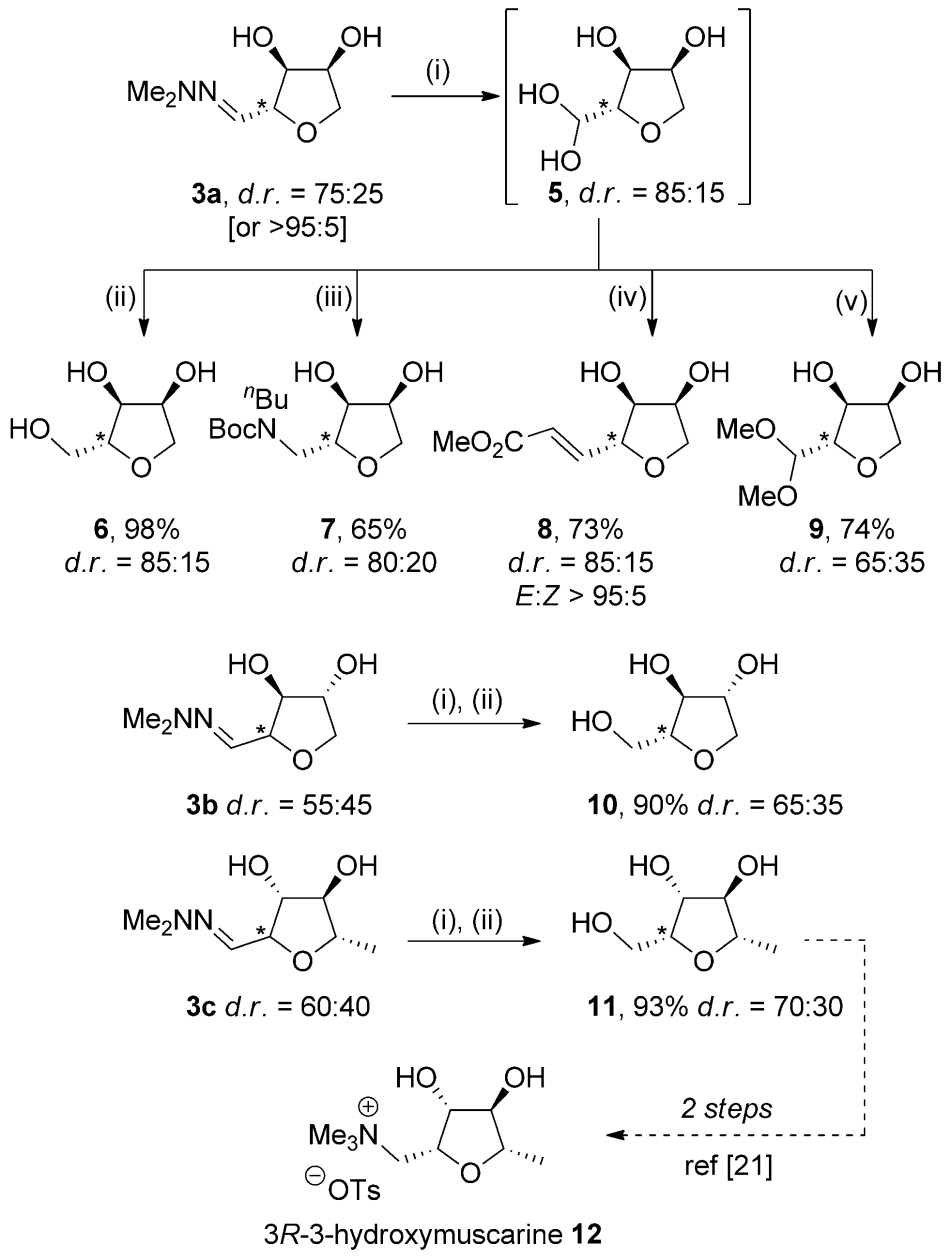
Hydrolysis of hydrazones 3 and transformation into a range of THFs. Reagents and conditions; i) Amberlyst® 15, H_2_O, 5 min, RT; ii) NaBH_4_, MeOH, 1 h, 0 °C; iii) *n*BuNH_2,_ AcOH, H_2_ (1 atm.), 10 % Pd/C, MeOH, 4 h, RT, then Boc_2_O, cyclopentyl methyl ether (CPME), 16 h, RT; iv) trimethyl phosphonoacetate, K_2_CO_3_, MeOH, 4 h 0 °C; v) Amberlyst® 15, MeOH, 48 h, RT.

The hydrolysis/reduction sequence was also applied to the hydrazones **3 b** and **3 c**, which gave the corresponding triols **10** and **11** in 90 % and 93 % yield respectively. l-Rhamnose-derived triol **11** is a late-stage intermediate in Fleet’s synthesis of 3*R*-3-hydroxymuscarine **12**.[21] Triol **11** was previously prepared from l-rhamnose using stoichiometric bromine, trifluoromethanesulfonic anhydride, and lithium aluminium hydride, so our route represents a less hazardous and more sustainable alternative.

A plausible reaction mechanism for the cyclization of hydrazone **2 a** is proposed in Scheme 4. The *N,N-*dialkylhydrazone group of **2 a** could promote the acid-mediated elimination of the adjacent hydroxyl to give vinyldiazenium intermediate **13**.[22] Cyclization of this intermediate would give THF **3 a** as either an *anti*- or *syn*-diastereoisomer. Resubmission of an isomerically pure sample of *anti*-**3 a** to the reaction conditions resulted in the same 75:25 mixture of *anti-* and *syn-*diastereoisomers that was observed in the original reaction, which suggests that the diastereoselectivity is under thermodynamic control. Conducting the reaction in [D_4_]MeOH did not result in detectable incorporation of deuterium adjacent to the hydrazone, indicating that epimerization occurs through a reversible ring closure rather than via a vinylhydrazine intermediate. The proposed mechanism is also consistent with the observation that hydrazones **2 a** and **2 b** converge to THF **3 a** and *ent***-3 a** with the same diastereoselectivity (Table 1, entries 1 and 2), as the two reactions would proceed through enantiomeric vinyldiazenium intermediates. Without TFA present no cyclization of **1 a** was observed.

**Scheme 4:**
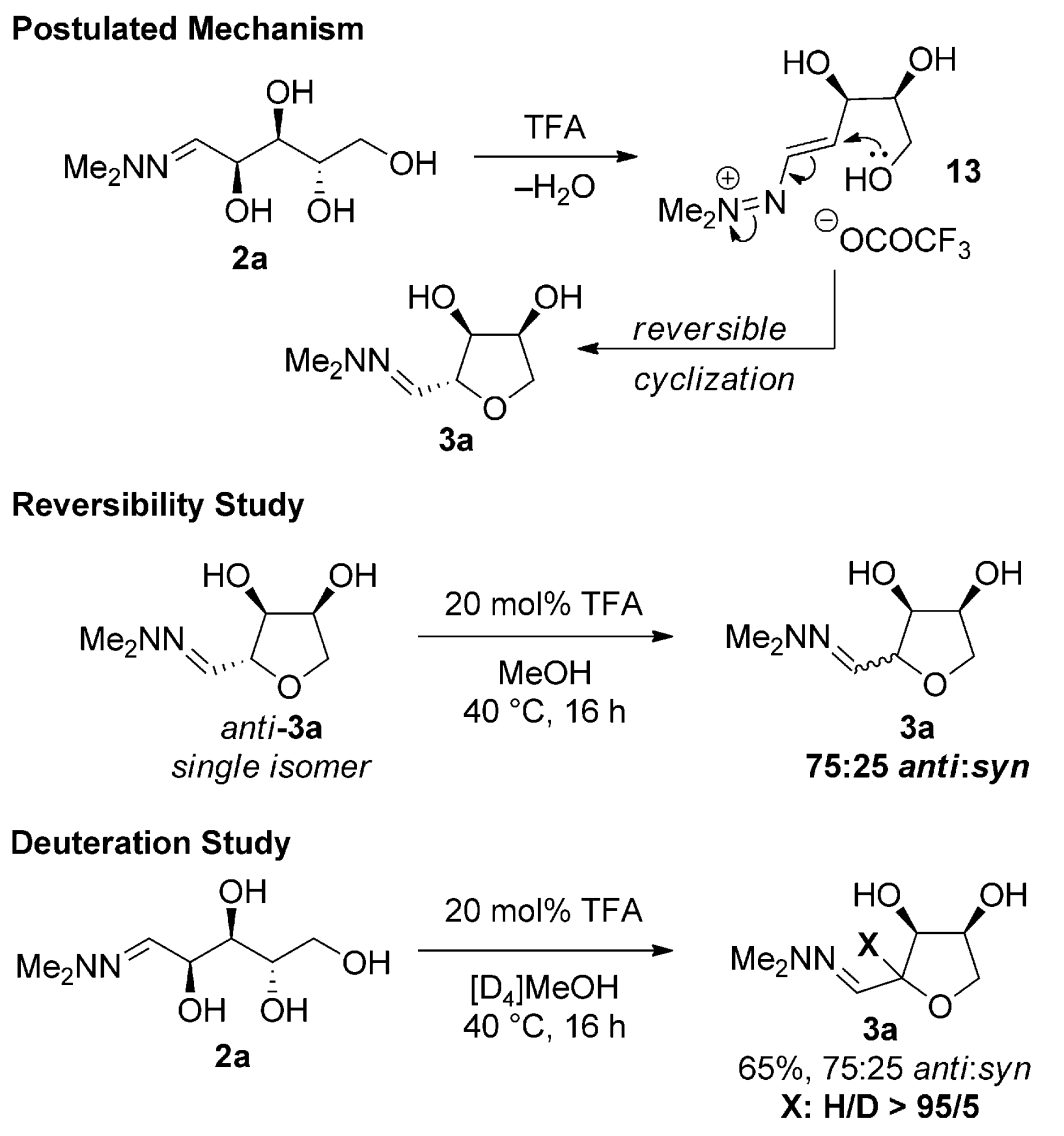
Postulated mechanism and mechanistic studies.

In a preliminary study, the extension of this approach to hexoses was explored (Scheme 5). Hydrazone **14**, formed from d-galactose, was subjected to the TFA-mediated cyclization conditions. This gave a 60:40 mixture of THF **15** and tetrahydropyran **16** in 53 % isolated yield, with both heterocycles formed as single stereoisomers.

**Scheme 5:**
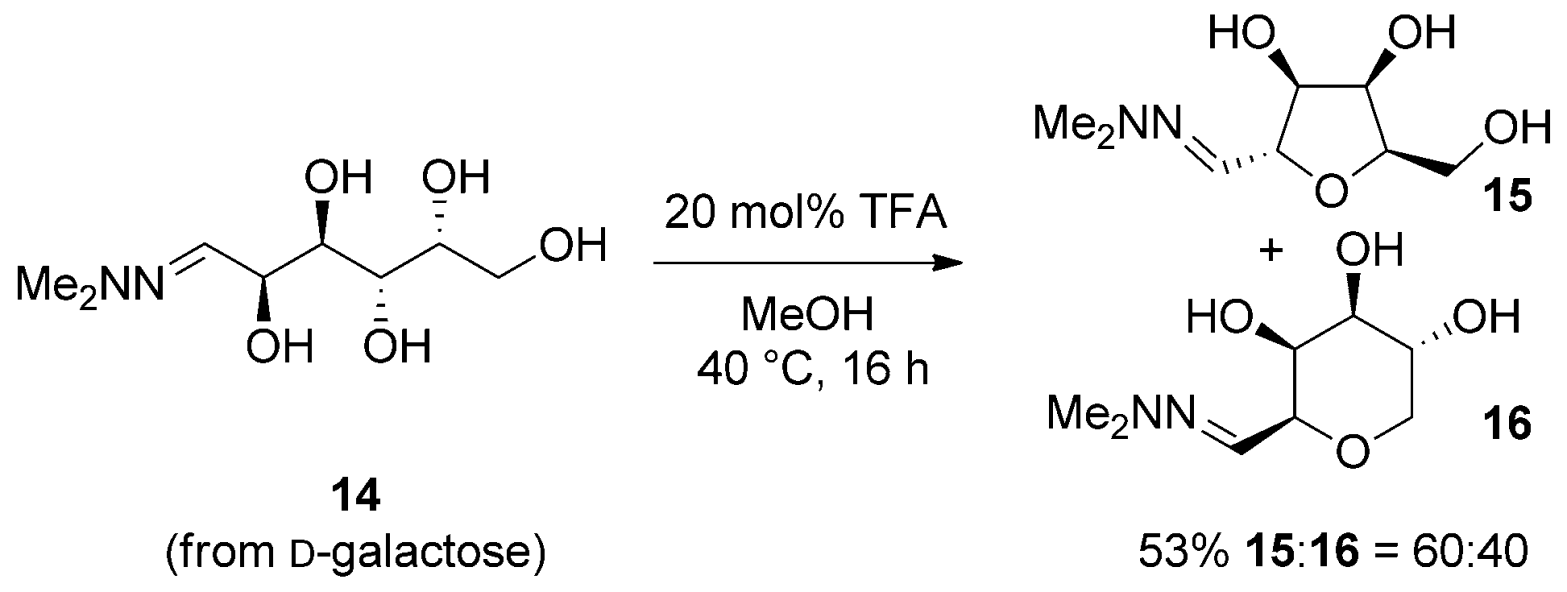
Extending the methodology to d-galactose.

In summary, we have developed an efficient multi-gram approach to low-molecular weight chiral molecules from biomass feedstock sources. This route allows access to a range of THF products without the need for protecting groups, including a formal synthesis of 3*R*-3-hydroxymuscarine. On the basis of experimental evidence, we have proposed a reaction mechanism for the key cyclization involving a vinyldiazenium intermediate.

## Experimental Section

Experimental procedures, ^1^H and ^13^C NMR spectra, characterization data for all compounds and crystallographic data for ***anti*****-3 a** are available in the Supporting Information.

A mixture of hydrazone **2 a** (20.0 g, 104 mmol) in MeOH (210 mL, 0.5 m) was treated with TFA (1.5 mL, 2.4 g, 20 mol %) at room temperature and the reaction stirred at 40 °C for 16 h. The reaction was quenched with aq. sat. NaHCO_3_ and concentrated in vacuo to give the crude THF (*anti*:*syn*=75:25). This was purified by flash column chromatography (80:100 hexane:acetone) to give THF **3 a** (11.9 g, 68.3 mmol, 66 %, *anti*:*syn*=75:25).

***anti*****-3 a**: Isolated as a single stereoisomer following recrystallization from boiling CPME; white crystalline solid; m.p.=65–67 °C; *R*_f_=0.33 (1:1 acetone:hexane); *ν*_max_ (film/cm^−1^) 3415 s br. 2875 s, 1586 s, 1467 s, 1445 s; ^1^H NMR (600 MHz; [D_4_]MeOH) 6.51 (1 H, d, *J*=6.6, N=C*H*), 4.23–4.18 (2 H, m, N=CHC*H*, CH_2_C*H*), 4.08 (1 H, dd, *J*=9.6, 4.9, OC*H*H′), 4.02 (1 H, dd, *J*=7.3, 5.1, N=CHCHC*H*), 3.76–3.72 (1 H, m, OCH*H*′), 2.79 (6 H, s, N(C*H*_3_)_2_); ^13^C NMR (150 MHz; [D_4_]MeOH) 135.6 (*C*=N), 82.5 (*C*HCH_2_), 76.5 (N=CHCH*C*H), 73.9 (O*C*H_2_), 72.4 (CH_2_CH*C*H), 42.8 (N(*C*H_3_)_2_); HRMS (EI^+^) found [M+H]^+^ 174.0979; C_7_H_14_N_2_O_3_ requires 174.0999; [α]_D_ (20 °C)=+85.8 (*anti*-**3 a**, MeOH, C=1.4).

## Supporting Information

As a service to our authors and readers, this journal provides supporting information supplied by the authors. Such materials are peer reviewed and may be re-organized for online delivery, but are not copy-edited or typeset. Technical support issues arising from supporting information (other than missing files) should be addressed to the authors.

miscellaneous_information
