# Supplementary material for: Sustainable Synthesis of Chiral Tetrahydrofurans through the Selective Dehydration of Pentoses
Source: Chemistry. 2015 Sep 25;21(45):15947–50. doi: 10.1002/chem.201503510 (PMC4648048; doi:10.1002/chem.201503510)
Supplement: Supplementary file 1 — miscellaneous_information [file chem0021-15947-sd1.pdf]

CHEMISTRY
A European Journal

Supporting Information

Sustainable Synthesis of Chiral Tetrahydrofurans through the Selective Dehydration of Pentoses

Robert W. Foster,[a] Christopher J. Tame,[b] Dejan-Krešimir Bučar,[a] Helen C. Hailes,*[a] and Tom D. Sheppard*[a]

chem_201503510_sm_miscellaneous_information.pdf

1. General Experimental Procedures

All solvents and chemicals were used as obtained from commercial suppliers. Column chromatography was carried out using BDH (40-63 µm) silica gel and analytical thin layer chromatography was carried out using Merck Kieselgel aluminium-backed plates coated with silica gel. Components were visualised using combinations of UV (254 nm) and potassium permanganate. Infrared (IR) spectra were recorded on a Perkin-Elmer spectrum 100 FT-IR spectrometer as thin films. ^{1}H and ^{13}C NMR spectra were recorded respectively at 400 MHz and 100 MHz on a Bruker Avance 400 spectrometer, 500 MHz and 125 MHz on a Bruker Avance 500 or at 600 MHz and 150 MHz on a Bruker Avance 600 spectrometer in the stated solvent. Mass spectra were obtained using either a VG70-SE or MAT 900XP spectrometer at the Department of Chemistry, University College London. All optical rotations were measured on a Perkin-Elmer 343 polarimeter with a path length of 1 dm.

2. General Experimental Procedures

General Hydrazone Synthesis Procedure

A stirring mixture of sugar **1** in methanol (2.0 M) was treated with NH_2NMe_2 (2 equivalents) and Amberlyst® 15 (1.00 g/100 mmol sugar) at room temperature. The resulting mixture was stirred at room temperature for 24 h before the mixture was filtered and the filtrate concentrated *in vacuo* to give the crude hydrazone.

General Acid-Catalyzed Cyclization Procedure

A stirring mixture of hydrazone **2** in methanol (0.5 M) was treated with TFA (20 mol%) at room temperature and the reaction stirred at 40 °C for 16 h. The reaction was then quenched with aq. sat. $NaHCO_3$ and concentrated *in vacuo* to give the crude hydrazone.

3. Compound Synthesis: Experimental Details & Compound Characterisation

(2*S*,3*R*,4*S*,*E*)-5-(2,2-Dimethylhydrazono)pentane-1,2,3,4-tetraol 2a[1]

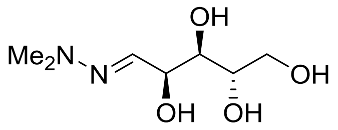

Prepared from L-arabinose **1a** (13.5 g, 90.0 mmol) according to the General Hydrazone Synthesis Procedure to give the hydrazone **2a** as a white crystalline solid (17.1 g, 89.5 mmol, 99%); m.p. = 93–95 °C (lit. m.p. = 88–90 °C)1; R_f = 0.24 (acetone); ν_{max} (film/cm^{-1}) 3420s (O-H), 3264s (O-H), 2938s (C-H), 1470s; ^{1}H NMR (600 MHz; DMSO-d_6) 6.61 (1H, d, *J* = 6.2, *H*C=N), 4.61 (1H, d, *J* = 5.8, CHO*H*), 4.53 (1H, d, *J* = 5.7, CHO*H*), 4.42 (1H, d, *J* = 7.2, CHO*H*), 4.33 (1H, t, *J* = 5.6, CH_2OH), 4.23–4.19 (1H, m, N=CHC*H*), 3.66–3.55 (1H, m, C*H*H'OH), 3.51–3.46 (1H, m, C*H*CH_2OH), 3.41–3.33 (1H, m, CH*H*'OH; *H*OD), 3.31–3.27 (1H, m, N=CHCHC*H*), 2.67 (6H, s, $N(CH_3)_2$); ^{13}C NMR (150 MHz; DMSO-d_6) 138.5 (*C*=N), 73.8 (*C*HCH_2OH), 71.2 (N=CHCH*C*H), 70.5 (N=CH*C*H), 63.5 (CH_2OH), 42.6 ($N(CH_3)_2$); $[\alpha]_D$ (20 °C) = –44.0 (MeOH, C = 1.0); data in accordance with the literature.[1]

(2*R*,3*S*,4*S*,*E*)-5-(2,2-Dimethylhydrazono)pentane-1,2,3,4-tetraol 2b

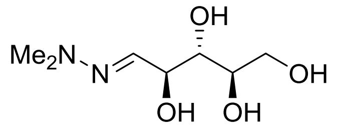

Prepared from D-ribose **1b** (1.00 g, 6.67 mmol) according to the General Hydrazone Synthesis Procedure to give the *hydrazone* **2b** as a white crystalline solid (1.26 g, 6.56 mmol, 98%); m.p. = 70–72 °C; R_f = 0.24 (acetone); ν_{max} (film/cm^{-1}) 3335s br. (O-H), 2992s (C-H), 1597s, 1469s, 1444s; ^{1}H NMR (600 MHz; DMSO-d_6) 6.56 (1H, d, *J* = 6.4, N=C*H*), 4.85 (1H, d, *J* = 5.1, CHO*H*), 4.63 (1H, d, *J* = 5.3, CHO*H*), 4.48 (1H, d, *J* = 4.9, CHO*H*), 4.31 (1H, t, *J* = 5.7, CH_2OH), 4.12–4.09 (1H, m, N=CHC*H*), 3.57–3.54 (1H, m, C*H*H'), 3.46–3.35 (3H, m, CH*H*'C*H*C*H*), 2.67 (6H, s, $N(CH_3)_2$); ^{13}C NMR (150 MHz; DMSO-d_6) 137.4 (N=*C*), 74.4 (*C*H), 72.3 (*C*H), 72.2 (*C*H), 63.2 (CH_2), 42.6 ($N(CH_3)_2$); HRMS (ESI$^+$) found $[M+H]^+$ 193.1180; $C_7H_{17}N_2O_4$ requires 193.1188; $[\alpha]_D$ (20 °C) = –22.6 (MeOH, C = 1.0).

(2*R*,3*R*,4*R*,*E*)-5-(2,2-Dimethylhydrazono)pentane-1,2,3,4-tetraol 2c

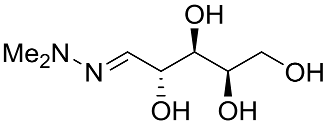

Prepared from D-lyxose **1c** (1.00 g, 6.67 mmol) according to the General Hydrazone Synthesis Procedure to give the *hydrazone* **2c** as a white crystalline solid (1.26 g, 6.56 mmol, 98%); m.p. = 73–75 °C; R_f = 0.14 (acetone); ν_{max} (film/cm^{-1}) 3336s br. (O-H), 2865s (C-H), 1599s, 1468s, 1443s; ^{1}H NMR (600 MHz; DMSO-d_6) 6.56 (1H, d, *J* = 6.2, N=C*H*), 4.84 (1H, d, *J* = 5.3, CHO*H*), 4.45 (1H, t, *J* = 5.6, CH_2O*H*), 4.22 (1H, d, *J* = 6.6, CHO*H*)), 4.19 (1H, d, *J* = 7.2, CHO*H*), 4.01–3.96 (1H, m, N=CHC*H*), 3.66–3.62 (1H, m, C*H*CH_2), 3.43–3.38 (2H, m, N=CHCHC*H*, C*H*H'OH), 3.37–3.33 (1H, m, CH*H*'OH; *H*OD), 2.68 (6H, s, N(CH_3)$_2$); ^{13}C NMR (150 MHz; DMSO-d_6) 138.5 (N=*C*), 72.8 (N=CHCH*C*H), 71.0 (N=CH*C*H), 70.3 (*C*HCH_2OH), 62.8 (*C*H_2OH), 42.6 (N(*C*H_3)$_2$); HRMS (ESI$^+$) found [M+H]$^+$ 193.1196; $C_7H_{17}N_2O_4$ requires 193.1188; $[\alpha]_D$ (20 °C) = +16.4 (MeOH, C = 0.58).

(2*S*,3*S*,4*S*,5*S*,*E*)-1-(2,2-Dimethylhydrazono)hexane-2,3,4,5-tetraol 2e

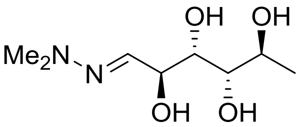

Prepared from L-rhamnose monohydrate **1e** (2.58 g, 14.2 mmol) according to the General Hydrazone Synthesis Procedure to give the *hydrazone* **2e** as a white crystalline solid (2.90 g, 14.1 mmol, 99%); m.p. = 101–103 °C; R_f = 0.30 (acetone); ν_{max} (film/cm^{-1}) 3347s br. (O-H), 2920w (C-H), 1611m, 1444s; ^{1}H NMR (600 MHz; DMSO-d_6) 6.57 (1H, d, *J* = 6.0, N=C*H*), 4.82 (1H, d, *J* = 5.3, CHO*H*), 4.41 (1H, d, *J* = 5.6, CHO*H*), 4.12 (1H, d, *J* = 7.7, CHO*H*), 4.08 (1H, d, *J* = 7.2, CHO*H*), 3.98–3.93 (1H, m, N=CHC*H*), 3.64–3.60 (1H, m, N=CHCHC*H*), 3.59–3.53 (1H, m, C*H*CH_3), 3.32–3.29 (1H, m, C*H*CHCH_3), 2.66 (6H, s, N(CH_3)$_2$), 1.10 (3H, d, *J* = 6.2, CHCH_3); ^{13}C NMR (150 MHz; DMSO-d_6) 138.8 (N=*C*), 73.5 (*C*H), 71.1 (*C*H), 71.0 (*C*H), 66.3 (*C*H), 42.6 (N(*C*H_3)$_2$), 20.8 (*C*H_3); HRMS (ESI$^+$) found [M+H]$^+$ 207.1347; $C_8H_{19}N_2O_4$ requires 207.1345; $[\alpha]_D$ (20 °C) = +4.1 (MeOH, C = 1.3).

(2*R*,3*S*,4*S*)-2-((*E*)-(2,2-Dimethylhydrazono)methyl)tetrahydrofuran-3,4-diol *anti*-3a and (2*S*,3*S*,4*S*)-2-((*E*)-(2,2-Dimethylhydrazono)methyl)tetrahydrofuran-3,4-diol *syn*-3a

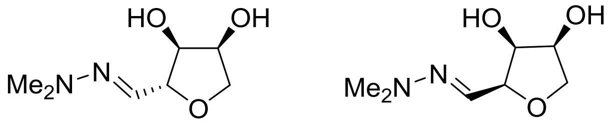

anti-**3a** *syn*-**3a**

Experiment A (6.60 mmol scale): Prepared from hydrazone **2a** (1.26 g, 6.60 mmol) according to the General Acid-Catalyzed Cyclization Procedure, to give the crude product (*anti*:*syn* = 75:25). This was purified by flash column chromatography (80:100 hexane:acetone) to give the *tetrahydrofuran* **3a** (772 mg, 4.44 mmol, 67%, *anti*:*syn* = 75:25).

Experiment B (104 mmol scale): Prepared from hydrazone **2a** (20.0 g, 104 mmol) according to the General Acid-Catalyzed Cyclization Procedure, to give the crude product (*anti*:*syn* = 75:25). This was purified by flash column chromatography (80:100 hexane:acetone) to give the *tetrahydrofuran* **3a** (11.9 g, 68.3 mmol, 66%, *anti*:*syn* = 75:25).

***Tetrahydrofuran anti*-3a:** Isolated as a single stereoisomer following recrystallization from boiling CPME; white crystalline solid; m.p. = 65–67 °C; R_f = 0.33 (1:1 acetone:hexane); ν_{max} (film/cm^{-1}) 3415s br. (O-H), 2875s (C-H), 1586s, 1467s, 1445s; ^1H NMR (600 MHz; MeOH-d_4) 6.51 (1H, d, *J* = 6.6, N=C*H*), 4.23–4.18 (2H, m, N=CHC*H*, CH_2C*H*), 4.08 (1H, dd, *J* = 9.6, 4.9, OC*H*H’), 4.02 (1H, dd, *J* = 7.3, 5.1, N=CHCHC*H*), 3.76–3.72 (1H, m, OCH*H*’), 2.79 (6H, s, N(CH_3)$_2$); ^{13}C NMR (150 MHz; MeOH-d_4) 135.6 (*C*=N), 82.5 (*C*HCH_2), 76.5 (N=CHCH*C*H), 73.9 (O*C*H_2), 72.4 (CH_2CH*C*H), 42.8 (N(*C*H_3)$_2$); HRMS (EI^+) found $[M]^+$ 174.0979; $C_7H_{14}N_2O_3$ requires 174.0999; $[\alpha]_D$ (20 °C) = +85.8 (*anti*-**3a**, MeOH, C = 1.4).

***Tetrahydrofuran syn*-3a**: ^1H NMR (600 MHz; MeOH-d_4) 6.71 (1H, d, *J* = 7.2, N=C*H*), 4.36–4.31 (2H, m, N=CHC*H*; CH_2C*H*), 4.15 (1H, t, *J* = 4.8, C*H*CHCH_2), 3.91 (1H, dd, *J* = 8.7, 6.2, OC*H*H’), 3.76–3.72 (1H, m, OCH*H*’), 2.79 (6H, s, N(CH_3)$_2$); ^{13}C NMR (150 MHz; MeOH-d_4) 135.6 (*C*=N), 83.1 (*C*HCH_2), 74.3 (N=CHCH*C*H), 73.2 (N=CH*C*H), 72.5 (O*C*H_2), 42.8 (N(*C*H_3)$_2$).

(2*S*,3*R*,4*R*)-2-((*E*)-(2,2-Dimethylhydrazono)methyl)tetrahydrofuran-3,4-diol *ent-syn*-3a and (2*R*,3*R*,4*R*)-2-((*E*)-(2,2-Dimethylhydrazono)methyl)tetrahydrofuran-3,4-diol *ent-syn*-3a

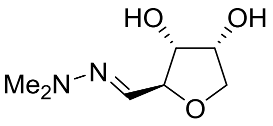

ent-anti-**3a**

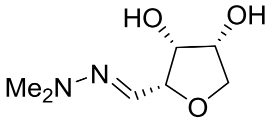

ent-syn-**3a**

Prepared from hydrazone **2b** (1.16 g, 6.03 mmol) according to General Acid-Catalyzed Cyclization Procedure to give the crude product (*anti*:*syn* = 75:25). This was purified by flash column chromatography (80:100 hexane:acetone) the *tetrahydrofuran ent*-**3a** as a yellow oil (620 mg, 3.56 mmol, 59%, *anti*:*syn* = 75:25); ^{1}H NMR consistent with **3a**; $[\alpha]_D$ (20 °C) = –24.2 (*ent*-**3a**, MeOH, C = 1.1).

(2*R*,3*R*,4*S*)-2-((*E*)-(2,2-Dimethylhydrazono)methyl)tetrahydrofuran-3,4-diol *anti*-3b and ((2*S*,3*R*,4*S*)-2-((*E*)-(2,2-Dimethylhydrazono)methyl)tetrahydrofuran-3,4-diol *syn*-3b

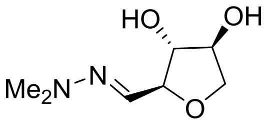

anti-**3b**

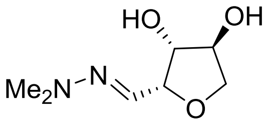

syn-**3b**

Method A: Prepared from hydrazone **2c** (1.22 g, 6.35 mmol) according to the General Acid-Catalyzed Cyclization Procedure to give the crude product (*d.r.* = 55:45). This was purified by flash column chromatography (80:100 hexane:acetone) to give the *tetrahydrofuran* **3b** (731 mg, 4.20 mmol, 66%, *d.r.* = 55:45).

Method B: Prepared according to the General Hydrazone Synthesis Procedure from D-xylose (1.00 g, 6.67 mmol) to give a crude hydrazone, which was subjected to the General Acid-Catalyzed Cyclization Procedure to give the crude product (*d.r.* = 55:45). This was purified by flash column chromatography (80:100 hexane:acetone) to give the *tetrahydrofuran* **3b** (711 mg, 4.09 mmol, 61% over 2 steps from D-xylose, *d.r.* = 55:45).

Isolated as a yellow oil; R_f = 0.20 (1:1 acetone:hexane); ν_{max} (film/cm^{-1}) 3360s br. (O-H), 2875s (C-H), 1595s, 1470s, 1445s; ^{1}H NMR (600 MHz; D_2O) 6.92 (1H, d, *J* = 6.6, N=C*H* major), 6.88 (1H, d, *J* = 6.4, N=C*H* minor), 4.60 (1H, dd, *J* = 6.4, 3.6, N=CHC*H* minor), 4.40–4.38 (1H, m, C*H*CH_2 minor), 4.35–4.33 (2H, m, C*H*CH_2, N=CHC*H* major), 4.26–4.23 (2H, m, C*H*CHC*H*H' minor), 4.20–4.19 (1H, m, C*H*CHCH_2 major), 4.10 (1H, dd, *J* = 10.0, 4.1, C*H*H' major), 3.98 (1H, dd, *J* = 10.0, 2.0, CH*H*' major), 3.83 (1H, dd, *J* = 10.0, 1.1, CH*H*' minor), 2.80 (6H, s, N(CH_3)$_2$ minor), 2.78 (6H,

s, N(CH_3)$_2$ major); ^{13}C NMR (150 MHz; D_2O, with a MeOH standard) 140.6 (*C*=N), 138.1 (*C*=N), 85.3 (*C*H), 81.0 (*C*H), 80.9 (*C*H), 78.1 (*C*H), 77.2 (*C*H), 77.1 (*C*H), 73.8 (*C*H_2), 73.7 (*C*H_2), 43.0 (N(*C*H_3)$_2$); HRMS (EI$^+$) found [M]$^+$ 174.0969; $C_7H_{14}N_2O_3$ requires 174.0999; $[\alpha]_D$ (20 °C) = +45.6 (MeOH, C = 1.1).

(2*R*,3*S*,4*R*)-2-((*E*)-(2,2-Dimethylhydrazono)methyl)tetrahydrofuran-3,4-diol *ent-anti*-3b and ((2*S*,3*S*,4*R*)-2-((*E*)-(2,2-Dimethylhydrazono)methyl)tetrahydrofuran-3,4-diol *ent-syn*-3b

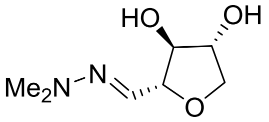

ent-anti-**3b**

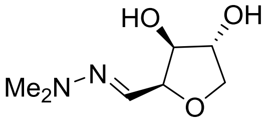

ent-syn-**3b**

Prepared according to the General Hydrazone Synthesis Procedure from L-xylose (1.00 g, 6.67 mmol) to give a crude hydrazone, which was subjected to the General Acid-Catalyzed Cyclization Procedure to give the crude product (*d.r.* = 55:45). This was purified by flash column chromatography (90:100 petroleum ether 40–60 °C:acetone) to give the *tetrahydrofuran ent*-**3b** as a yellow oil (656 mg, 3.77 mmol, 57% over 2 steps from L-xylose, *d.r.* = 55:45); ^{1}H NMR consistent with **3b**; $[\alpha]_D$ (20 °C) = –45.3 (MeOH C = 1.2).

(2*S*,3*R*,4*R*,5*S*)-2-((*E*)-(2,2-Dimethylhydrazono)methyl)-5-methyltetrahydrofuran-3,4-diol *anti*-3c and (2*R*,3*R*,4*R*,5*S*)-2-((*E*)-(2,2-Dimethylhydrazono)methyl)-5-methyltetrahydrofuran-3,4-diol *syn*-3c

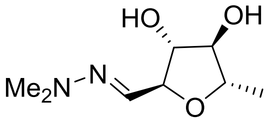

anti-**3c**

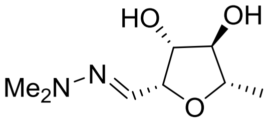

syn-**3c**

Prepared from hydrazone **2e** (1.37 g, 6.65 mmol) according General Acid-Catalyzed Cyclization Procedure to give the crude product (*d.r.* = 60:40). This was purified by flash column chromatography (80:100 hexane:acetone) to give the *tetrahydrofuran* **3c** as a yellow oil (866 mg, 4.61 mmol, 69%, *d.r.* = 60:40); R_f = 0.38 (1:1 acetone:hexane); ν_{max} (film/cm^{-1}) 3377s br. (O-H), 2921s (C-H), 1642s, 1445s; ^{1}H NMR (600 MHz; MeOH-d_4) 6.64 (1H, d, *J* = 7.0, N=C*H* minor), 6.61 (1H, d, *J* = 6.4, N=C*H* major), 4.41 (1H, dd, *J* = 7.0, 4.6, N=CHC*H* minor), 4.25 (1H, t, *J* = 6.4, N=CHC*H* major), 4.03 (1H, t, *J* = 6.4, N=CHCHC*H* major), 4.01–3.98 (1H, m, N=CHCHC*H* minor), 3.88 (1H, quintet, *J* = 6.4, C*H*CH_3 major), 3.79–3.74 (2H, m, C*H*C*H*CH_3 minor), 3.69 (1H, t, *J* = 6.4, C*H*CHCH_3 major), 2.81 (6H, s, N(CH_3)$_2$ minor), 2.79 (6H, s, N(CH_3)$_2$ major), 1.34 (3H, d,

J = 6.0, CHC*H*$_3$ minor), 1.28 (3H, d, *J* = 6.4, CHC*H*$_3$ major); ^{13}C NMR (150 MHz; MeOH-d$_4$) 136.1 (*C*=N major), 134.3 (*C*=N minor), 84.8 (*C*H minor), 84.1 (*C*H major), 83.7 (*C*H major), 82.7 (*C*H minor), 82.5 (*C*H minor), 81.8 (*C*H major), 80.9 (*C*H minor), 80.2 (*C*H major), 42.9 (N(*C*H$_3$)$_2$ major), 42.8 (N(*C*H$_3$)$_2$ minor), 19.6 (CH*C*H$_3$ minor), 19.3 (CH*C*H$_3$ major); HRMS (ESI$^+$) found [M+H]$^+$ 189.1235; $C_8H_{17}N_2O_3$ requires 189.1239; $[\alpha]_D$ (20 °C) = –33.2 (MeOH, C = 3.3).

tert-Butyl (((2*R*,3*S*,4*S*)-3,4-dihydroxytetrahydrofuran-2-yl)methyl)carbamate 4

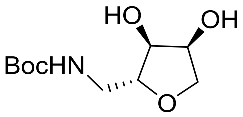

A stirring solution of tetrahydrofuran *anti*-**3a** (100 mg, 0.57 mmol) in CPME (2.9 mL) was treated with a solution of di-*tert*-butyl dicarbonate (313 mg, 1.44 mmol) in CPME (2.9 mL) at room temperature. The resulting solution was then treated with $Pd(OH)_2$ (20% on carbon, 163 mg) and the reaction was placed under an atmosphere of hydrogen (1 atm). The reaction was stirred at room temperature for 24 h before it was filtered through Celite and the filtrate concentrated *in vacuo* to give the crude product, which was purified by flash column chromatography (100:90 hexane:acetone) to give *carbamate* **4** as a colourless oil (80 mg, 0.34 mmol, 60%); R_f = 0.40 (100:90 hexane:acetone); ν_{max} (film/cm^{-1}) 3362s br. (O-H, N-H), 2977s (C-H), 1688s (C=O), 1523s, 1367s, 1251s; ^{1}H NMR (600 MHz; $CDCl_3$) 5.21–5.17 (1H, m, N*H*), 4.22–4.19 (1H, m, OCH$_2$C*H*), 4.05 (1H, dd, *J* = 10.2, 4.9, OC*H*H'), 3.84–3.80 (1H, m, NCH$_2$CHC*H*), 3.78–3.75 (2H, m, OCH*H*', NCH$_2$C*H*), 3.42–3.36 (1H, m, NC*H*H'), 3.31–3.26 (1H, m, NCH*H*'), 1.41 (9H, s, C(C*H*$_3$)$_3$); ^{13}C NMR (150 MHz; $CDCl_3$) 157.0 (*C*(O)), 80.6 (NCH$_2$*C*H), 80.2 (*C*Me$_3$), 73.2 (O*C*H$_2$), 73.1 (NCH$_2$CH*C*H), 71.2 (OCH$_2$*C*H), 42.0 (N*C*H$_2$), 28.5 (C(*C*H$_3$)$_3$) HRMS (ES$^+$) found [M+H]$^+$ 234.1339; $C_{10}H_{20}NO_5$ requires 234.1341; $[\alpha]_D$ (20 °C) = +32.9 (MeOH, C = 1.0).

(2*S*,3*S*,4*S*)-2-(Dihydroxymethyl)tetrahydrofuran-3,4-diol *anti*-5 and (2*R*,3*S*,4*S*)-2-(Dihydroxymethyl)tetrahydrofuran-3,4-diol *syn*-5

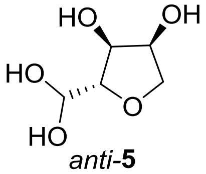

anti-**5**

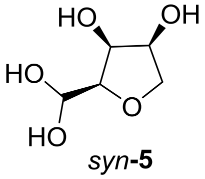

syn-**5**

Amberlyst 15 (6.90 g) was added to as stirring solution of hydrazone **3a** (1.20 g, 6.90 mmol) in water (34 mL) at room temperature. After 5 minutes the reaction was filtered and concentrated *in vacuo* and lyophilized to give the hydrolyzed product as a white gum (894 mg); R_f = 0.42 (acetone); ν_{max}

(film/cm^{-1}) 3345s br. (O-H), 2945s (C-H), 1720w, 1441m; HRMS (CI^+) found $[M–OH]^+$ 133.0496; $C_5H_9O_4$ requires 133.0495; $[\alpha]_D$ (20 °C) = +36.9 (MeOH, C = 1.0).

Analysis of the ^1H NMR in H_2O, D_2O, DMSO-d_6 and MeOH-d_4 suggested that the structure of the hydrolyzed product **5** was dependent on the solvent. In D_2O (accounting for deuterium exchange) the NMR data was consistent with a mixture of *hydrate anti*-**5** and *hydrate syn*-**5** (*anti*:*syn* = 85:15).

***Hydrate anti*-5 and *Hydrate syn*-5:** ^1H NMR (600 MHz; D_2O) 5.16 (1H, d, *J* = 7.2, C*H*$(OD)_2$ *syn*-**5**), 5.04 (1H, d, *J* = 5.1, C*H*$(OD)_2$ *anti*-**5**), 4.49 (1H, td, *J* = 7.2, 4.0, C*H*CH_2 *syn*-**5**), 4.31–4.27 (1H, m, C*H*CH_2 *anti*-**5**; 1H, m, C*H*CHCH_2 *syn*-**5**), 4.24 (1H, t, *J* = 5.1, C*H*CHCH_2 *anti*-**5**), 4.06–4.02 (1H, dd, *J* = 10.0, 4.1, C*H*H' *anti*-**5**; 1H, m, C*H*H' *syn*-**5**), 3.82 (1H, dd, *J* = 10.0, 3.0, CH*H*' *anti*-**5**), 3.79 (1H, dd, *J* = 7.2, 4.0, C*H*CH$(OD)_2$ *syn*-**5**), 3.75 (1H, t, *J* = 5.1, C*H*CH$(OD)_2$ *anti*-**5**), 3.71 (1H, t, *J* = 7.2, CH*H*' *syn*-**5**); ^{13}C NMR (150 MHz; D_2O with MeOH standard) 90.4 (*C*H$(OD)_2$ *anti*-**5**), 89.2 (*C*H$(OD)_2$ *syn*-**5**), 84.2 (*C*HCH$(OD)_2$ *anti*-**5**), 83.3 (*C*HCH$(OD)_2$ *syn*-**5**), 73.0 (C*H*CH_2 *anti*-**5**), 72.7 (O*C*H_2 *anti*-**5**), 71.9 (*C*HCHCH_2 *anti*-**5**), 71.8 (*C*HCH_2CH_2 *syn*-**5**), 71.5 (*C*HCH_2 *syn*-**5**), 70.8 (O*C*H_2 *syn*-**5**).

However analysis of the ^1H and ^{13}C NMR spectra in MeOH-d_4 revealed a more complex mixture of compounds, with the majority of material existing as a compound consistent with hemiacetal **B** (see below) [^{13}C.NMR (150 MHz; MeOH-d_4) 98.9 (*C*(OD)(OCD_3)), 98.7 (*C*(OD)(OCD_3))]. Analysis of ^1H and ^{13}C NMR spectra in DMSO-d_6 revealed an even more complex mixture of compounds, but with evidence for aldehyde **C** [^1H NMR (600 MHz; DMSO-d_6) 9.56 (1H, d, *J* = 2.3, C(O)*H*); ^{13}C NMR (150 MHz; DMSO-d_6) 201.9 (*C*(O)H)].

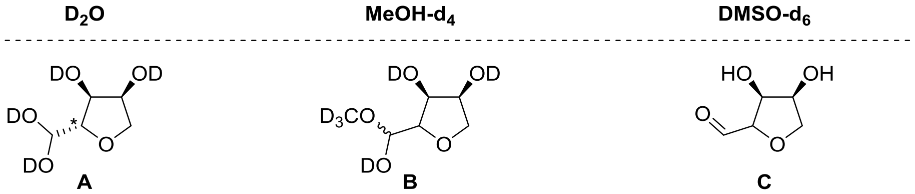

high purity
85:15 *anti*:*syn*

with other structures
stereochemistry undetermined

with other structures
stereochemistry undetermined

((2*S*,3*R*,4*S*)-2-((*E*)-(2,2-Dimethylhydrazono)methyl)tetrahydrofuran-3,4-diol *anti*-6 and (2*R*,3*R*,4*S*)-2-((*E*)-(2,2-Dimethylhydrazono)methyl)tetrahydrofuran-3,4-diol *syn*-6

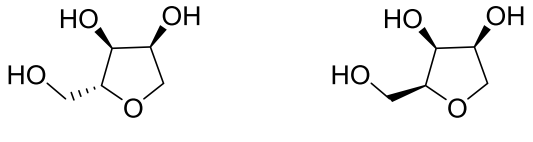

anti-**6** *syn*-**6**

A stirring solution of intermediate **5** (103 mg) in MeOH (3.9 mL) was treated with sodium borohydride (43 mg, 1.2 mmol) at 0 °C. The reaction was stirred at 0 °C for 1 h before the reaction was quenched with acetic acid (1 drop) and treated with Amberlyst 15 (1.6 g) and Amberlyst A26 (1.8 g). The mixture was then stirred at room temperature for 30 minutes before it was filtered the filtrate concentrated *in vacuo* to give the *triol* **6** as a white crystalline solid (104 mg, 0.776 mmol, 98% over two steps from hydrazone **3a**, *anti*:*syn* = 85:15); m.p. = 83–85 °C; R_f = 0.57 (acetone); ν_{max} (film/cm^{-1}) 3342s br. (O-H), 2930s (C-H), 1683w, 1411m; ^{1}H NMR (600 MHz; D_2O) 4.44 (1H, q, *J* = 5.8, C*H*CH_2 *syn*-**6**), 4.33 (1H, t, *J* = 4.8, C*H*CHCH_2 *syn*-**6**), 4.31–4.29 (1H, m, C*H*CH_2 *anti*-**6**), 4.13 (1H, dd, *J* = 7.3, 4.9, C*H*CHCH_2 *anti*-**6**), 4.09 (1H, dd, *J* = 10.2, 4.3, C*H*H' *anti*-**6**), 4.08–4.06 (1H, m, C*H*CH_2 *syn*-**6**), 3.99 (1H, dd, 9.2, 6.4, C*H*H' *syn*-**6**), 3.90–3.86 (1H, m, C*H*CH_2 *anti*-**6**), 3.85–3.81 (2H, m, C*H*H', CH*H*' *anti*-**6**; 1H, m, C*H*H' *syn*-**6**), 3.77–3.73 (2H, m, CH*H*', CH*H*' *syn*-**6**), 3.67 (1H, dd, *J* = 12.5, 5.1 CH*H*' *anti*-**6**); ^{13}C NMR (150 MHz; D_2O with MeOH reference) 82.1 (*C*H *anti*-**6**), 81.4 (*C*H *syn*-**6**), 72.8 ((*C*H_2 *anti*-**6**), 72.2 (*C*H *anti*-**6**), 71.8 (*C*H *syn*-**6**), 71.7 (*C*H *syn*-**6**), 71.7 (*C*H *anti*-**6**), 71.0 (*C*H_2 *syn*-**6**), 61.9 (*C*H_2 *anti*-**6**), 61.0 (*C*H_2 *syn*-**6**); HRMS (CI$^+$) found $[M+H]^+$ 135.0652; $C_5H_{11}O_4$ requires 135.0652; $[\alpha]_D$ (20 °C) = +50.9 (MeOH, C = 2.0); data in accordance with the literature.[2]

tert-Butyl butyl(((2*R*,3*S*,4*S*)-3,4-dihydroxytetrahydrofuran-2-yl)methyl)carbamate (*anti*-7) and _tert_-Butyl butyl(((2*S*,3*S*,4*S*)-3,4-dihydroxytetrahydrofuran-2-yl)methyl)carbamate (*syn*-7)

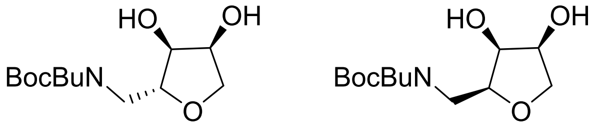

anti-**7** *syn*-**7**

A stirring solution of intermediate **5** (124 mg) in MeOH (4.7 mL) was treated with AcOH (27 µL, 28 mg, 0.47 mmol), ^{n}BuNH_2 (0.47 mL, 640 mg, 8.7 mmol) and 10% Pd/C (124 mg) at RT before the reaction was placed under an atmosphere of hydrogen. The reaction was stirred at RT for 4 h before it was filtered through Celite and the filtrate concentrated *in vacuo*. The crude product was then dissolved in CPME (2.0 mL) and stirred at RT. The reaction was treated with a solution of Boc_2O (308 mg, 1.41 mmol) in CPME (3.0 mL) and resulting mixture stirred at RT for 16 h. The reaction was then concentrated *in vacuo* to give the crude product, which was purified by flash column chromatography (70:30 hexane:acetone) to give the *carbamate* **7** as a white gum (179 mg, 0.619 mmol, 65% over two steps from hydrazone **3a**, *anti*:*syn* = 80:20); R_f = 0.38 (30:70 acetone:hexane); ν_{max} (film/cm^{-1}) 3406s br. (O-H), 2931s (C-H), 1690s, 1665s, 1479s, 1468s, 1416s; ^{1}H NMR (400 MHz; DMSO-d_6, 80 °C) 4.90–4.06 (2H, br. m, 2 × O*H* *anti*-**7**; 3H, br. m, 2 × O*H* *syn*-

7, C*H* *syn*-**7**), 4.05–4.00 (1H, m, C*H* *anti*-**7**), 3.94–3.88 (1H, m, *H* *syn*-**7**), 3.91 (1H, dd, *J* = 9.3, 5.3, C*H*H' *anti*-**7**), 3.88–3.83 (1H, m, C*H* *syn*-**7**), 3.76–3.70 (1H, m, C*H* *anti*-**7**, 1H, m, *H* *syn*-**7**), 3.66 (1H, dd, *J* = 6.0, 5.3, C*H* *anti*-**7**), 3.54–3.47 (2H, m, *H* *syn*-**7**), 3.51 (1H, dd, *J* = 9.3, 4.0, CH*H*' *anti*-**7**), 3.44 (1H, dd, *J* = 14.3, 4.0, C*H*H' *anti*-**7**), 3.27–3.15 (2H, m, $CH_2{}^n$Pr *anti*-**7**; 3H, m, $CH_2{}^n$Pr *syn*-**7**, *H* *syn*-**7**), 3.11 (1H, dd, *J* = 14.3, 7.3, CH*H*' *anti*-**7**), 1.52–1.44 (2H, m, $CH_2CH_2CH_3$ *anti*-**7**; 2H, m, $CH_2CH_2CH_3$ *syn*-**7**), 1.42 (9H, s, $C(CH_3)_3$ *anti*-**7**), 1.41 (9H, s, $C(CH_3)_3$ *syn*-**7**), 1.31–1.21 (2H, m, CH_2CH_3 *anti*-**7**; 2H, m, CH_2CH_3 *syn*-**7**), 0.90 (3H, t, *J* = 7.3, CH_2CH_3 *anti*-**7**; 3H, t, *J* = 7.3, CH_2CH_3 *syn*-**7**); ^{13}C NMR (150 MHz; DMSO-d_6) 154.9 (*C*(O)), 154.6 (*C*(O)), 81.2 (*C*H), 80.8 (*C*H), 78.3 (CO^tBu), 73.5 (*C*H), 72.2 (CH_2), 71.3 (*C*H), 70.9 (*C*H), 70.5 (CH_2), 70.1 (*C*H), 49.1 (CH_2), 48.8 (CH_2), 47.0 (CH_2), 46.5 (CH_2), 30.0 (CH_2), 29.5 (CH_2), 28.1 ($C(CH_3)_3$), 19.5 (CH_2), 13.8 (CH_2CH_3)); HRMS (ESI$^+$) found $[M+H]^+$ 290.1979; $C_{14}H_{28}NO_5$ requires 290.1967; $[\alpha]_D$ (20 °C) = +25.0 (MeOH, C = 1.0).

Methyl (*E*)-3-((2*R*,3*S*,4*S*)-3,4-dihydroxytetrahydrofuran-2-yl)acrylate *anti*-8 and Methyl (*E*)-3-((2*S*,3*S*,4*S*)-3,4-dihydroxytetrahydrofuran-2-yl)acrylate *syn*-8

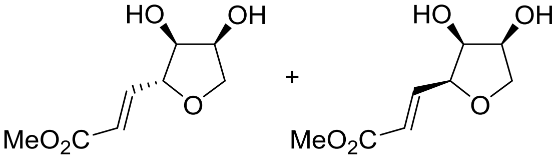

anti-**8** *syn*-**8**

Potassium carbonate (78 mg, 0.57 mmol) and trimethyl phosphonoacetate (74 µL, 82 mg, 0.43 mmol) were added to a stirring solution of intermediate **5** (50 mg, 0.38 mmol, 85:15 *anti*:*syn.*) in methanol (1.9 mL) at 0 °C. The reaction was stirred at 0 °C for 4 h before the reaction mixture was filtered through a silica plug (eluting with ethyl acetate) and the filtrate concentrated to give the crude product (*anti*:*syn*= 85:15). This was purified by flash column chromatography (CH_2Cl_2) to give the *alkene* **8** as a colourless oil (53 mg, 0.28 mmol, 73% over two steps from hydrazone **3a**, *anti*:*syn* = 85:15); R_f = 0.58 (1:1 acetone:hexane); ν_{max} (film/cm^{-1}) 3400s br. (O-H), 2944s (C-H), 1715s (C=O), 1649s, 1319s; ^{1}H NMR (600 MHz; MeOH-d_4) 7.02 (1H, dd, *J* = 15.6, 4.7, MeO_2CCH=C*H* *syn*-**8**), 6.98 (1H, dd, *J* = 15.8, 6.3, MeO_2CCH=C*H* *anti*-**8**), 6.08 (1H, dd, *J* = 15.8, 1.7, MeO_2CCH *anti*-**8**), 6.07–6.04 (1H, m, MeO_2CCH *syn*-**8**), 4.50 (1H, td, *J* = 4.7, 1.7, CH=CHC*H* *syn*-**8**), 4.34–4.31 (1H, m, C*H*CH_2 *syn*-**8**), 4.30–4.27 (1H, m, CH=CHC*H* *anti*-**8**), 4.19 (1H, t, *J* = 4.7, C*H*CHCH_2 *syn*-**8**), 4.18–4.15 (1H, m, CH_2C*H* *anti*-**8**), 4.12 (1H, dd, *J* = 9.8, 4.5, C*H*H' *anti*-**8**), 3.96–3.93 (1H, m, C*H*H' *syn*-**8**), 3.88–3.78 (2H, m, CH*H*'CHC*H* *anti*-**8**), 3.76–3.74 (1H, m, CH*H*' *syn*-**8**), 3.73 (3H, s, OCH_3 *anti*-**8**), 3.72 (3H, s, OCH_3 *syn*-**8**); ^{13}C NMR (150 MHz; MeOH-d_4) 168.3

(C(O), *anti*-**8**; C(O), *syn*-**8**), 148.1 ($MeO_2CCH{=}CH$ *anti*-**8**), 146.7 ($MeO_2CCH{=}CH$ *syn*-**8**), 122.5 ($MeO_2CCH{=}CH$ *syn*-**8**), 121.5 ($MeO_2CCH{=}CH$ *anti*-**8**), 81.6 (CH=CHCH *syn*-**8**), 81.5 (CH=CHCH *anti*-**8**), 77.7 (CH_2CHCH *anti*-**8**), 74.3 (CH_2 *anti*-**8**), 74.2 (*C*HOD *syn*-**8**), 73.1 (*C*HOD *syn*-**8**), 72.6 (CH_2 *syn*-**8**), 72.4 (CH_2CH *anti*-**8**), 52.1 (OCH_3 *anti*-**8**), 52.1 (OCH_3 *syn*-**8**); HRMS (EI^+) found $[M]^+$ 188.0680; $C_8H_{12}O_5$ requires 188.0679; $[\alpha]_D$ (20 °C) = +40.5 (MeOH, C = 0.45).

(2*S*,3*S*,4*S*)-2-(Dimethoxymethyl)tetrahydrofuran-3,4-diol *anti*-9 and (2*R*,3*S*,4*S*)-2-(Dimethoxymethyl)tetrahydrofuran-3,4-diol *syn*-9

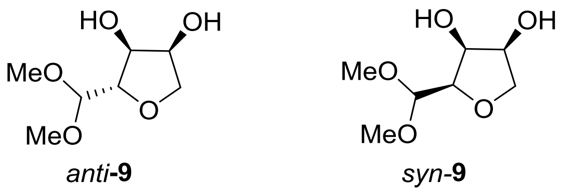

Amberlyst 15 (83 mg) was added to a stirring solution of intermediate **5** (109 mg) in methanol (4.1 mL) room temperature. The reaction was stirred at room temperature for 48 h before the reaction mixture was filtered and the filtrate concentrated to give the crude product (*anti*:*syn* = 65:35). This was purified by flash column chromatography (80:100 hexane:acetone) to give the acetal-**9** as a colourless oil (111 mg, 0.624 mmol, 74% over two steps from hydrazone **3a**, *anti*:*syn* = 75:25); R_f = 0.34 (1:1 hexane:acetone); ν_{max} (film/cm^{-1}) 3443s br. (O-H), 2947s (C-H); ^1H NMR (600 MHz; MeOH-d_4) 4.59 (1H, d, J = 7.5, C*H*$(OMe)_2$ *syn*-**9**), 4.33–4.29 (1H, m, C*H*CH_2 *syn*-**9**), 4.31 (1H, d, J = 4.7, C*H*$(OMe)_2$ *anti*-**9**), 4.13 (1H, q, J = 4.7, C*H*CH_2 *anti*-**9**), 4.10–4.06 (1H, m, C*H*CHCH$(OMe)_2$ *syn*-**9**), 4.09 (1H, t, J = 4.7, C*H*CHCH$(OMe)_2$ *anti*-**9**), 3.93 (1H, dd, J = 9.2, 4.7, C*H*H' *anti*-**9**), 3.89 (1H, t, J = 7.9, C*H*H' *syn*-**9**), 3.83 (1H, t, J = 4.7, C*H*CH$(OMe)_2$ *anti*-**9**), 3.79 (1H, dd, J = 7.5, 3.4, C*H*CH$(OMe)_2$ *syn*-**9**), 3.68 (1H, dd, J = 9.0, 4.3, CH*H*' *anti*-**9**), 3.65 (1H, t, J = 7.9, CH*H*' *syn*-**9**), 3.44 (3H, s, OCH_3 *syn*-**9**), 3.43 (3H, s, OCH_3 *anti*-**9**), 3.42 (3H, s, OCH_3 *anti*-**9**), 3.39 (3H, s, OCH_3 *syn*-**9**); ^{13}C NMR (150 MHz; MeOH-d_4) 106.3 ($C(OMe)_2$ *anti*-**9**), 104.2 ($C(OMe)_2$ *syn*-**9**), 84.1 (*C*HCH$(OMe)_2$ *anti*-**9**), 81.5 (*C*HCH$(OMe)_2$ *syn*-**9**), 73.6 (*C*HCHCH_2 *anti*-**9**), 73.3 (CH_2O *anti*-**9**), 73.0 (*C*HO *syn*-**9**), 72.6 (*C*HCH_2 *anti*-**9**), 72.5 (*C*HO *syn*-**9**), 72.1 (CH_2O *syn*-**9**), 56.1 ($O(CH_3)_2$ *anti*-**9**), 55.2 ($O(CH_3)_2$ *syn*-**9**); HRMS (ESI^+) found $[M+Na]^+$ 201.0741; $C_7H_{14}O_5Na$ requires 201.0739; $[\alpha]_D$ (20 °C) = +30.1 (MeOH, C = 0.38).

(2*R*,3*S*,4*R*)-2-(Hydroxymethyl)tetrahydrofuran-3,4-diol (*anti*-10)Error! Bookmark not defined. **and (2*S*,3*S*,4*R*)-2-(Hydroxymethyl)tetrahydrofuran-3,4-diol (*syn*-10)**

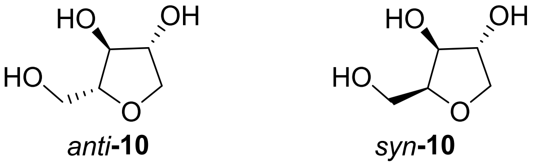

Amberlyst 15 (701 mg) was added to a stirring solution of hydrazone **3b** (122 mg, 0.701 mmol, 55:45 *d.r.*) in water (3.5 mL) at RT. After 5 minutes the reaction mixture was filtered and the filtrate concentrated *in vacuo* and lyophilized to give a white gum. The intermediate was then dissolved in MeOH (3.5 mL) and treated with $NaBH_4$ (40 mg, 1.1 mmol) portionwise at 0 °C and the reaction stirred at 0 °C for 1 h. The reaction was then quenched with AcOH (1 drop) and treated with Amberlyst 15 (1.4 0g) and Amberlyst A26 (1.40 g). The mixture was then stirred at RT for 30 minutes before it was filtered the filtrate concentrated *in vacuo* to give *triol* **10** as a colorless oil (85 mg, 0.63 mmol, 90%, *anti*:*syn* = 65:35); R_f = 0.57 (acetone); ν_{max} (film/cm^{-1}) 3330s br. (O-H), 2939s (C-H), 1655m, 1414s; ^{1}H NMR (600 MHz; D_2O) 4.33–4.29 (1H, m, C*H* *syn*-**10**), 4.27–4.25 (1H, m, C*H* *anti*-**10**), 4.23 (1H, dd, *J* = 3.6, 1.3, C*H* *syn*-**10**), 4.18 (1H, dd, *J* = 10.3, 4.2, C*H*H' *syn*-**10**), 4.16–4.12 (1H, m, C*H* *syn*-**10**), 4.05–4.02 (2H, m, C*H*, C*H*H' *anti*-**10**), 3.89–3.84 (2H, m, C*H*, CH*H'* *anti*-**10**; 1H, m, C*H*H' *syn*-**10**), 3.80–3.71 (2H, m, CH_2 *anti*-**10**; 2H, m, CH*H*', CH*H*' *syn*-**10**); ^{13}C NMR (150 MHz; D_2O with MeOH standard) 86.1 (*C*H *anti*-**10**), 81.4 (*C*H *syn*-**10**), 78.5 (*C*H *anti*-**10**), 77.5 (*C*H *anti*-**10**), 77.2 (*C*H *syn*-**10**), 76.7 (*C*H *syn*-**10**), 73.4 (*C*H_2 *anti*-**10**), 73.3 (*C*H_2 *syn*-**10**), 62.2 (*C*H_2 *anti*-**10**), 60.5 (*C*H_2 *syn*-**10**); HRMS (ESI$^+$) found [M+H]$^+$ 135.0658; $C_5H_{11}O_4$ requires 135.0652; $[\alpha]_D$ (20 °C) = +69.6 (MeOH, C = 0.17); Data for *anti*-**10** in accordance with the literature.**Error! Bookmark not defined.**

(2*R*,3*R*,4*R*,5*S*)-2-(Hydroxymethyl)-5-methyltetrahydrofuran-3,4-diol *syn*-11[3] and (2*S*,3*R*,4*R*,5*S*)-2-(Hydroxymethyl)-5-methyltetrahydrofuran-3,4-diol *anti*-11

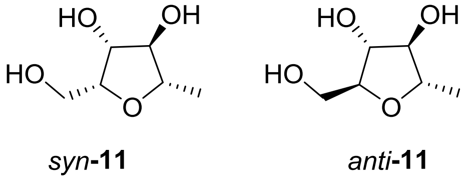

Amberlyst 15 (888 mg) was added to a stirring solution of hydrazone **3c** (167 mg, 0.888 mmol, 60:40 *d.r.*) in water (4.4 mL) at room temperature. After 5 minutes the reaction mixture was filtered and the filtrate concentrated *in vacuo* and lyophilized to give a white gum. The intermediate was then dissolved in methanol (4.4 mL) and treated with $NaBH_4$ (49 mg, 1.3 mmol) at 0 °C and the reaction

stirred at 0 °C for 1 h. The reaction was then quenched with acetic acid (1 drop) and treated with Amberlyst 15 (1.80 g) and Amberlyst A26 (1.80 g). The mixture was then stirred at room temperature for 30 minutes before it was filtered and the filtrate concentrated *in vacuo* to give the *triol* **11** as a colourless oil (122 mg, 0.824 mmol, 93%, *syn*:*anti* = 70:30); R_f = 0.26 (1:1 acetone:hexane); ν_{max} (film/cm^{-1}) 3317s (O-H), 2930s (C-H), 1450s; ^{1}H NMR (600 MHz; D_2O) 4.21–4.19 (1H, m, C*H* *anti*-**11**), 4.09–4.06 (1H, m, C*H* *anti*-**11**), 4.04 (1H, t, *J* = 6.4, C*H*OD *syn*-**11**), 3.96–3.91 (2H, m, C*H*CH_2, C*H*CH_3 *syn*-**11**), 3.86–3.80 (1H, m, C*H*OD *syn*-**11**; 3H, m, C*H*CH_3, C*H*H’, C*H* *anti*-**11**), 3.78–3.73 (1H, m, C*H*H’ *syn*-**11**; 1H, m, C*H*H’OD *anti*-**11**), 3.72–3.69 (1H, dd, *J* = 12.4, 5.8, CH*H*’OD *syn*-**11**), 1.37–1.35 (3H, m, CHCH_3 *anti*-**11**), 1.32 (3H, d, *J* = 6.4, CHCH_3 *syn*-**11**); ^{13}C NMR (150 MHz; D_2O with MeOH standard) 83.4 (*C*H *anti*-**11**), 82.5 (*C*H *syn*-**11**), 82.3 (*C*H *syn*-**11**), 81.3 (*C*H *anti*-**11**), 80.9 (*C*H *anti*-**11**), 78.6 (*C*H *syn*-**11**), 78.2 (*C*H *anti*-**11**), 77.2 (*C*H *syn*-**11**), 61.9 (*C*H_2 *syn*-**11**), 60.7 (*C*H_2 *anti*-**11**), 18.7 (*C*H_3 *anti*-**11**), 18.3 (*C*H_3 *syn*-**11**); HRMS (CI^+) found $[M+H]^+$ 149.0821; $C_6H_{13}O_4$ requires 149.0808; $[\alpha]_D$ (20 °C) = –28.7 (MeOH, C = 3.3); Data for *syn*-**11** accordance with the literature.[3]

(2*R*,3*S*,4*R*,5*S*,*E*)-6-(2,2-Dimethylhydrazono)hexane-1,2,3,4,5-pentaol 14[1]

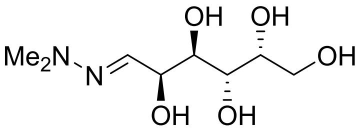

A stirring solution of D-galactose (1.80 g, 10.0 mmol) in methanol (20 mL, 0.5 M) was treated with NH_2NMe_2 (1.5 mL, 1.2 g, 20 mmol) and Amberlyst 15 (2.00 g) at room temperature and the reaction stirred at room temperature for 3 days. The reaction was then filtered and the filtrate concentrated *in vacuo* to give the crude product, which was purified by flash column chromatography (acetone) to give the *hydrazone* **14** as a yellow crystalline solid (680 mg, 3.06 mmol, 31%); m.p. = 106–108 °C (lit. m.p. = 96–100 °C) [1]; R_f = 0.20 (1:5 MeOH:acetone); ν_{max} (film/cm^{-1}) 3362s br. (O-H), 2931s (C-H), 1593s, 1469s, 1412s; ^{1}H NMR (600 MHz; DMSO-d_6) 6.65 (1H, d, *J* = 6.0, N=C*H*), 4.53 (1H, d, *J* = 6.4, CHO*H*), 4.43 (1H, t, *J* = 5.6, CH_2O*H*), 4.33 (1H, d, *J* = 7.5, CHO*H*), 4.27–4.24 (1H, m, N=CHC*H*), 4.14–4.10 (2H, m, 2 × CHO*H*), 3.70 (1H, dd, *J* = 6.5, 1.4, C*H*OH), 3.51–3.47 (1H, m, C*H*OH), 3.44–3.36 (3H, m, C*H*OH, CH_2OH), 2.67 (6H, s, N(CH_3)$_2$); ^{13}C NMR (150 MHz; DMSO-d_6) 139.0 (N=*C*), 72.5 (*C*H), 70.4 (*C*H), 69.9 (*C*H), 69.1 (*C*H), 63.1 (*C*H_2), 42.7 (N(*C*H_3)$_2$); $[\alpha]_D$ (20 °C) = –30.0 (MeOH, C = 1.0); Data in accordance with the literature.[1]

(2*R*,3*S*,4*R*,5*R*)-2-((*E*)-(2,2-Dimethylhydrazono)methyl)-5-(hydroxymethyl)tetrahydrofuran-3,4-diol 15 and (2*S*,3*S*,4*S*,5*R*)-2-((*E*)-(2,2-Dimethylhydrazono)methyl)tetrahydro-2*H*-pyran-3,4,5-triol 16

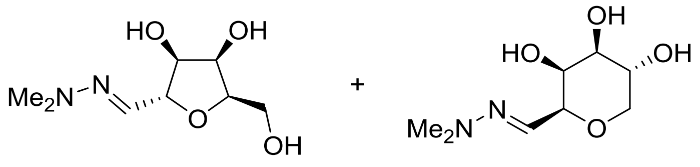

Prepared from hydrazone **14** (222 mg, 1.00 mmol) according to the General Acid-Catalyzed Cyclization Procedure to give the crude product (**15**:**16** = 60:40). This was purified by flash column chromatography (30:70 hexane:acetone) to give the *tetrahydrofuran* **15** as a colourless oil (59 mg, 0.29 mmol, 29%). Further elution of the column gave the *tetrahydropyran* **16** as a colourless oil (49 mg, 0.24 mmol, 24%, 20% impurity of **15**). Further purification of the second fraction by flash column chromatography (30:70 hexane:acetone) gave an analytical sample of the *tetrahydropyran* **16** (7 mg).

***Tetrahydrofuran* 15:** R_f = 0.43 (70:30 acetone:hexane); ν_{max} (film/cm^{-1}) 3372s br. (O-H), 2898s (C-H), 1599w, 1444m, 1406m; ^{1}H NMR (600 MHz; D_2O) 6.93 (1H, d, *J* = 6.0, N=C*H*), 4.22 (1H, dd, *J* = 9.4, 6.0, N=CHC*H*), 4.15–4.12 (1H, m, C*H*CHCH$_2$), 4.04 (1H, dd, *J* = 9.4, 3.2, N=CHCHC*H*), 4.01–3.98 (2H, m, C*H*C*H*H'), 3.85–3.82 (1H, m, CH*H*'), 2.89 (6H, s, N(C*H*$_3$)$_2$); ^{13}C NMR (150 MHz; D_2O with MeOH standard) 139.0 (*C*=N), 75.8 (*C*H), 70.0 (*C*H), 69.8 (*C*H), 67.6 (*C*H), 66.7 (*C*H$_2$), 43.0 (N(*C*H$_3$)$_2$); HRMS (ES$^+$) found [M+H]$^+$ 205.1181; $C_8H_{17}N_2O_4$ requires 205.1188; $[\alpha]_D$ (20 °C) = +1.2 (MeOH, C = 0.5). NOESY experiment shows strong NOE between 6.93 (1H, d, *J* = 6.0, N=C*H*) and 4.04 (1H, dd, *J* = 9.4, 3.2, N=CHCHC*H*).

***Tetrahydropyran* 16:** R_f = 0.28 (1:1 acetone:hexane); ν_{max} (film/cm^{-1}) 3369s br. (O-H), 2858s (C-H), 1596m, 1443m; ^{1}H NMR (600 MHz; MeOH-d$_4$) 6.67 (1H, d, *J* = 5.7, N=C*H*), 3.94–3.91 (2H, m, C*H*H', N=CHC*H*), 3.87 (1H, d, *J* = 3.4, N=CHCHC*H*), 3.84–3.79 (1H, m, C*H*CH$_2$), 3.44 (1H, dd, *J* = 9.4, 3.4, C*H*CHCH$_2$), 3.14 (1H, t, *J* = 10.9, CH*H*'), 2.79 (6H, s, N(C*H*$_3$)$_2$); ^{13}C NMR (150 MHz; MeOH-d$_4$) 135.1 (*C*=N), 80.8 (*C*H), 76.2 (*C*H), 73.1 (*C*H), 71.1 (*C*H$_2$), 68.1 (*C*H), 42.8 (N(*C*H$_3$)$_2$); HRMS (EI$^+$) found [M]$^+$ 204.1104; $C_8H_{16}N_2O_4$ requires 204.1105; $[\alpha]_D$ (20 °C) = –2.8 (MeOH, C = 0.5).

4. NMR Spectra

(2*S*,3*R*,4*S*,*E*)-5-(2,2-Dimethylhydrazono)pentane-1,2,3,4-tetraol 1a

^{1}H NMR (600 MHz, DMSO-d_6)

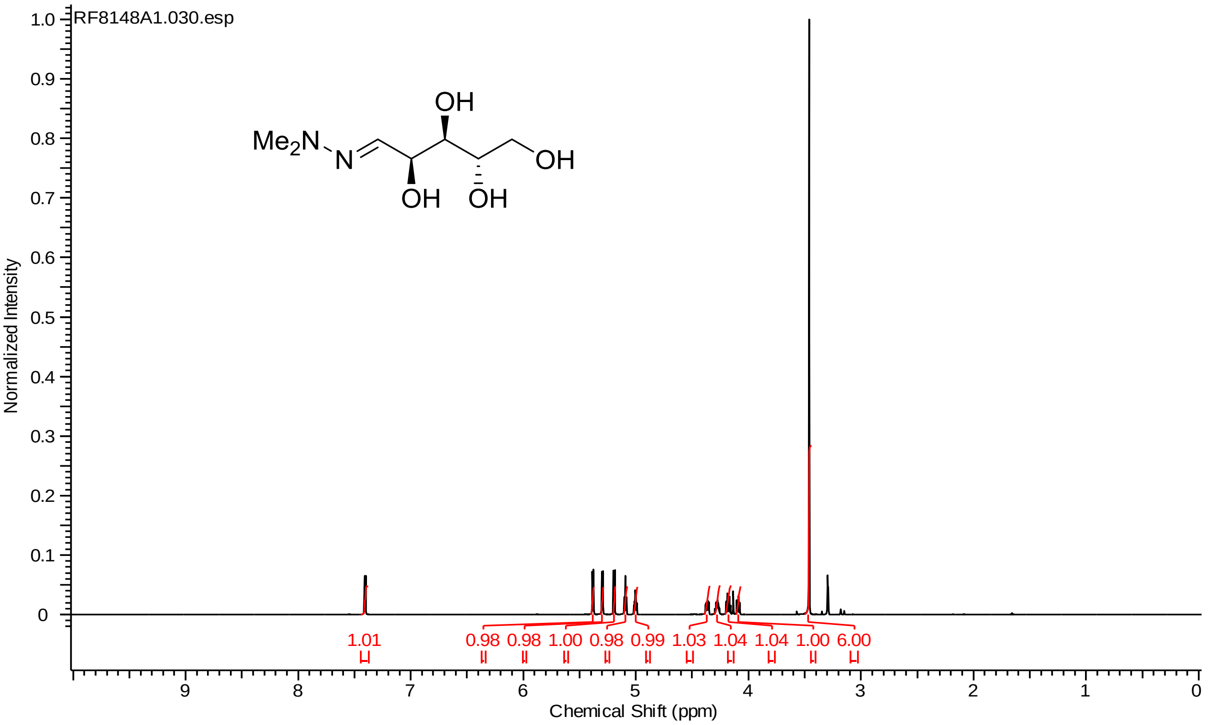

^{13}C NMR (150 MHz, DMSO-d_6)

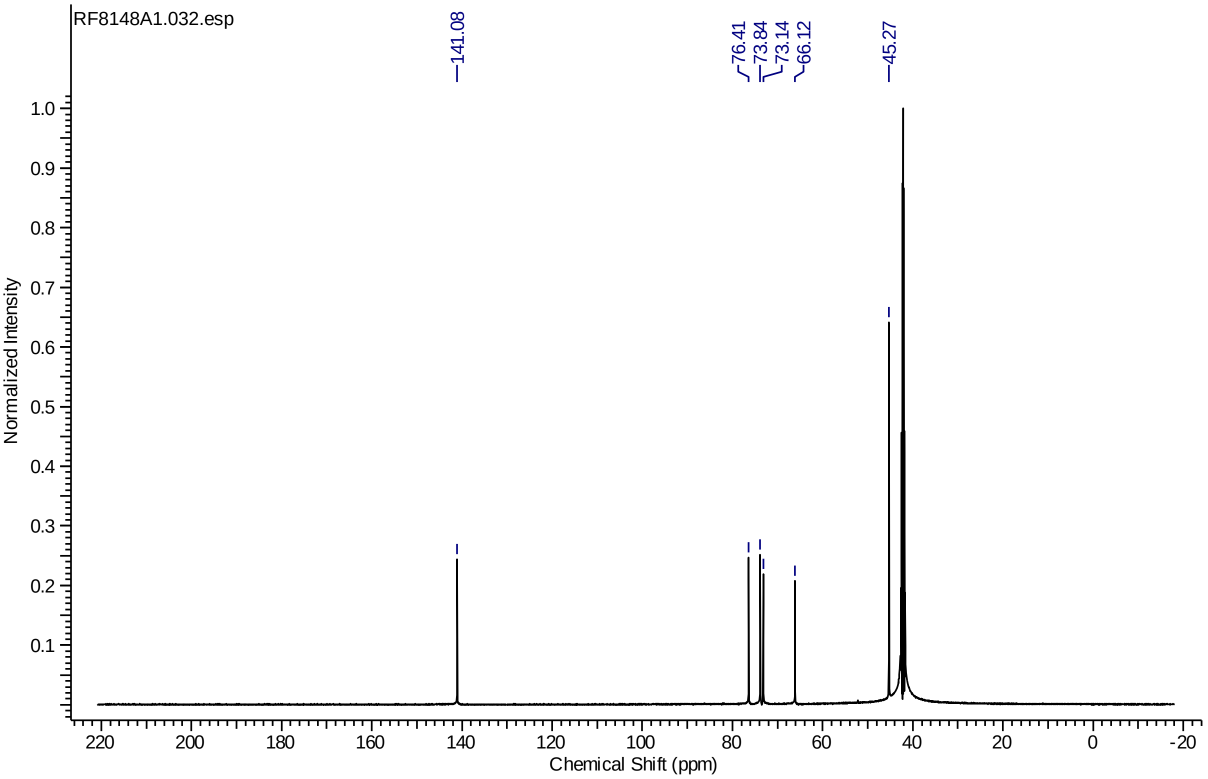

(2*R*,3*S*,4*S*,*E*)-5-(2,2-Dimethylhydrazono)pentane-1,2,3,4-tetraol 2b

^{1}H NMR (600 MHz, DMSO-d$_6$)

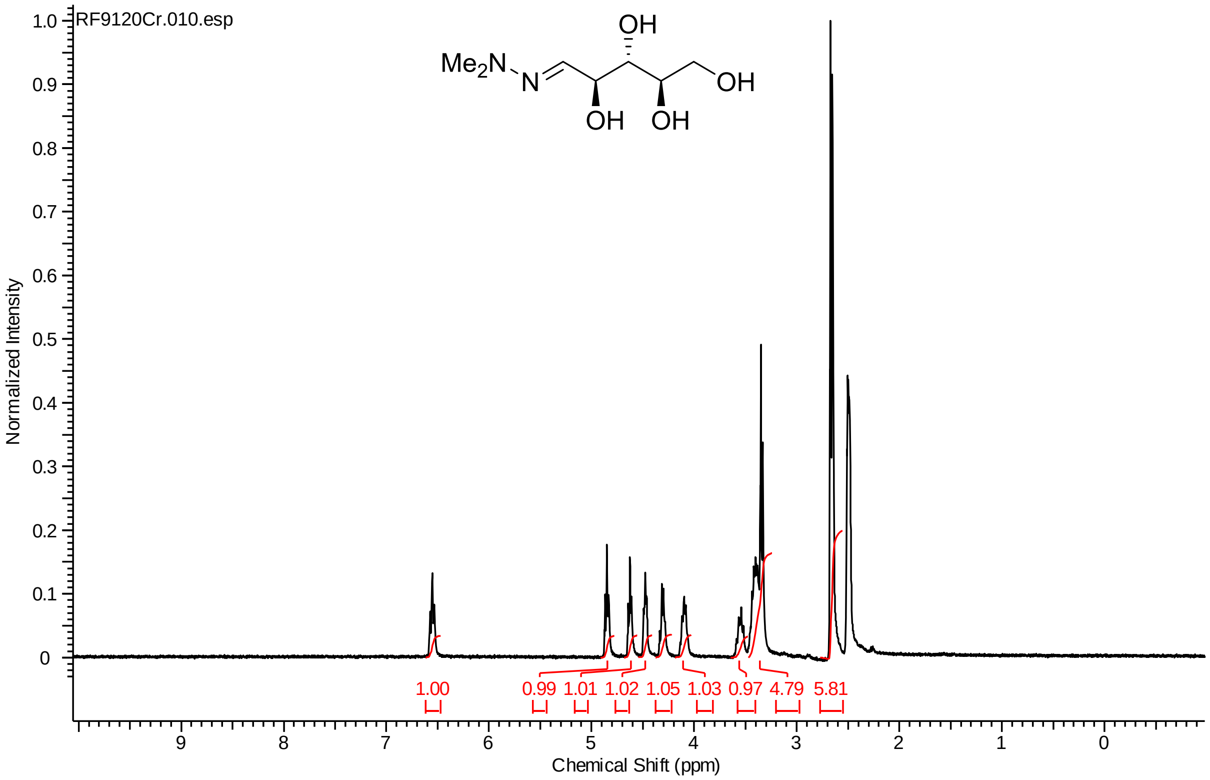

^{13}C NMR (150 MHz, DMSO-d$_6$)

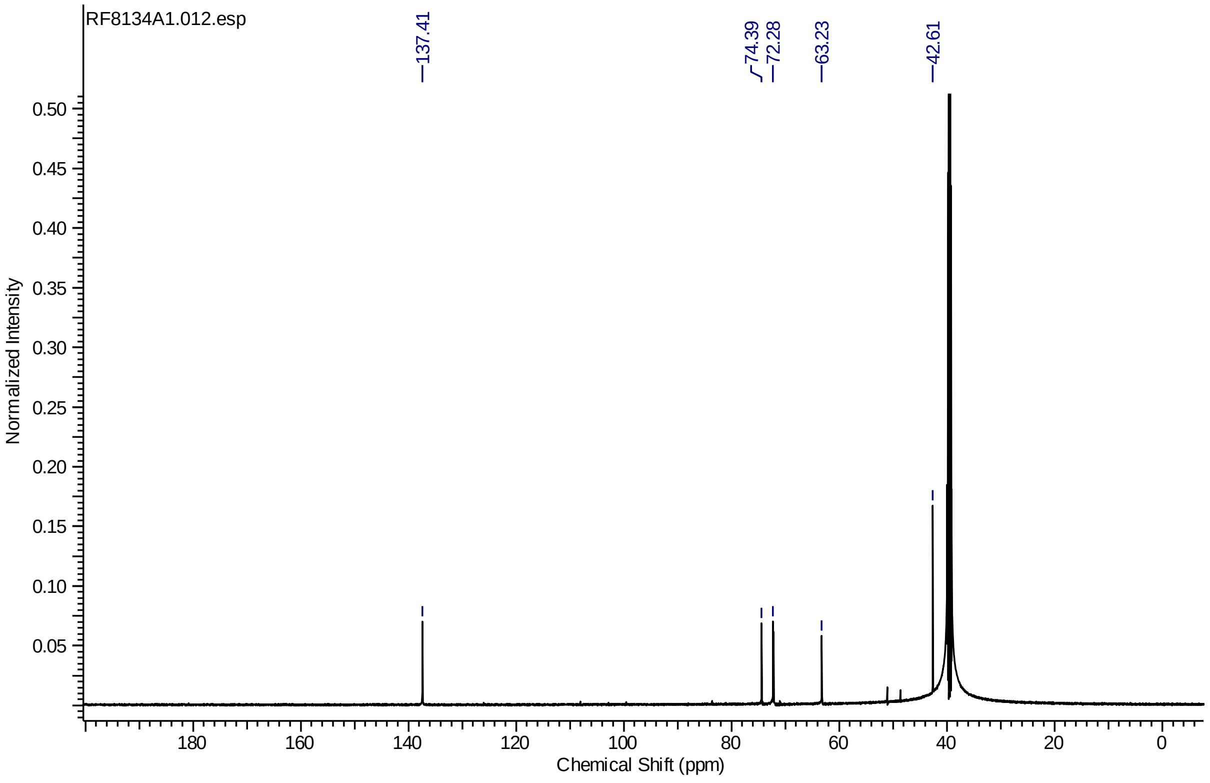

(2*R*,3*R*,4*R*,*E*)-5-(2,2-Dimethylhydrazono)pentane-1,2,3,4-tetraol 2c

^{1}H NMR (600 MHz, DMSO-d$_6$)

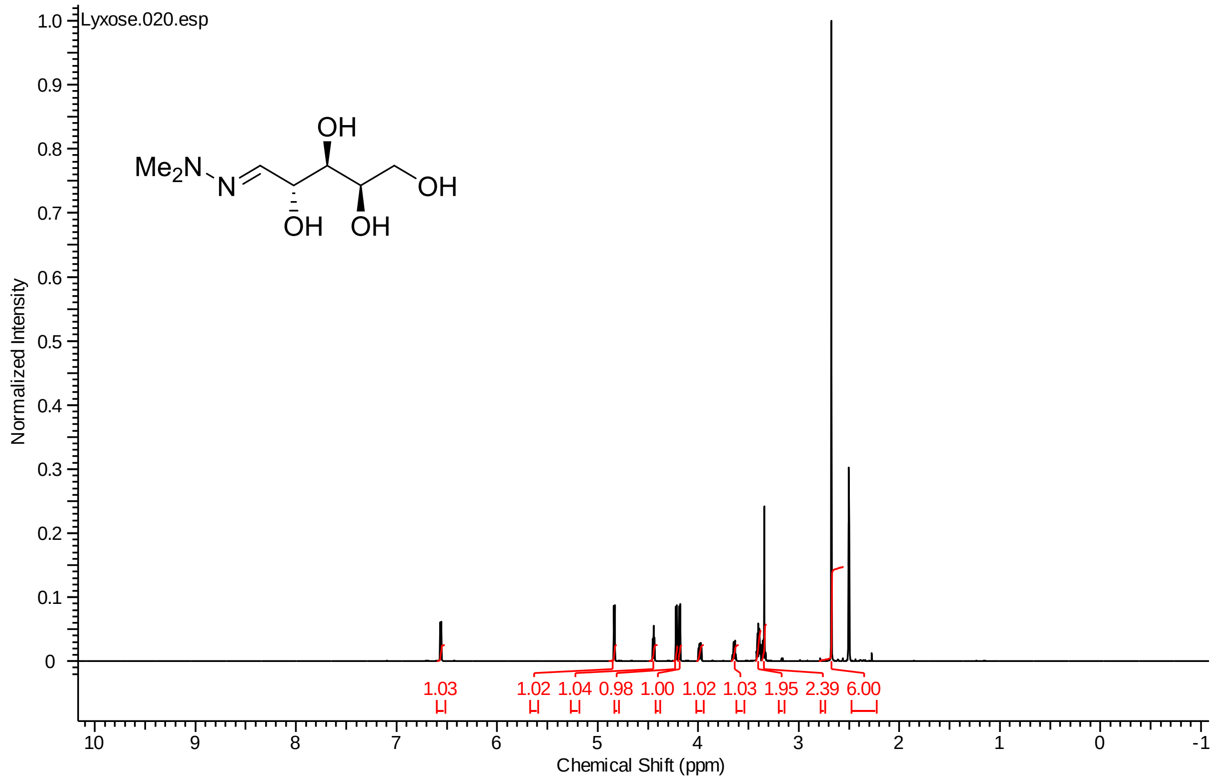

^{13}C NMR (150 MHz, DMSO-d$_6$)

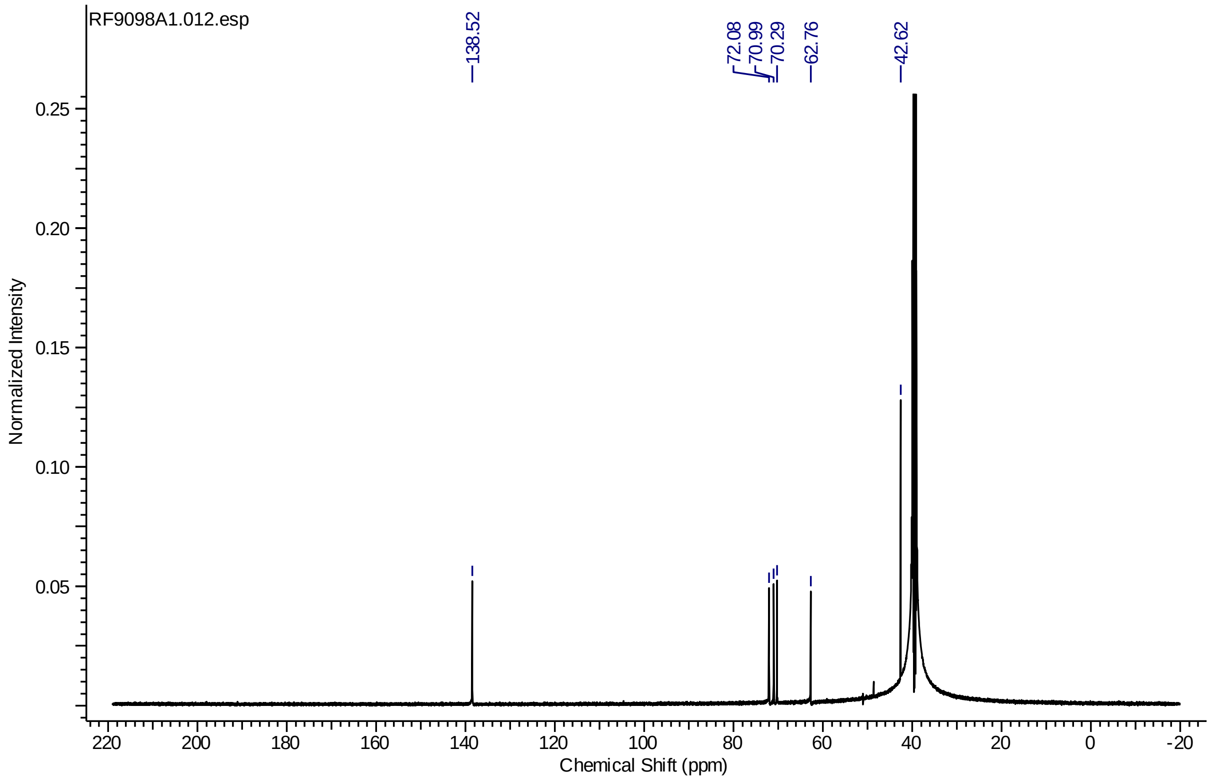

(2*S*,3*S*,4*S*,5*S*,*E*)-1-(2,2-Dimethylhydrazono)hexane-2,3,4,5-tetraol 2e

^{1}H NMR (600 MHz, DMSO-d$_6$)

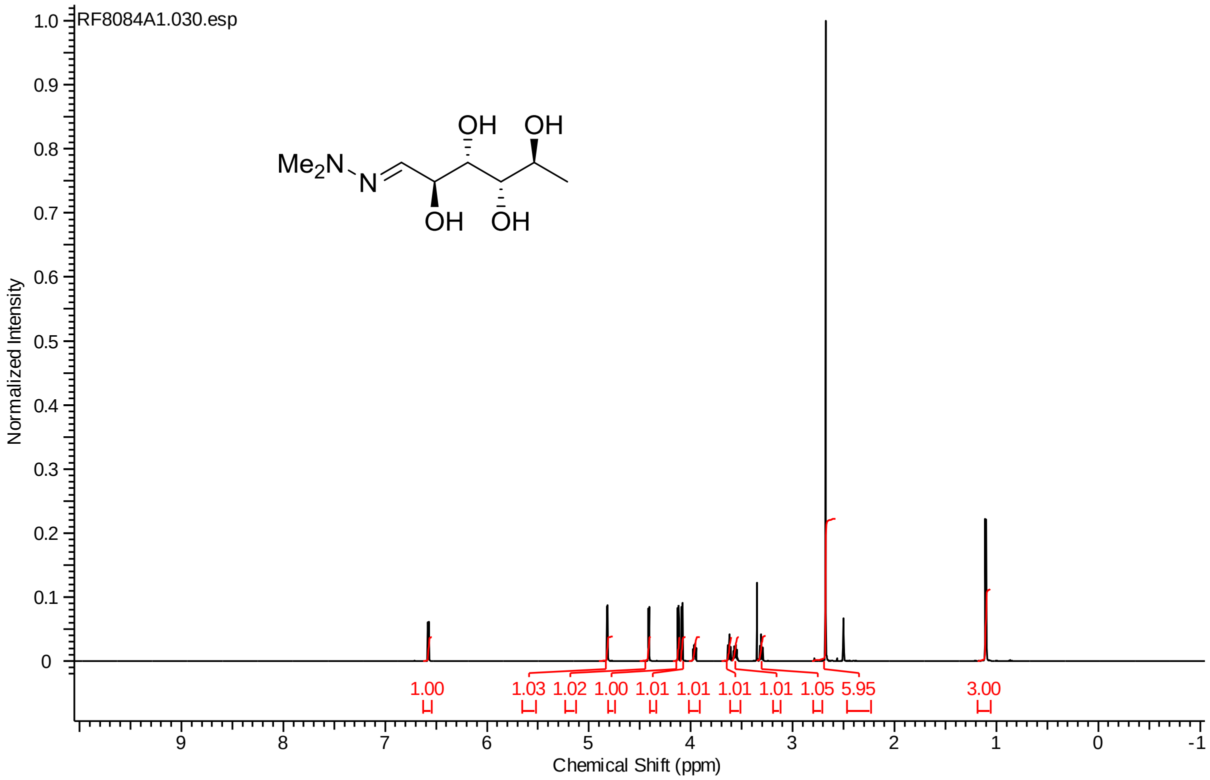

^{13}C NMR (150 MHz, DMSO-d$_6$)

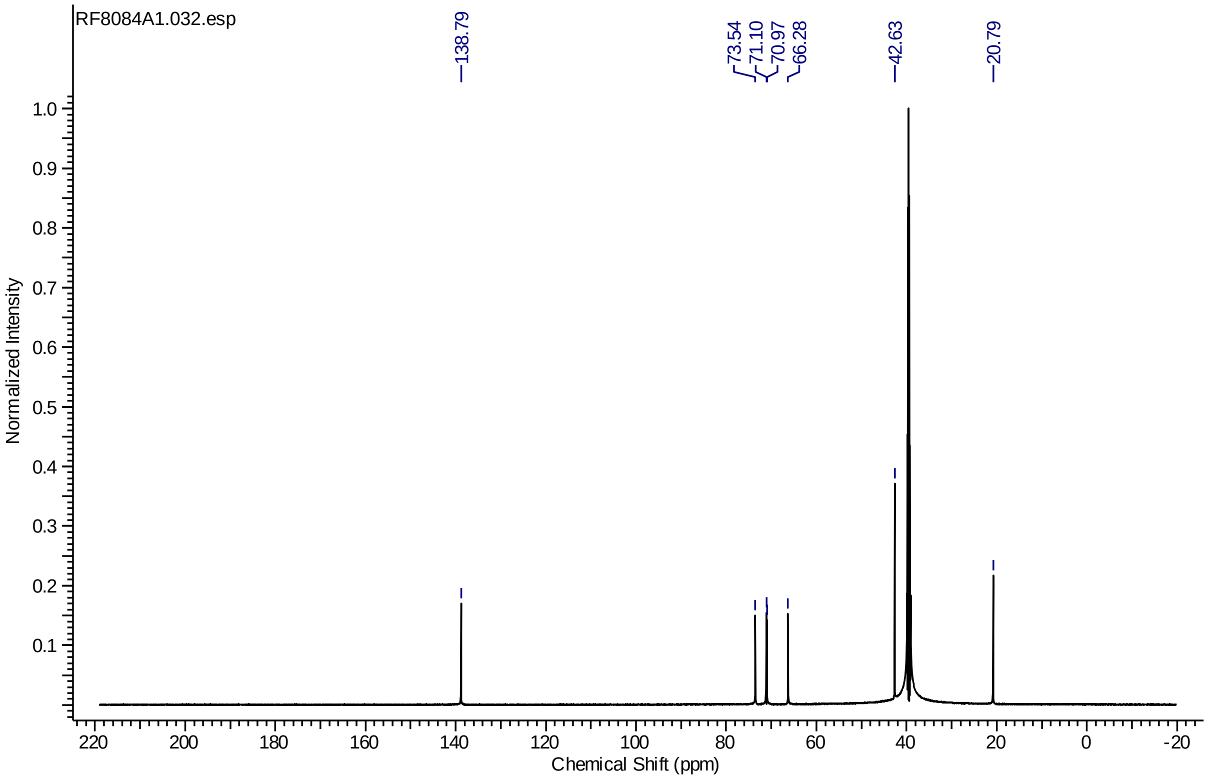

(2*R*,3*S*,4*S*)-2-((*E*)-(2,2-Dimethylhydrazono)methyl)tetrahydrofuran-3,4-diol *anti*-3a and (2*S*,3*S*,4*S*)-2-((*E*)-(2,2-Dimethylhydrazono)methyl)tetrahydrofuran-3,4-diol *syn*-3a

^{1}H NMR (600 MHz, MeOH-d$_4$)

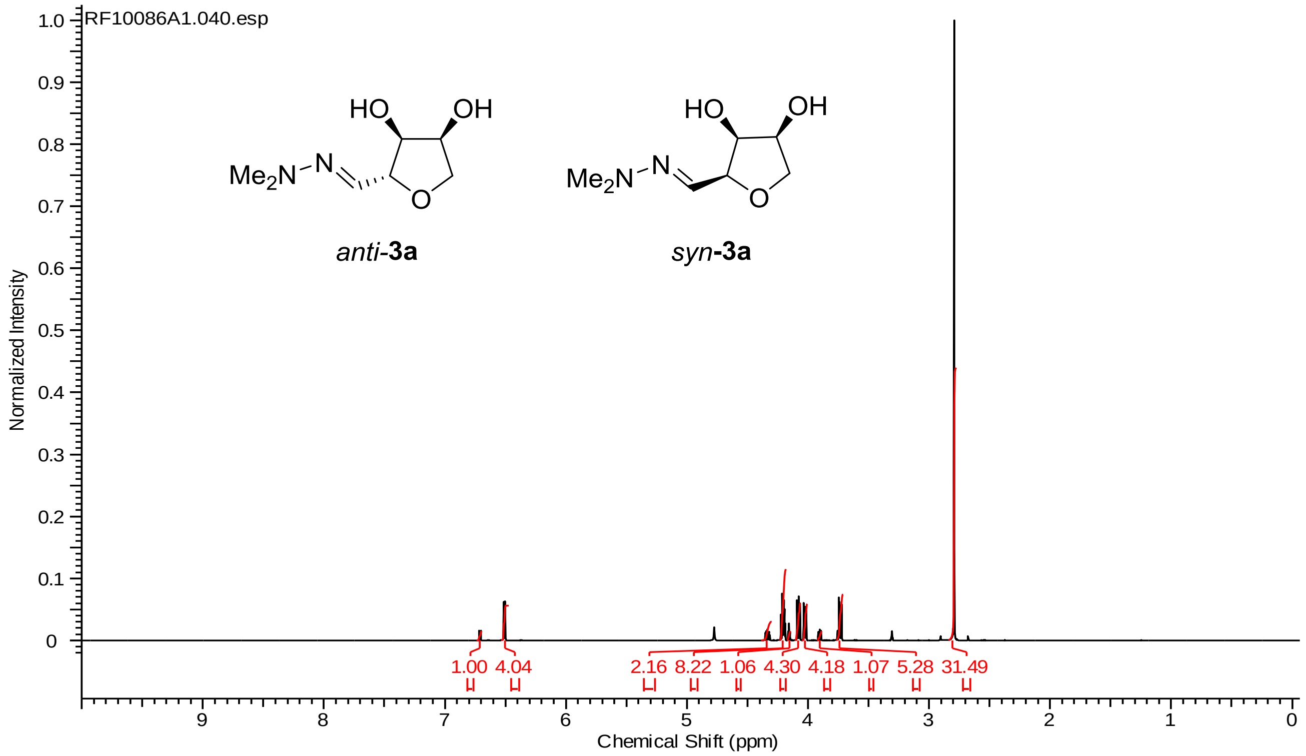

^{13}C NMR (150 MHz, MeOH-d$_4$)

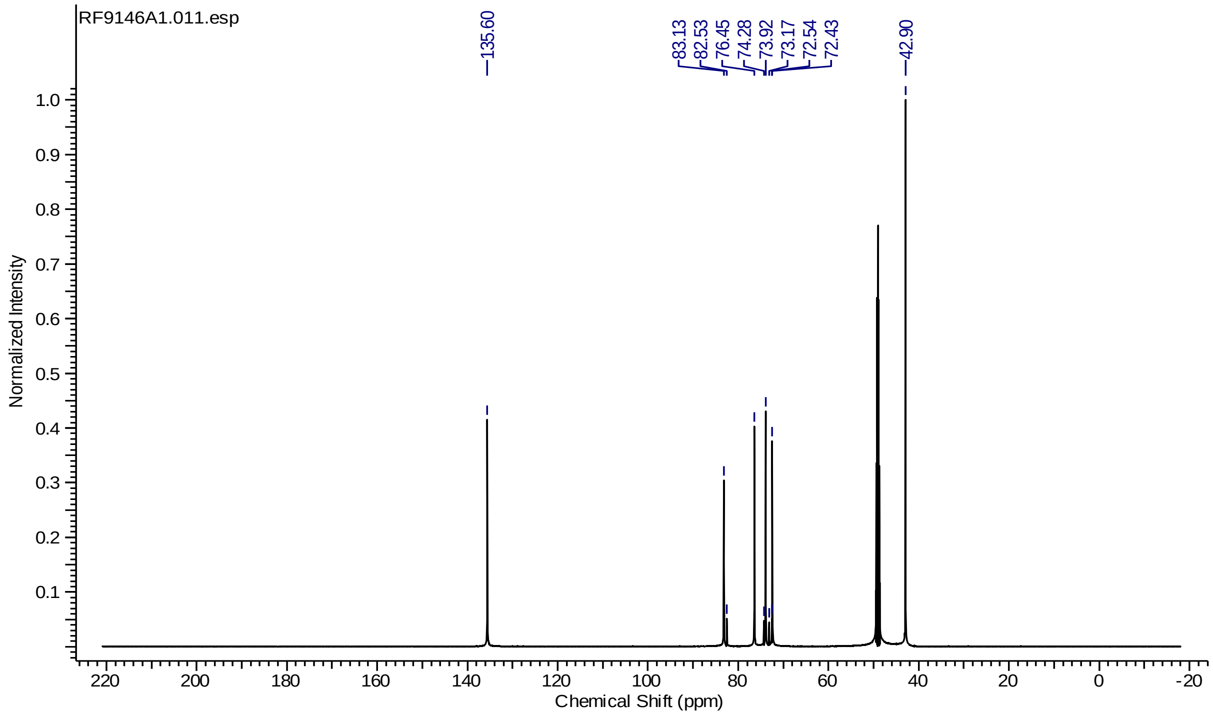

(2*R*,3*S*,4*S*)-2-((*E*)-(2,2-Dimethylhydrazono)methyl)tetrahydrofuran-3,4-diol *anti*-3a

^{1}H NMR (600 MHz, MeOH-d_4)

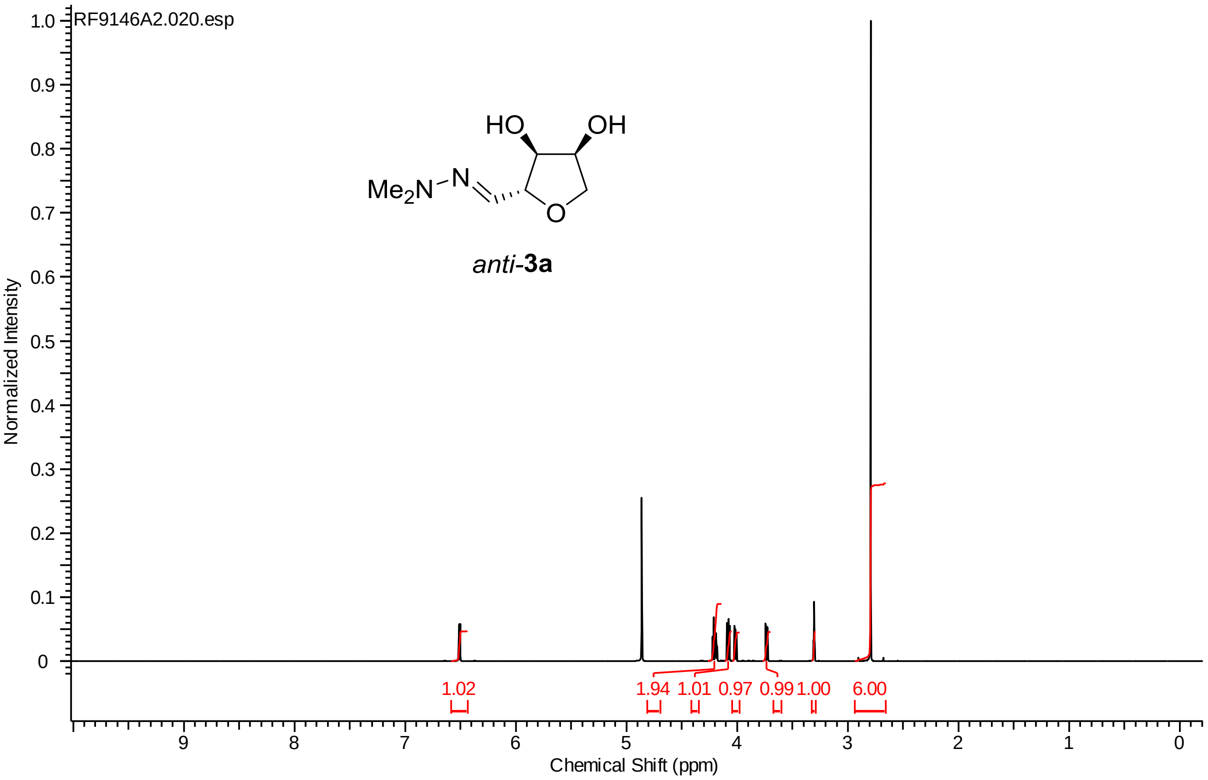

^{13}C NMR (150 MHz, MeOH-d_4)

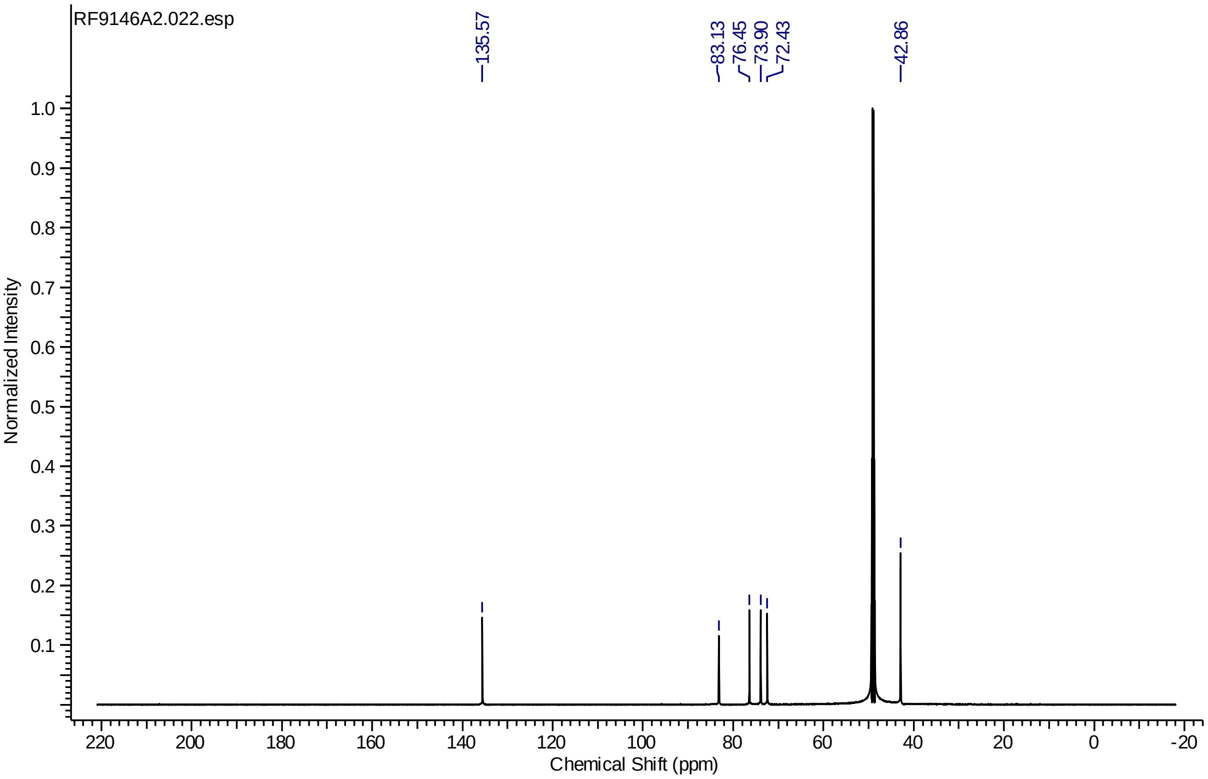

(2*R*,3*R*,4*S*)-2-((*E*)-(2,2-Dimethylhydrazono)methyl)tetrahydrofuran-3,4-diol *anti*-3b and ((2*S*,3*R*,4*S*)-2-((*E*)-(2,2-Dimethylhydrazono)methyl)tetrahydrofuran-3,4-diol *syn*-3b

^{1}H NMR (600 MHz, MeOH-d$_4$)

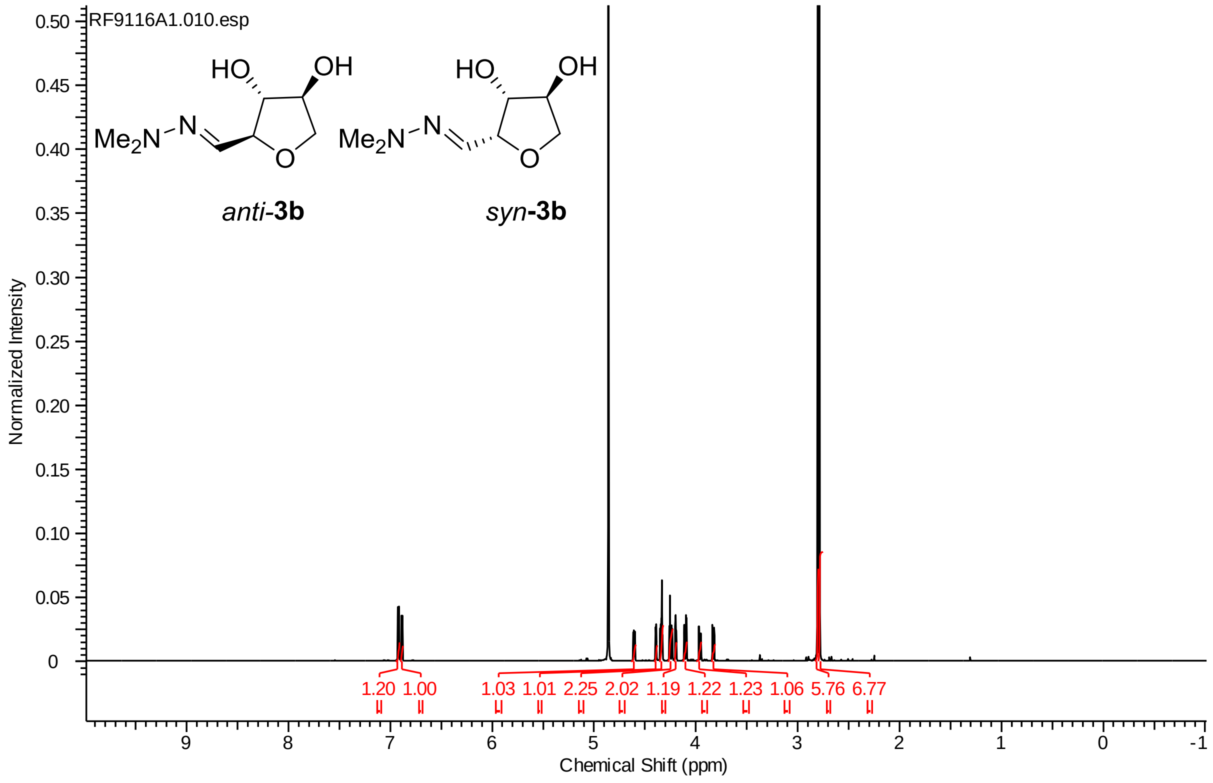

^{13}C NMR (150 MHz, MeOH-d$_4$)

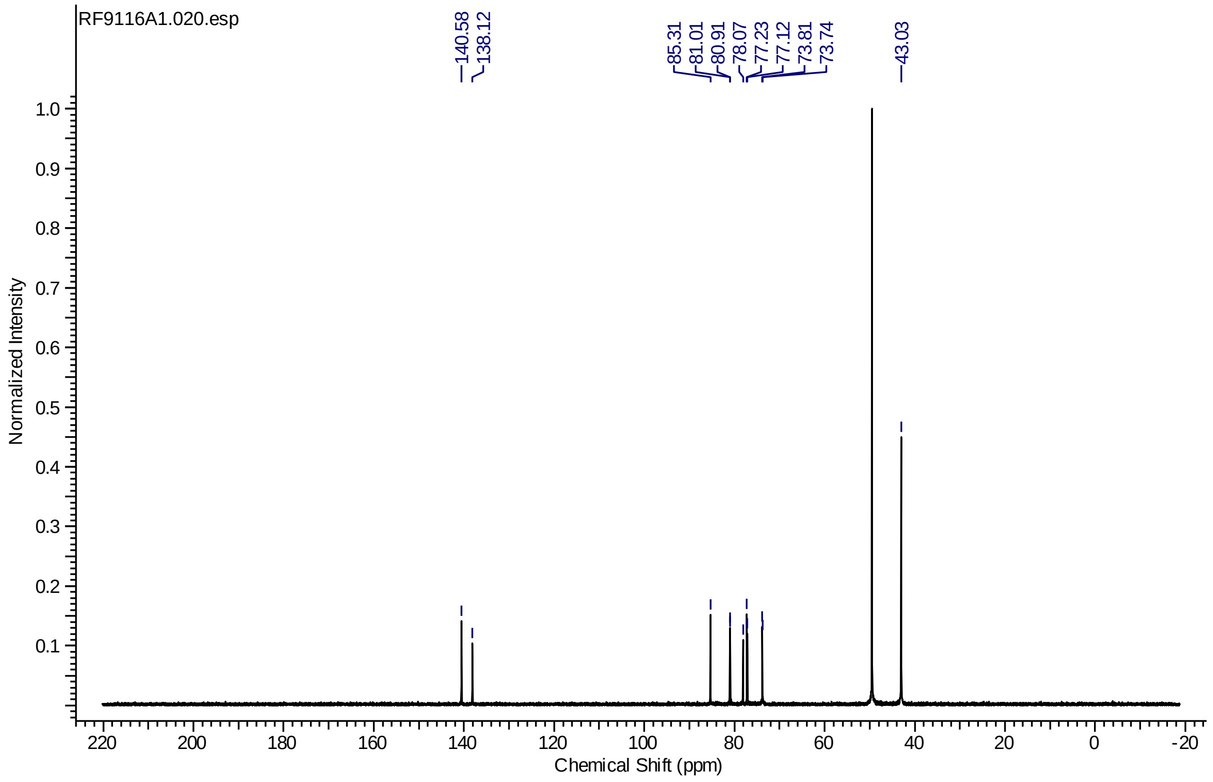

(2*S*,3*R*,4*R*,5*S*)-2-((*E*)-(2,2-Dimethylhydrazono)methyl)-5-methyltetrahydrofuran-3,4-diol *anti*-3c and (2*R*,3*R*,4*R*,5*S*)-2-((*E*)-(2,2-Dimethylhydrazono)methyl)-5-methyltetrahydrofuran-3,4-diol *syn*-3c

^{1}H NMR (600 MHz, MeOH-d$_4$)

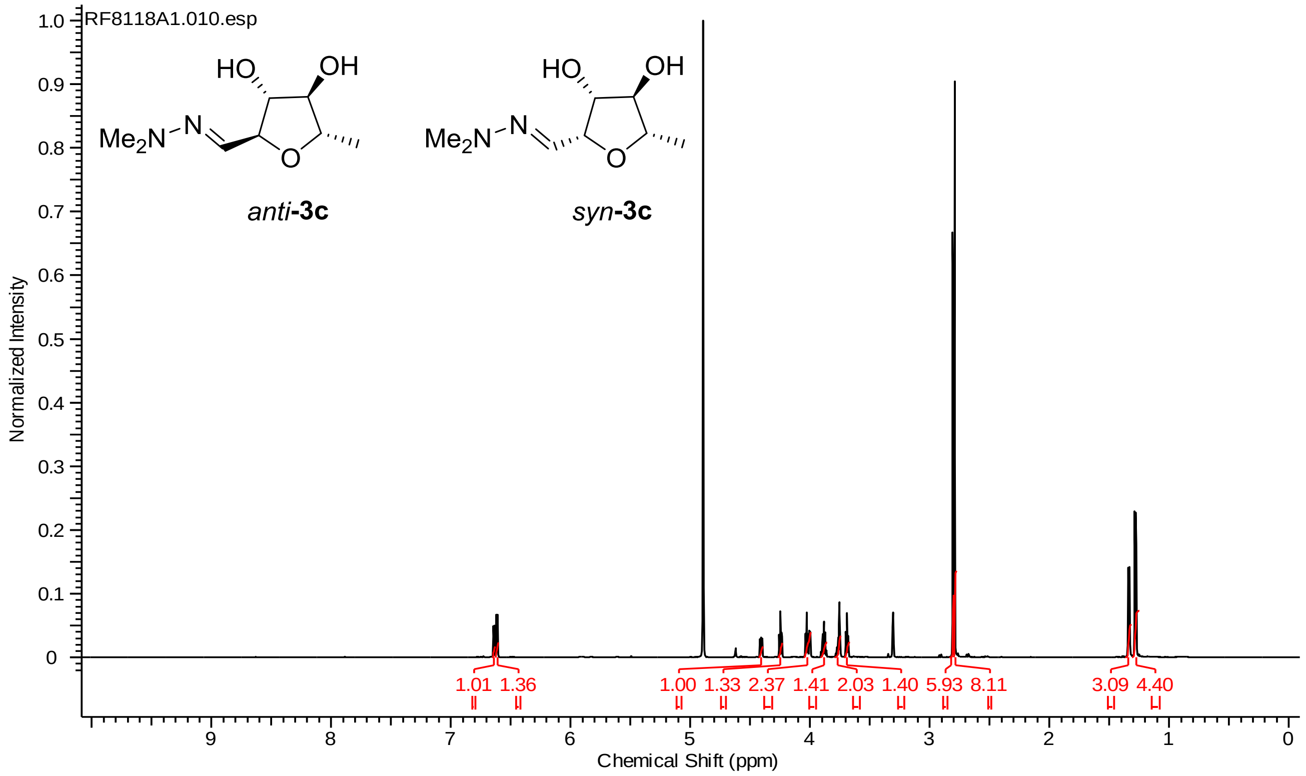

^{13}C NMR (150 MHz, MeOH-d$_4$)

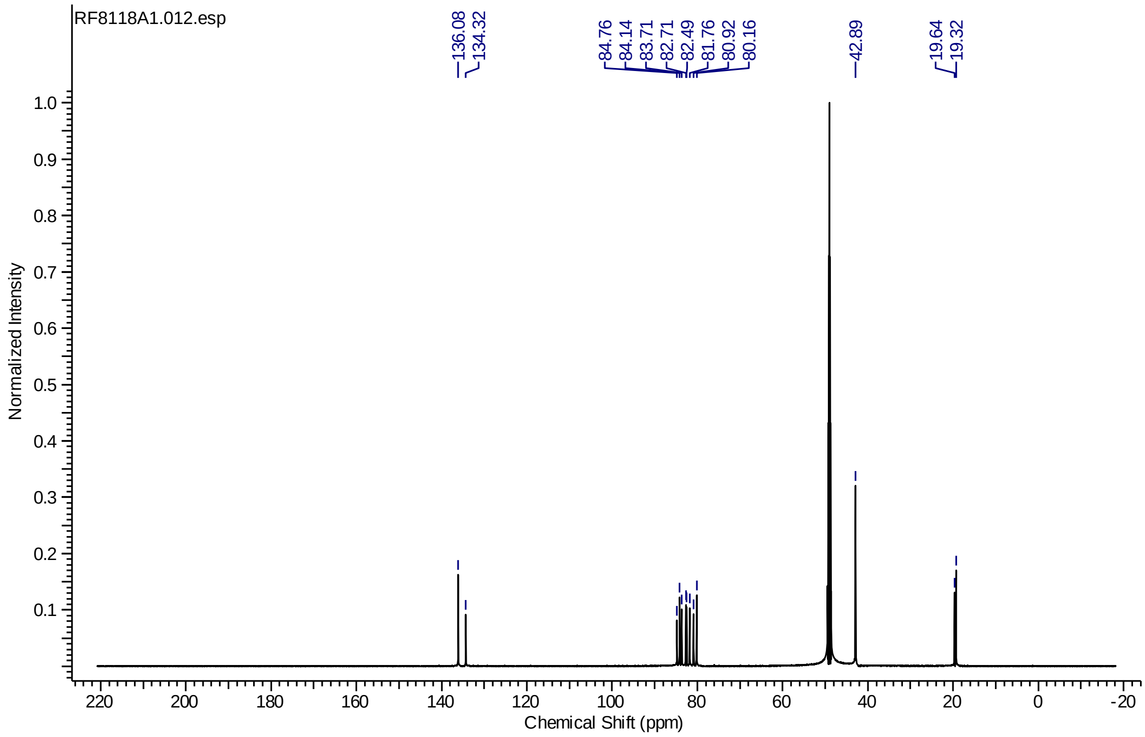

***tert*-Butyl (((2*R*,3*S*,4*S*)-3,4-dihydroxytetrahydrofuran-2-yl)methyl)carbamate 4**

^{1}H NMR (600 MHz, $CDCl_3$)

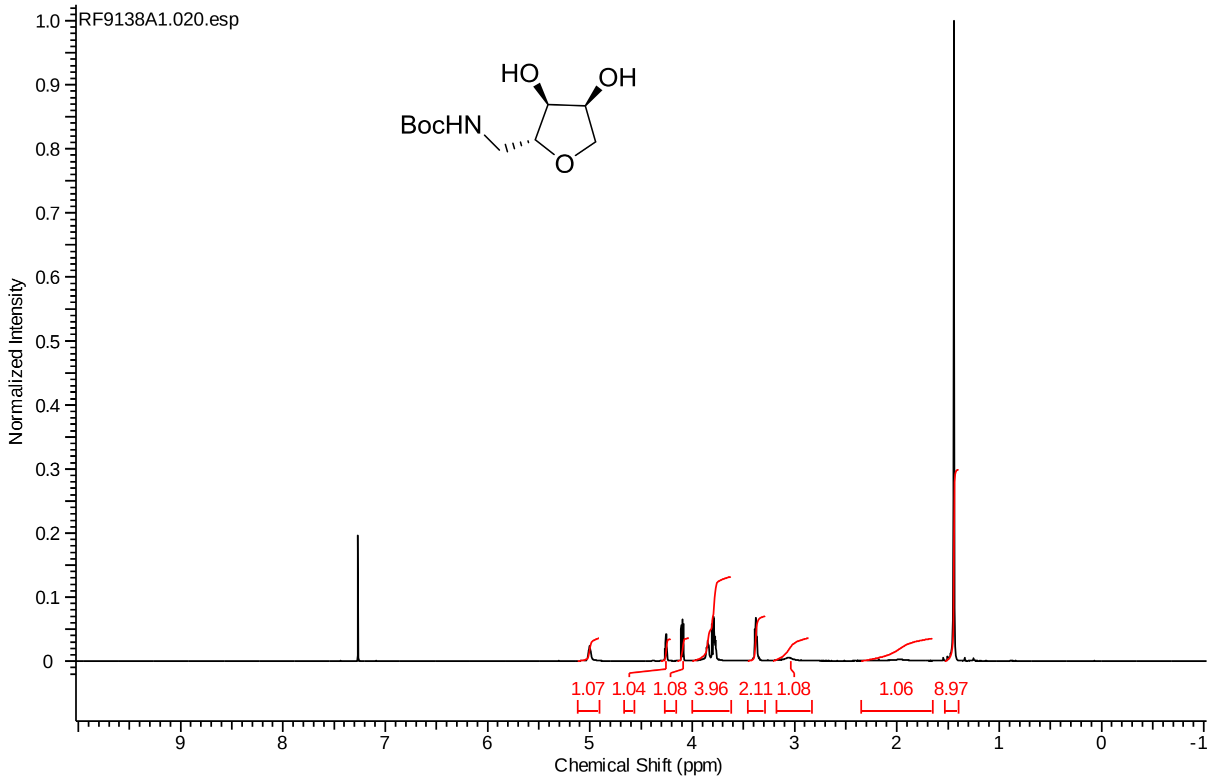

^{13}C NMR (150 MHz, $CDCl_3$)

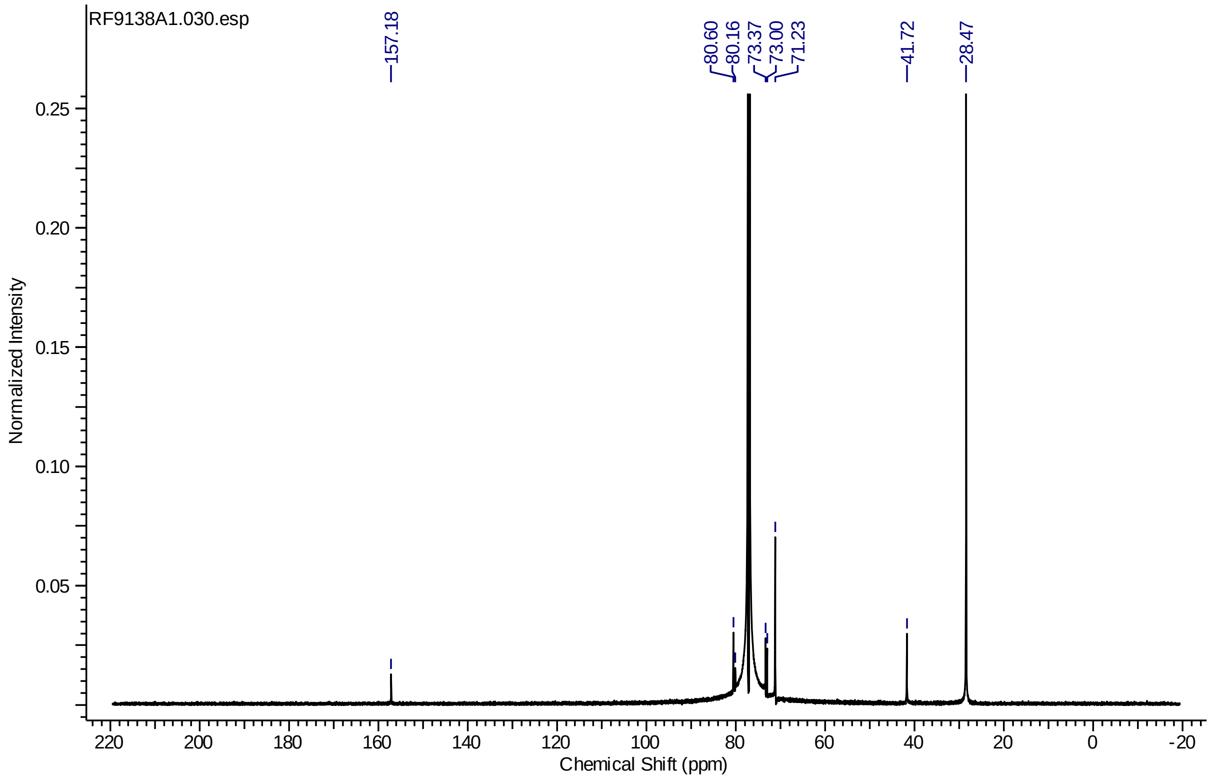

(2*S*,3*S*,4*S*)-2-(Dihydroxymethyl)tetrahydrofuran-3,4-diol *anti*-5 and (2*R*,3*S*,4*S*)-2-(Dihydroxymethyl)tetrahydrofuran-3,4-diol *syn*-5

^{1}H NMR (600 MHz, D_2O)

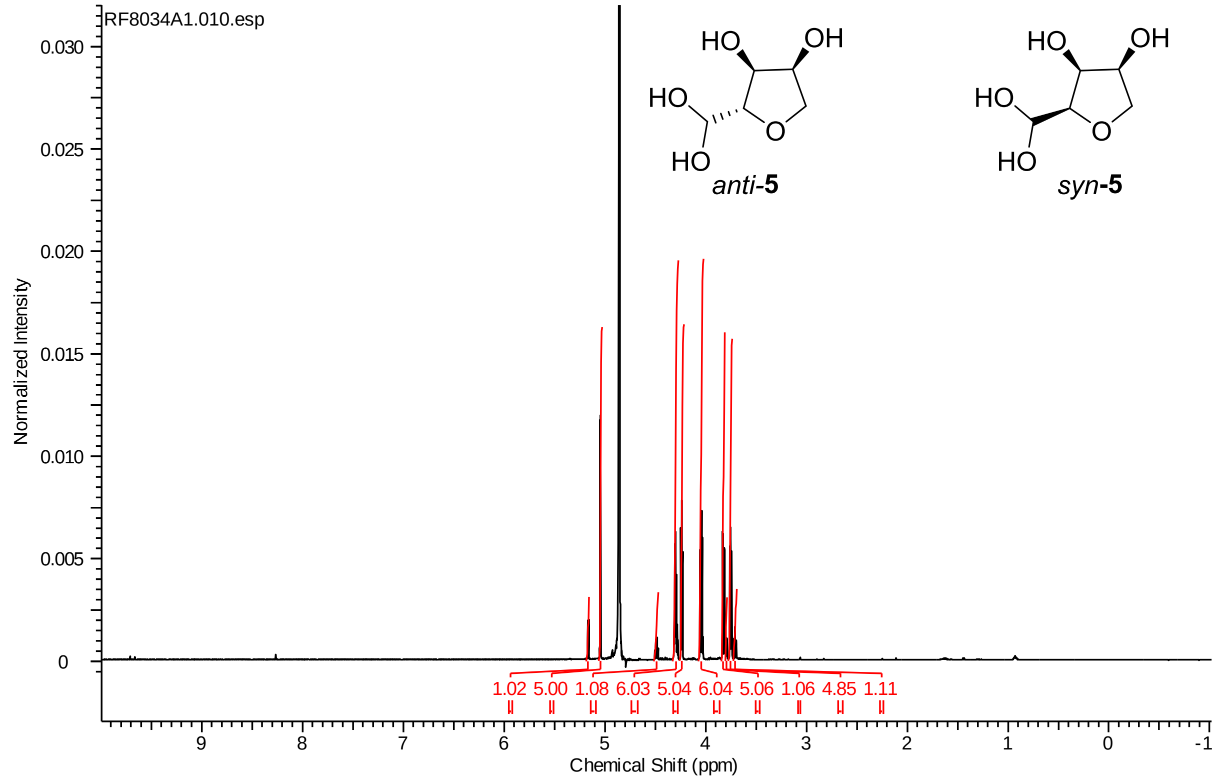

^{13}C NMR (150 MHz, D_2O with MeOH standard)

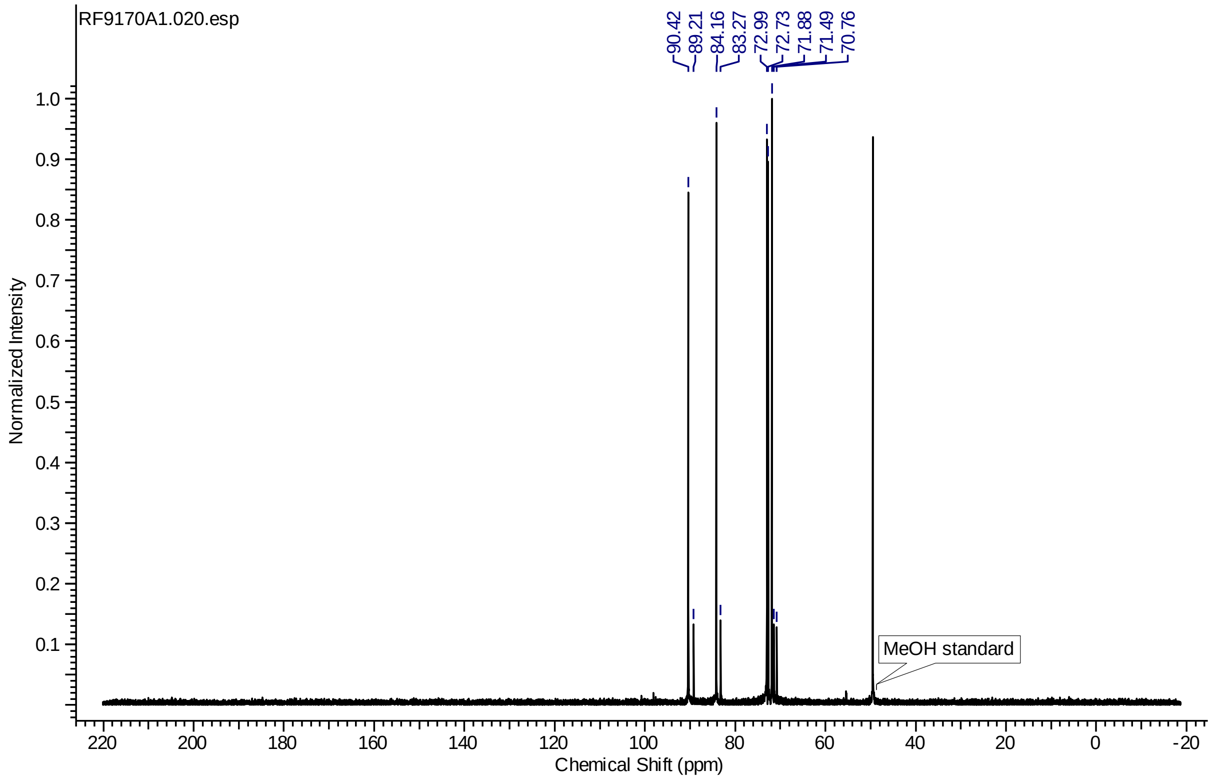

^{1}H NMR (600 MHz, MeOH-d_4)

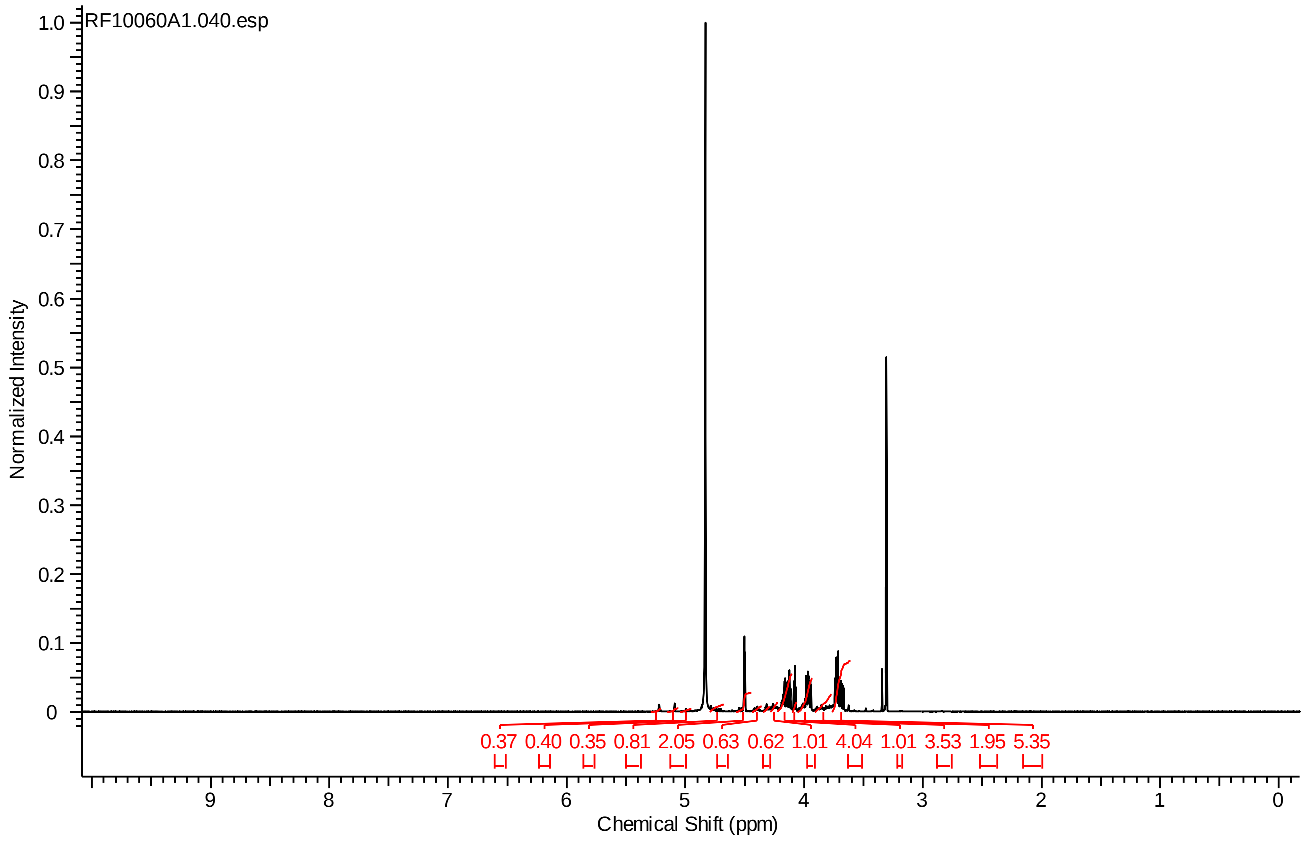

^{13}C NMR (150 MHz, MeOH-d_4)

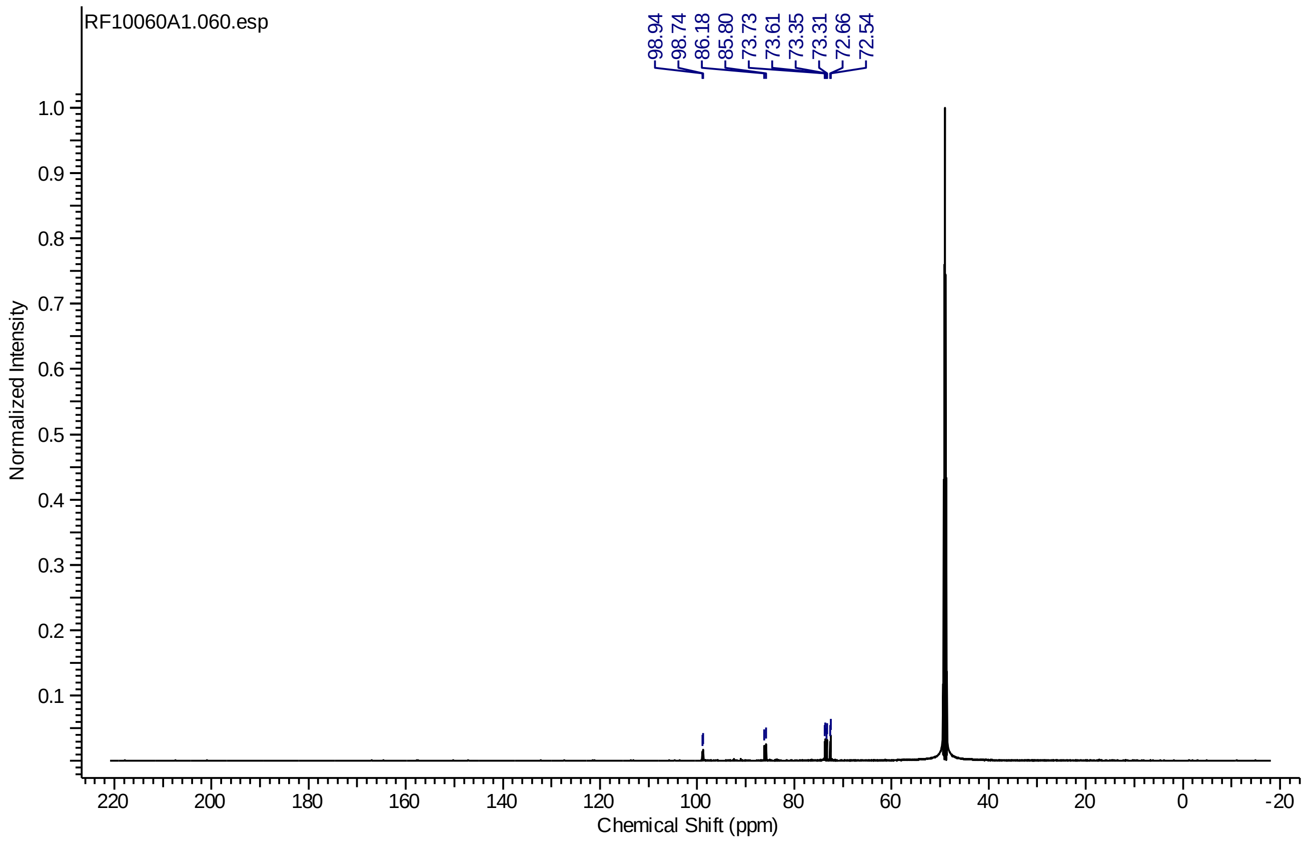

^{1}H NMR (600 MHz, DMSO-d_6)

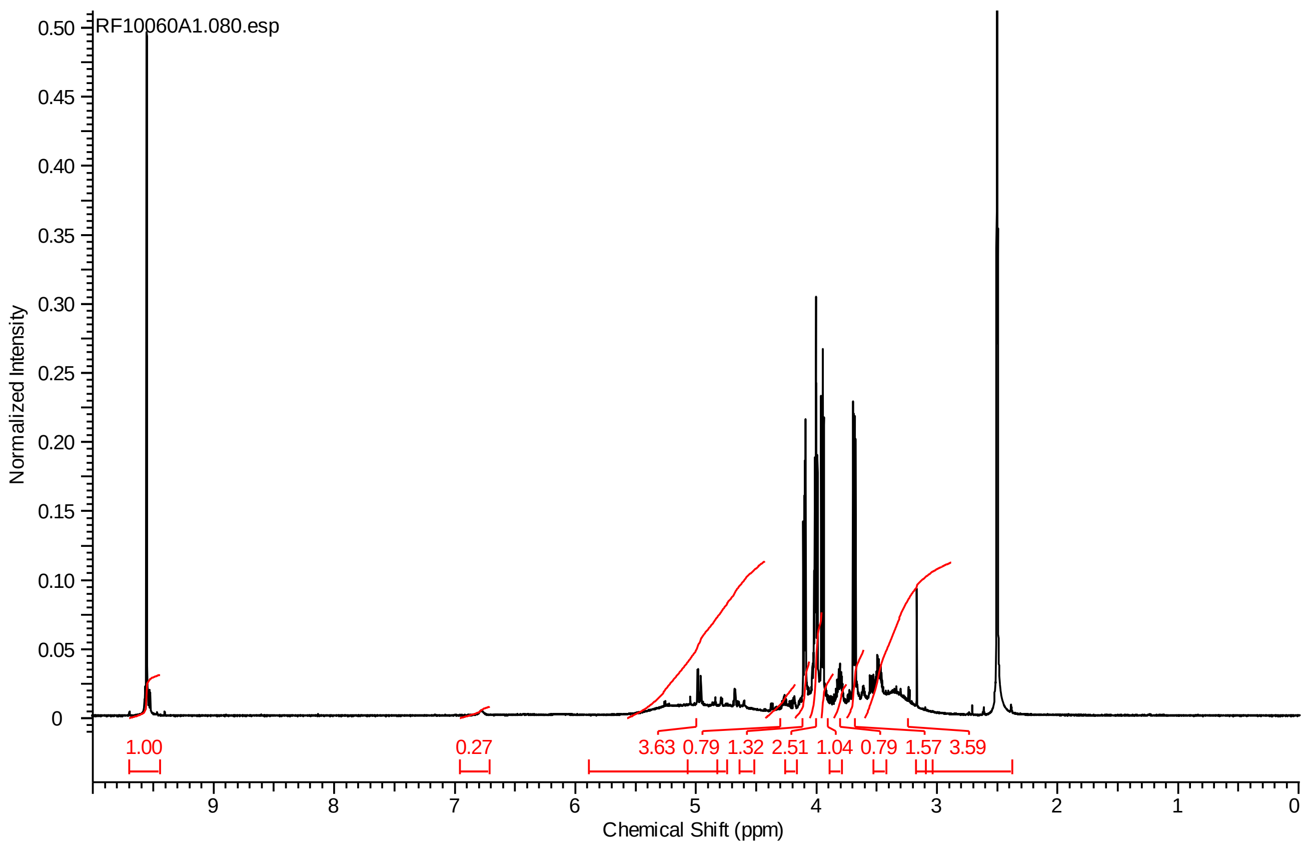

^{13}C NMR (150 MHz, MeOH-d_4)

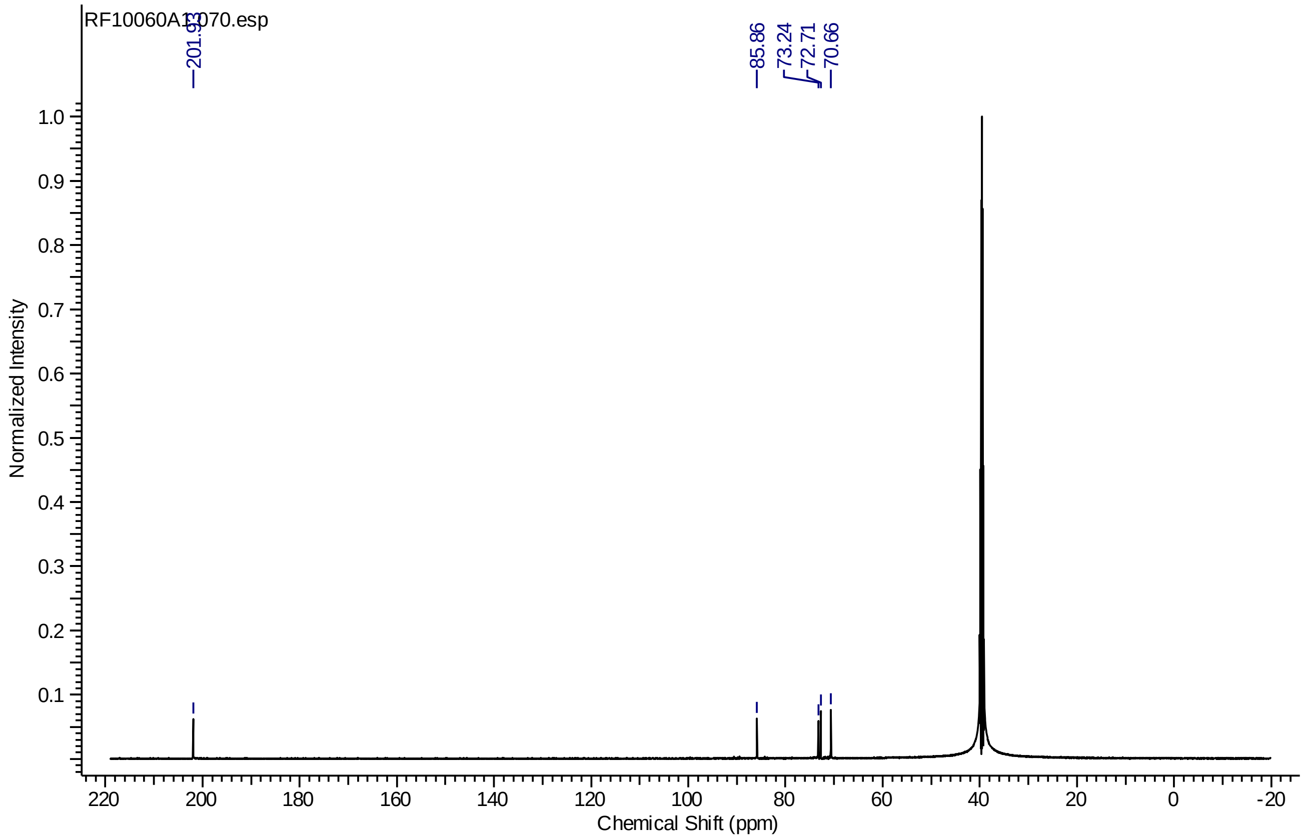

((2*S*,3*R*,4*S*)-2-((*E*)-(2,2-Dimethylhydrazono)methyl)tetrahydrofuran-3,4-diol 6 and (2*R*,3*R*,4*S*)-2-((*E*)-(2,2-Dimethylhydrazono)methyl)tetrahydrofuran-3,4-diol 6

^{1}H NMR (600 MHz, D_2O)

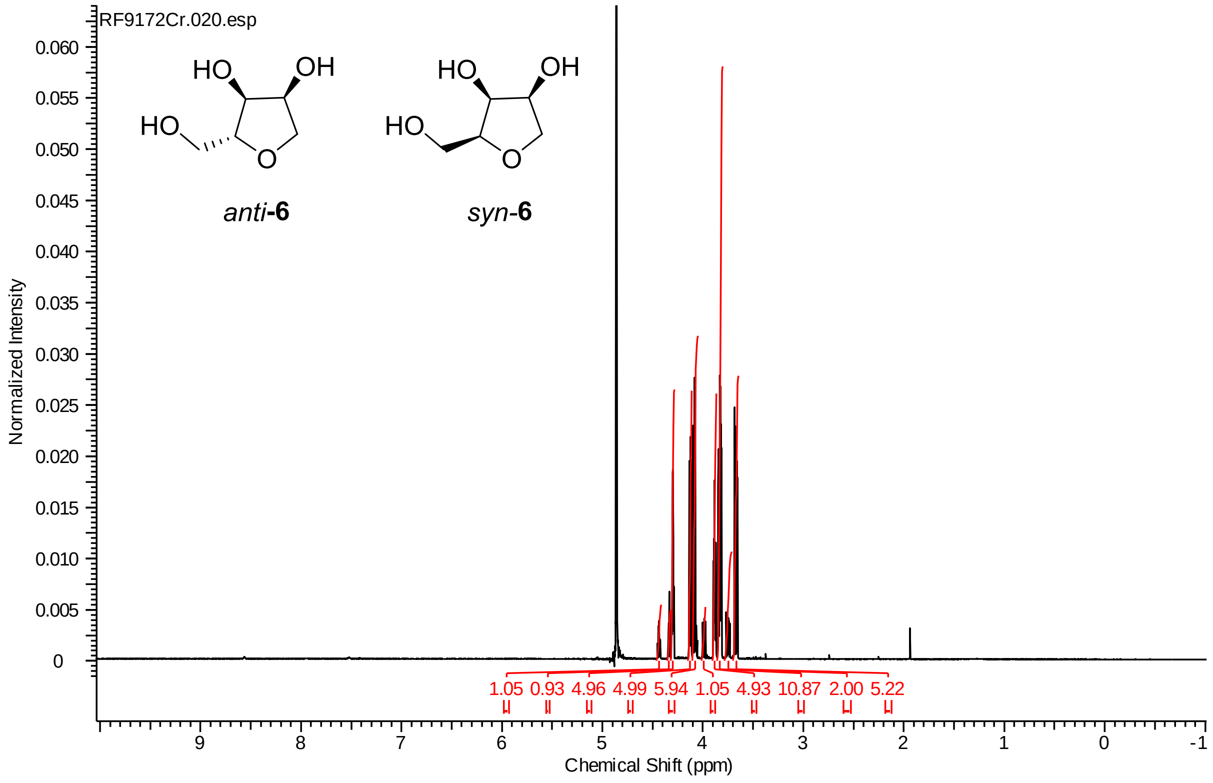

^{13}C NMR (150 MHz, D_2O with MeOH standard)

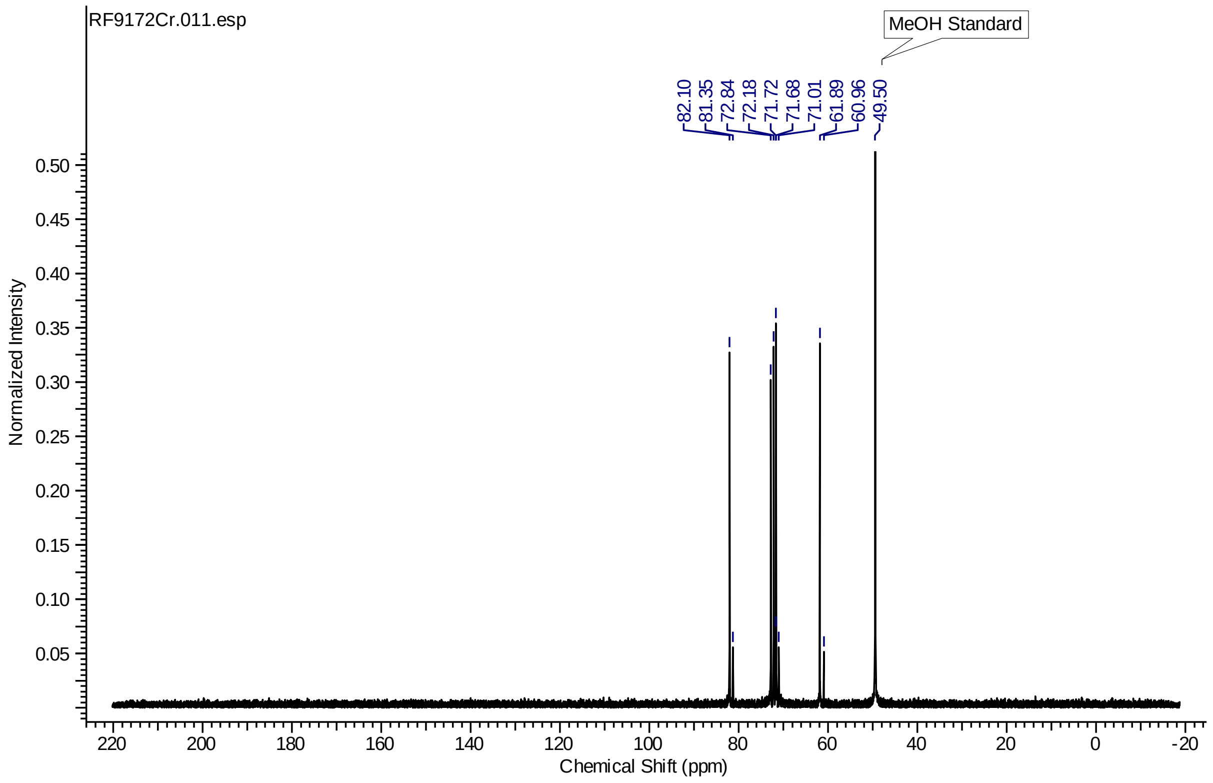

***tert*-Butyl butyl(((2*R*,3*S*,4*S*)-3,4-dihydroxytetrahydrofuran-2-yl)methyl)carbamate *anti*-7 and *tert*-Butyl butyl(((2*S*,3*S*,4*S*)-3,4-dihydroxytetrahydrofuran-2-yl)methyl)carbamate *syn*-7**

^{1}H NMR (400 MHz, DMSO-d$_6$, 80 °C)

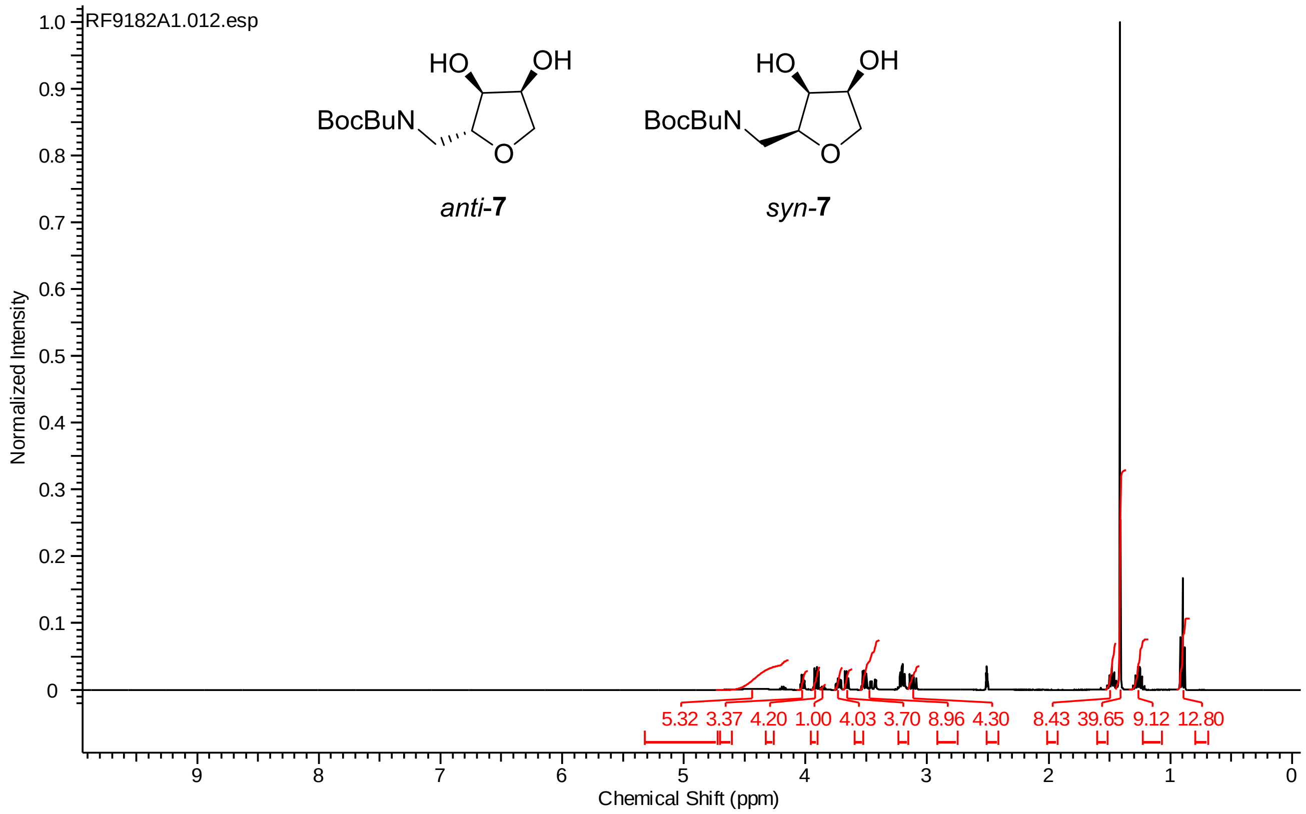

^{13}C NMR (150 MHz, DMSO-d$_6$)

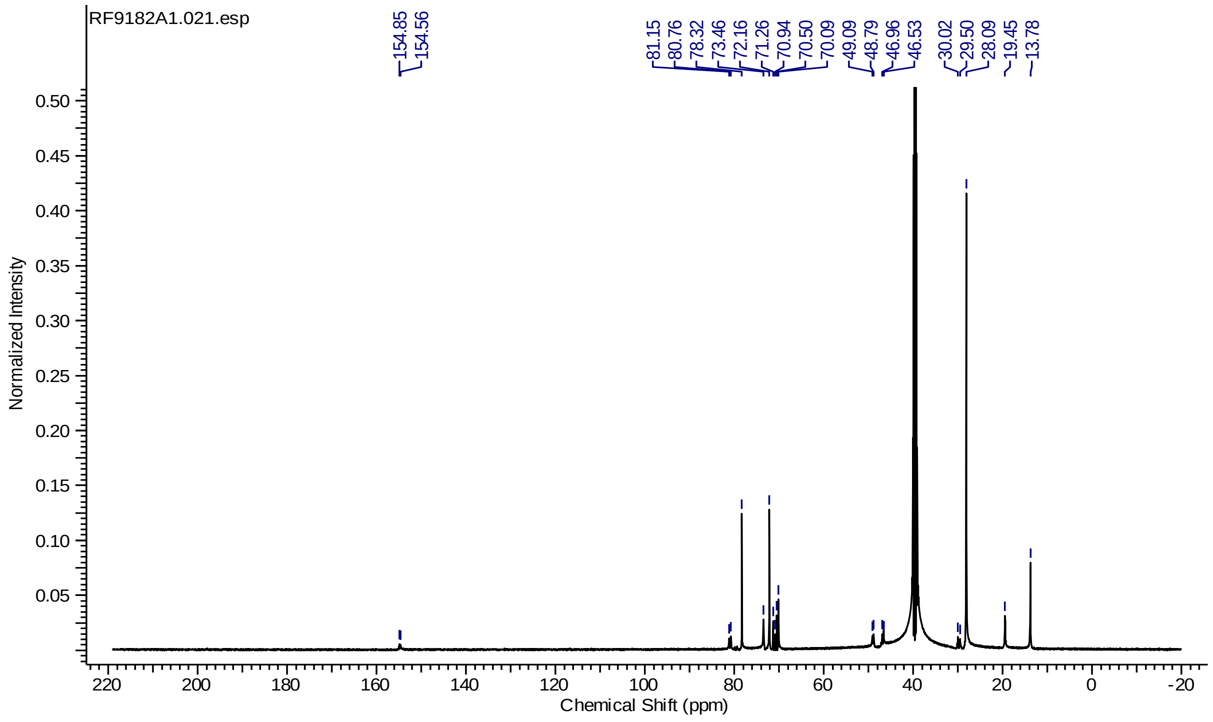

Methyl (*E*)-3-((2*R*,3*S*,4*S*)-3,4-dihydroxytetrahydrofuran-2-yl)acrylate *anti*-8 and Methyl (*E*)-3-((2*S*,3*S*,4*S*)-3,4-dihydroxytetrahydrofuran-2-yl)acrylate *syn*-8

^{1}H NMR (600 MHz, MeOH-d$_4$)

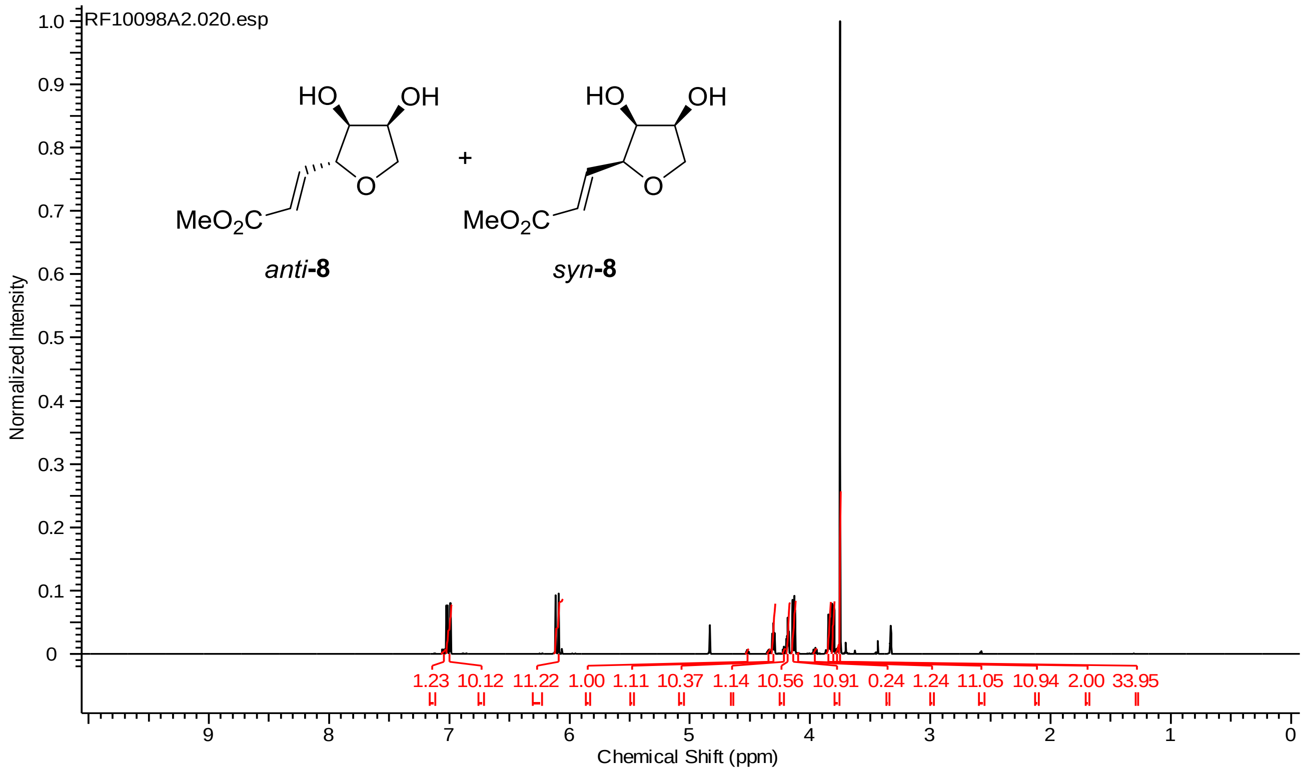

^{13}C NMR (150 MHz, MeOH-d$_4$)

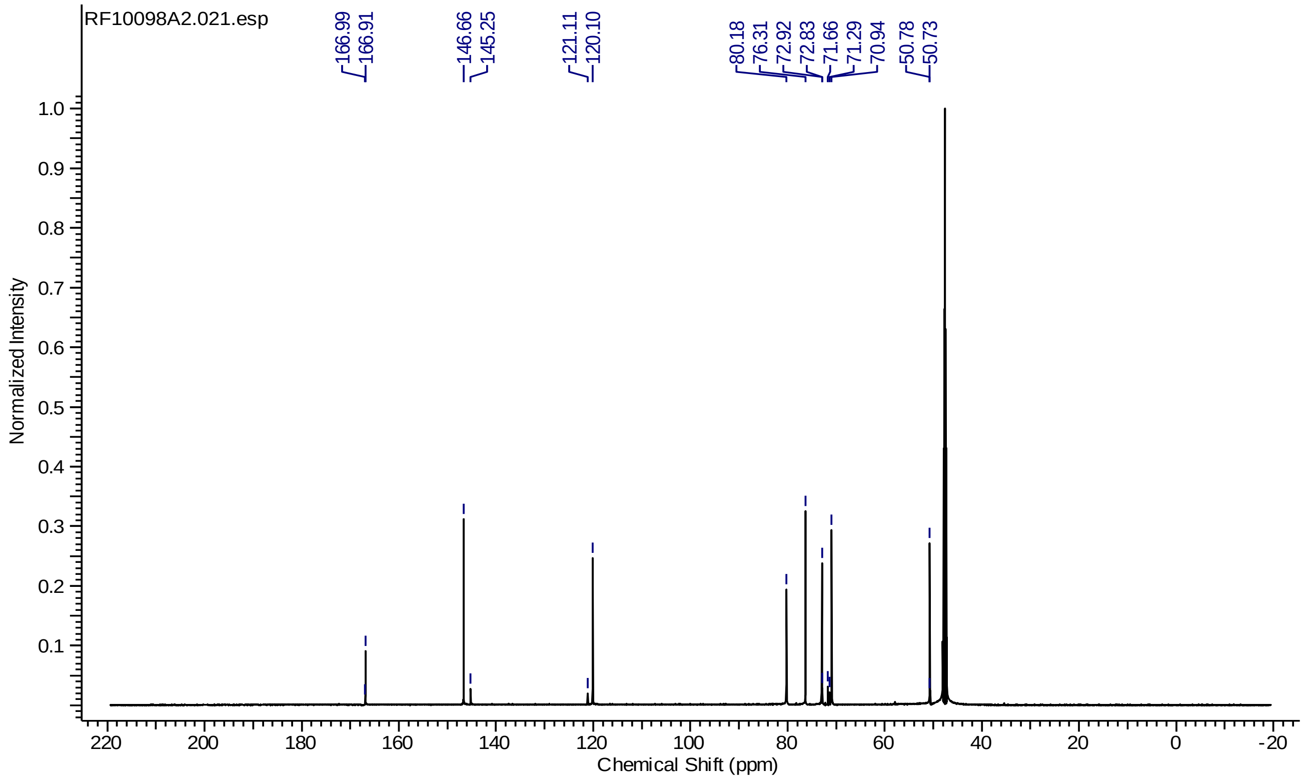

2*S*,3*S*,4*S*)-2-(Dimethoxymethyl)tetrahydrofuran-3,4-diol *anti*-9 and (2*R*,3*S*,4*S*)-2-(Dimethoxymethyl)tetrahydrofuran-3,4-diol *syn*-9

^{1}H NMR (600 MHz, MeOH-d_4)

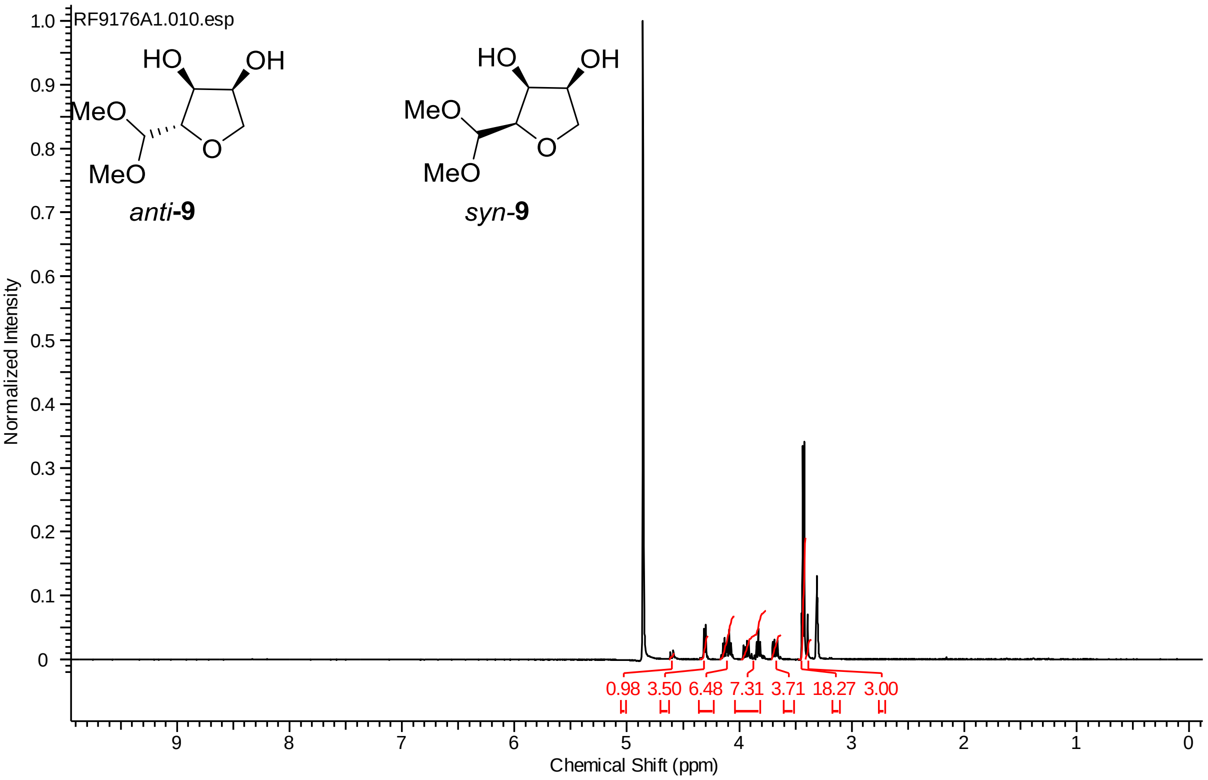

^{13}C NMR (150 MHz, MeOH-d_4)

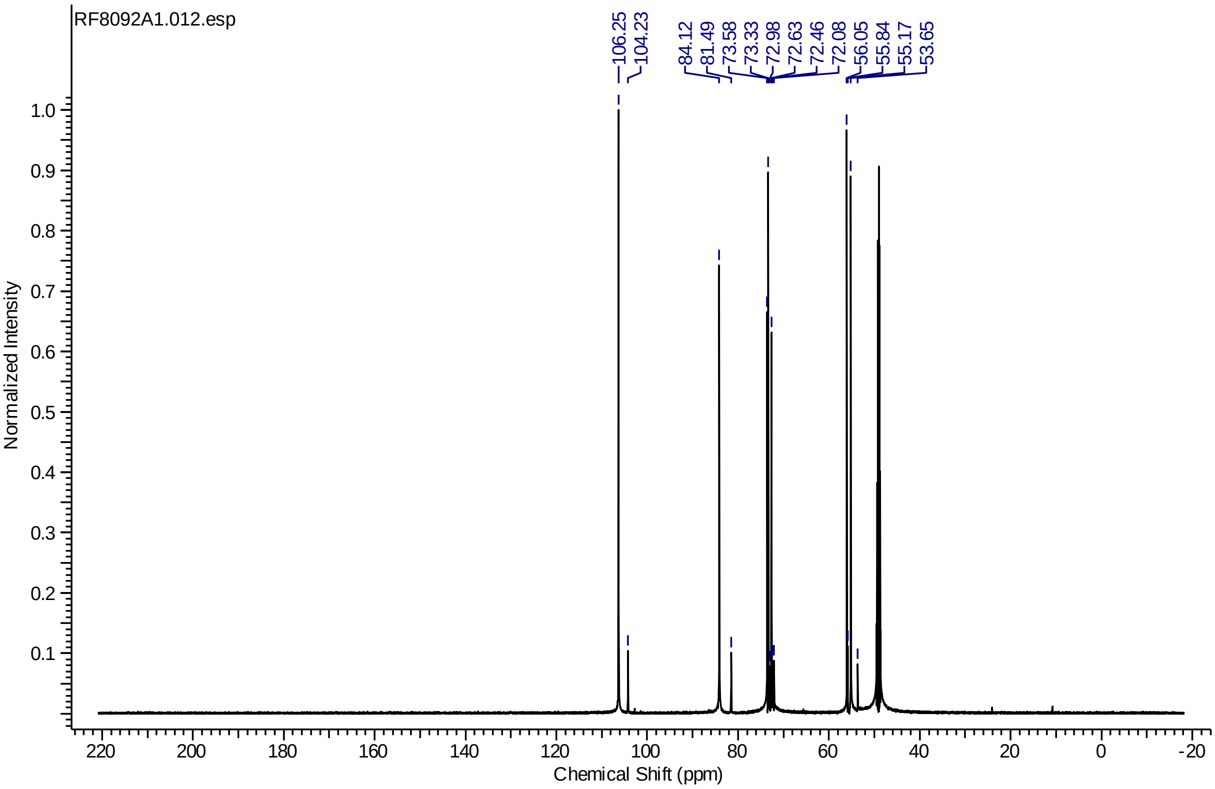

(2*R*,3*S*,4*R*)-2-(Hydroxymethyl)tetrahydrofuran-3,4-diol *anti*-10 and (2*S*,3*S*,4*R*)-2-(Hydroxymethyl)tetrahydrofuran-3,4-diol *syn*-10

^{1}H NMR (600 MHz, D_2O)

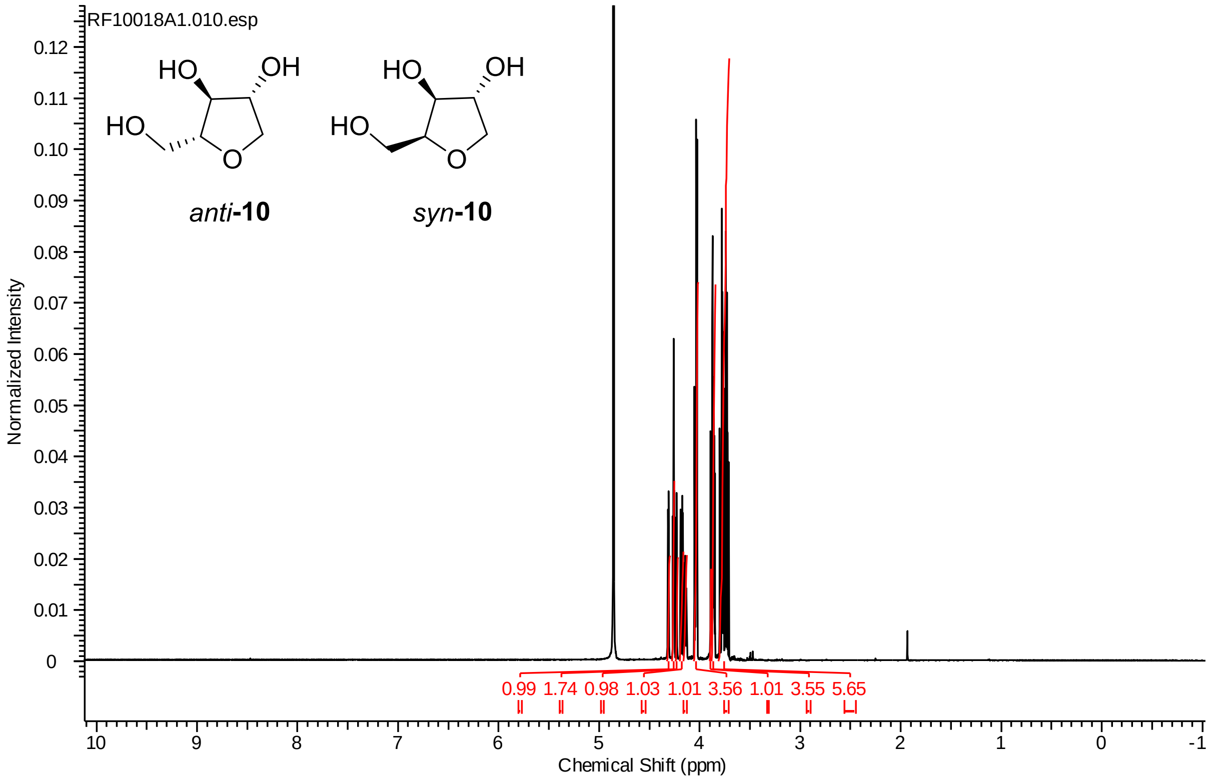

^{13}C NMR (150 MHz, D_2O with MeOH standard)

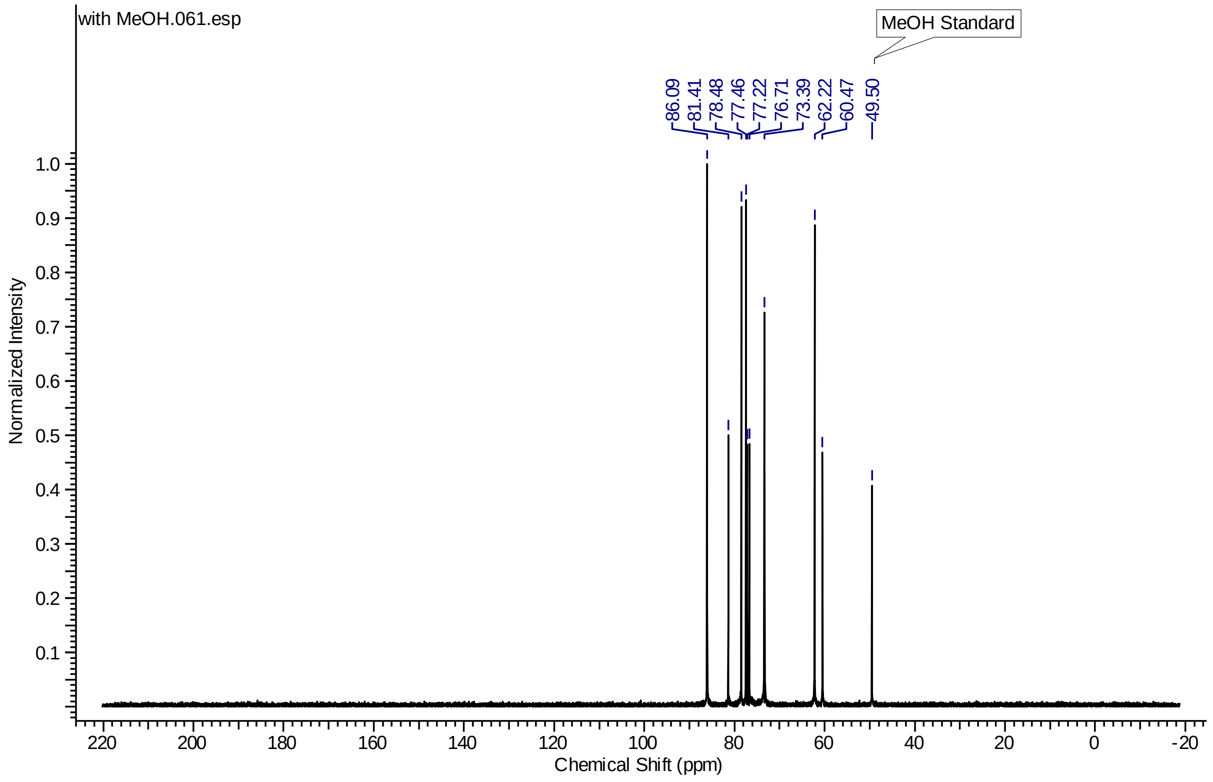

(2*R*,3*R*,4*R*,5*S*)-2-(Hydroxymethyl)-5-methyltetrahydrofuran-3,4-diol *syn*-11 and (2*S*,3*R*,4*R*,5*S*)-2-(Hydroxymethyl)-5-methyltetrahydrofuran-3,4-diol *anti*-11'

^{1}H NMR (600 MHz, D_2O)

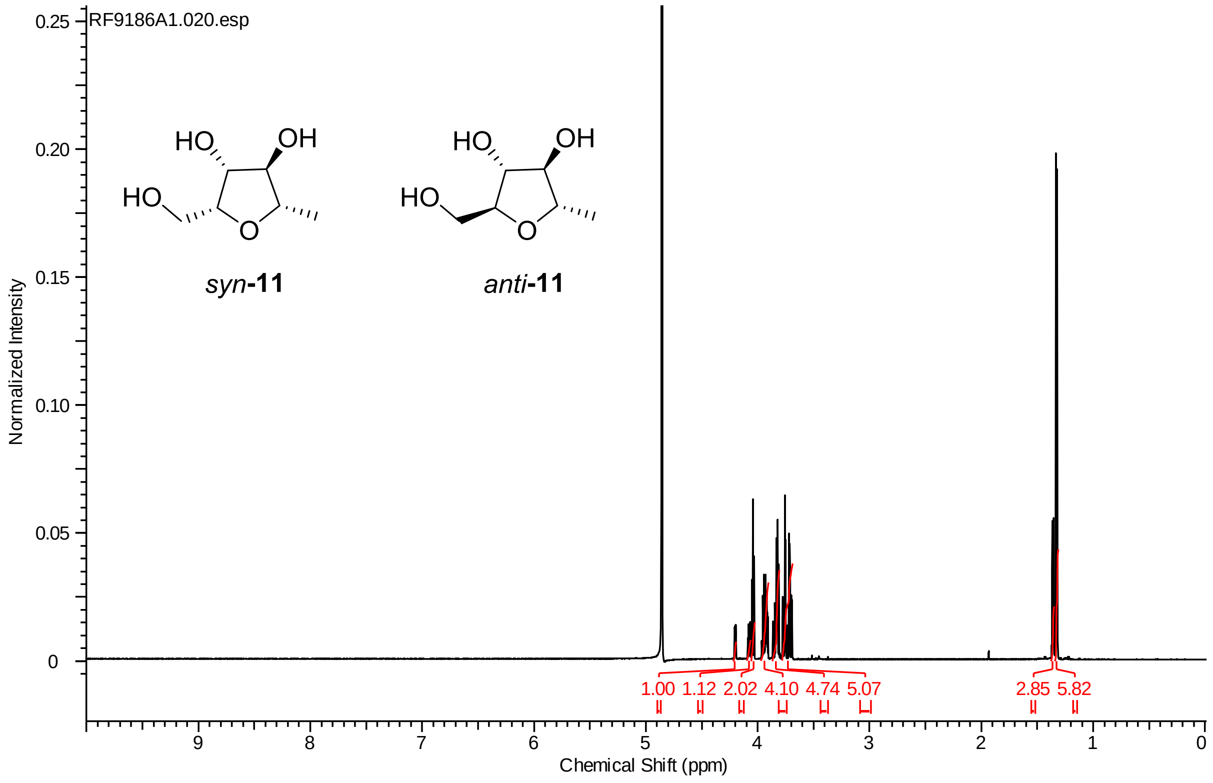

^{13}C NMR (150 MHz, D_2O with MeOH standard)

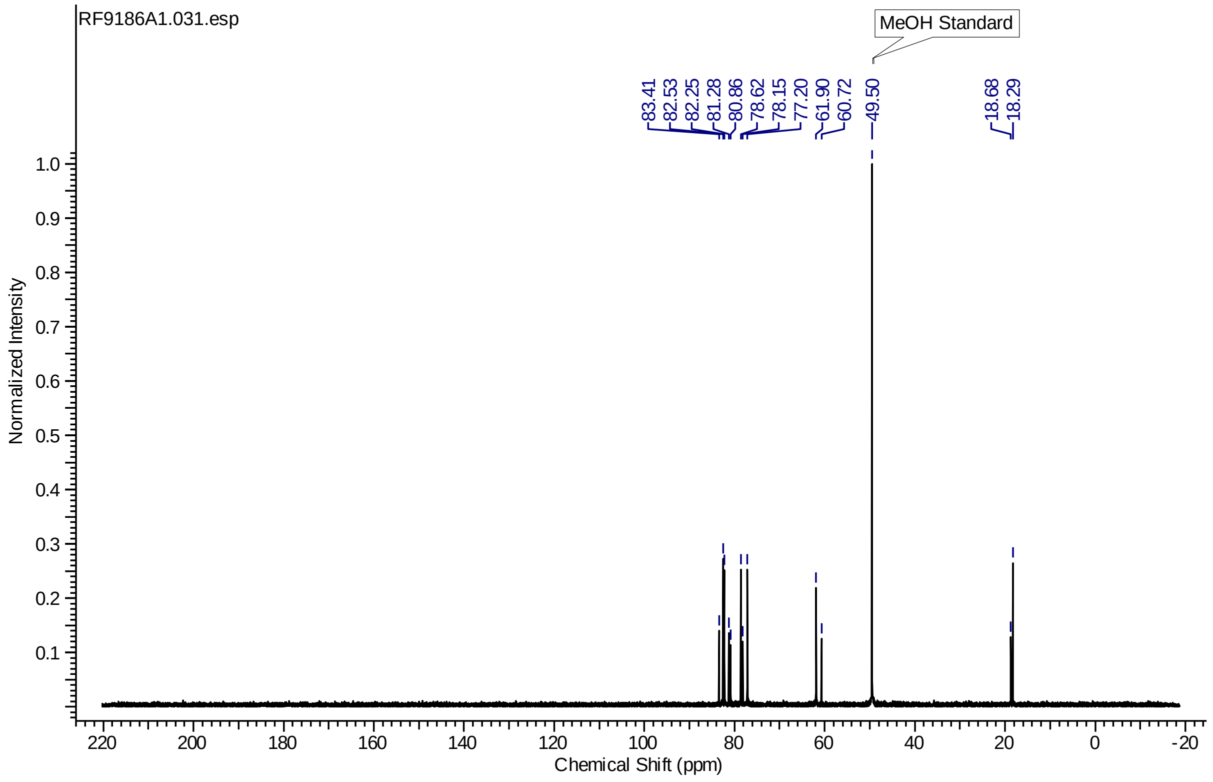

(2*R*,3*S*,4*R*,5*S*,*E*)-6-(2,2-Dimethylhydrazono)hexane-1,2,3,4,5-pentaol 14

^{1}H NMR (600 MHz, DMSO-d$_6$)

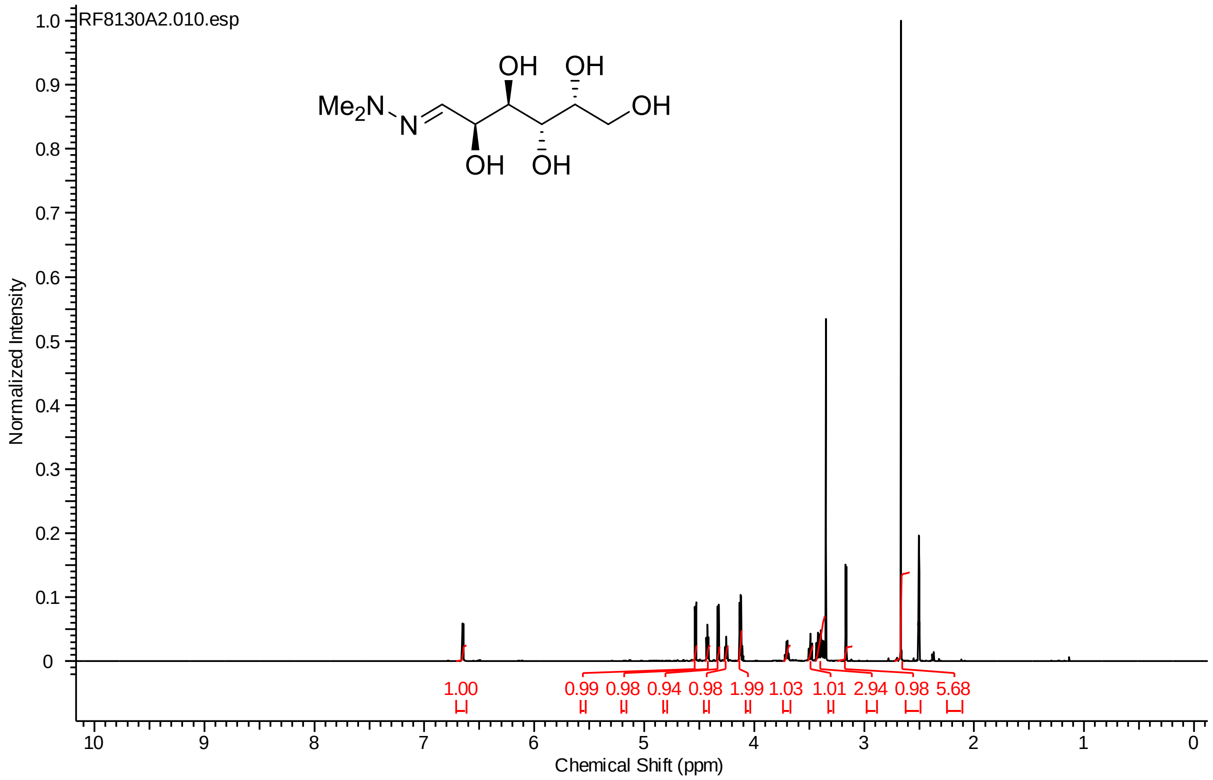

^{13}C NMR (150 MHz, DMSO-d$_6$)

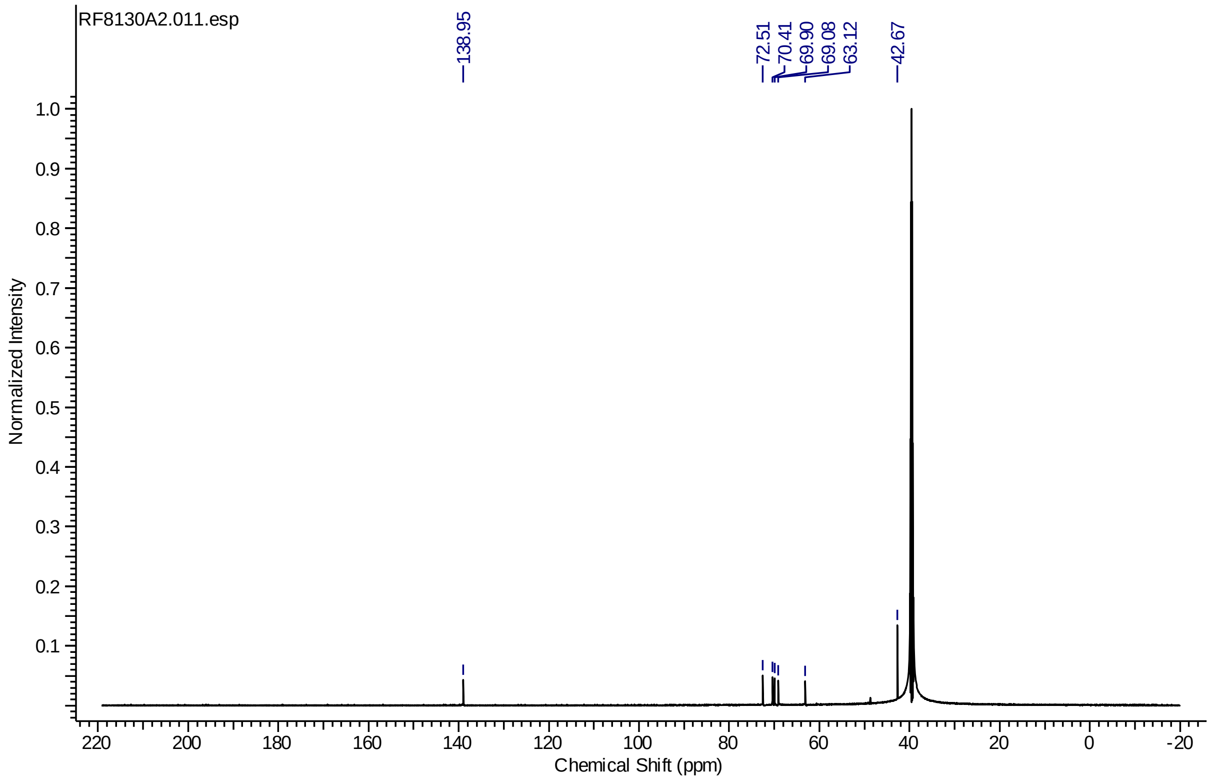

(2*R*,3*S*,4*R*,5*R*)-2-((*E*)-(2,2-Dimethylhydrazono)methyl)-5-(hydroxymethyl)tetrahydrofuran-3,4-diol 15

^{1}H NMR (600 MHz, D_2O)

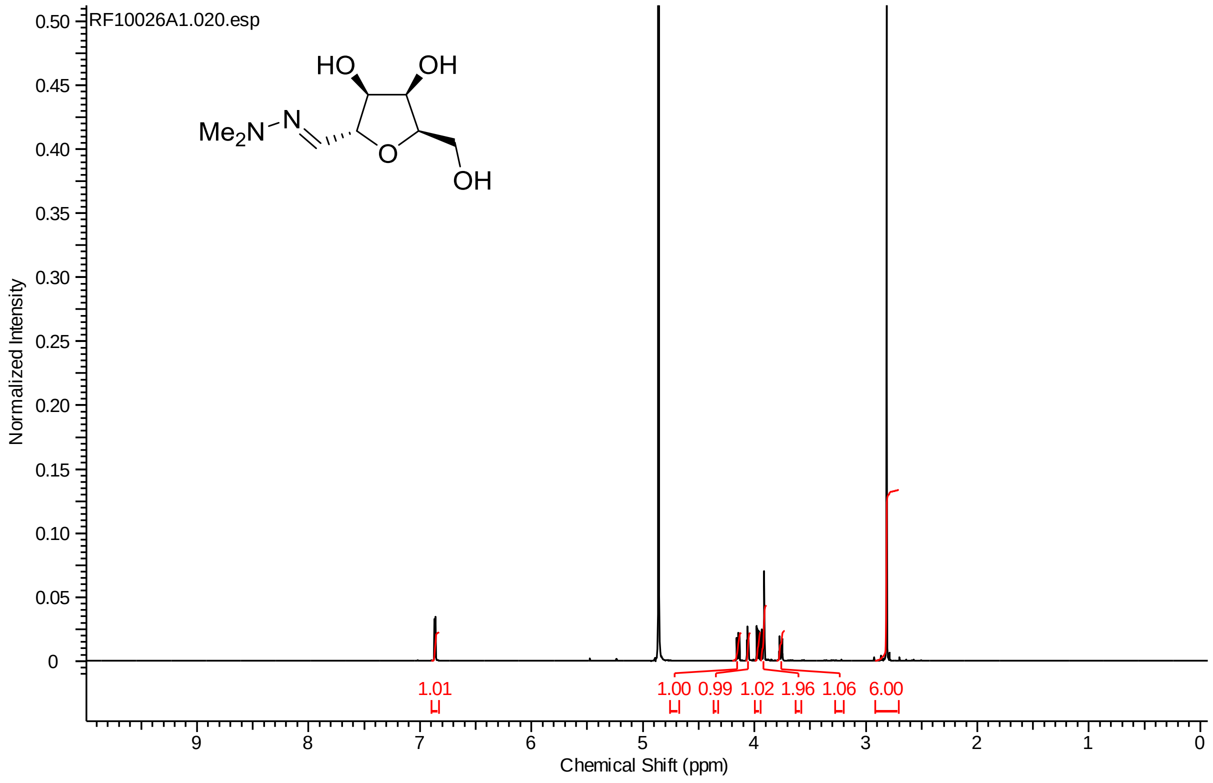

^{13}C NMR (150 MHz, D_2O with MeOH standard)

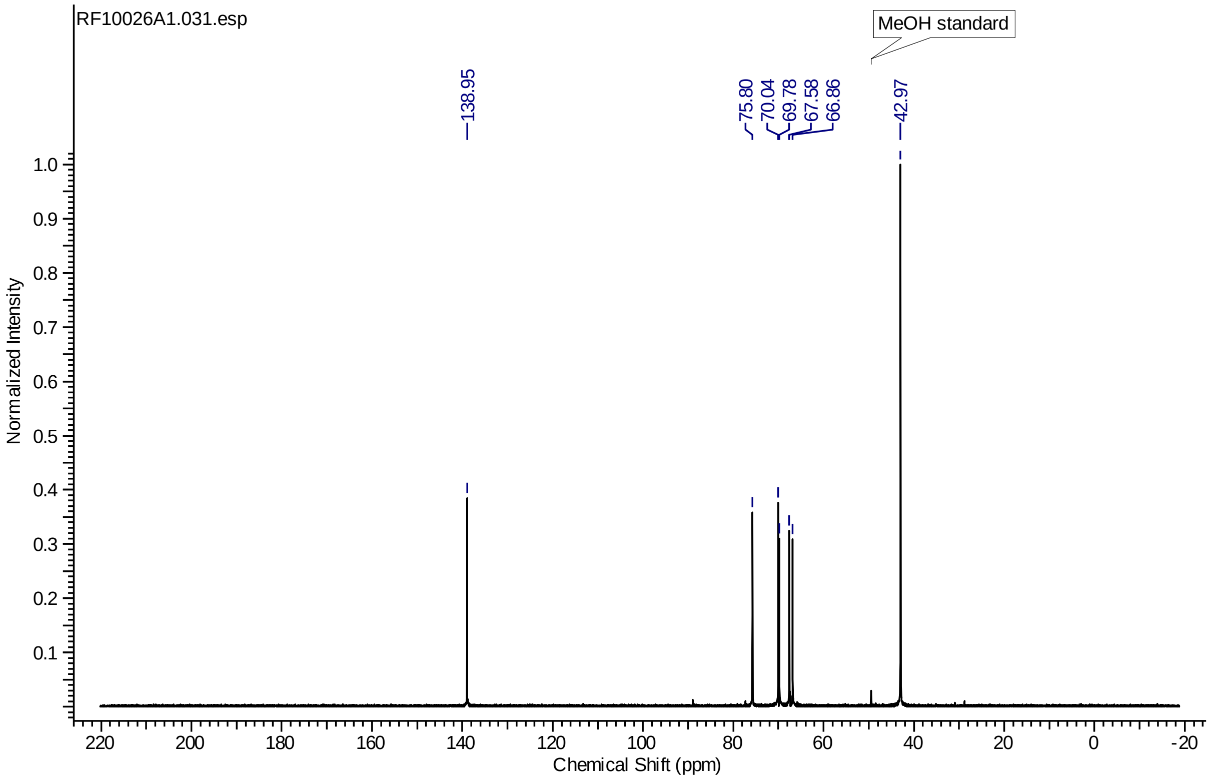

(2*S*,3*S*,4*S*,5*R*)-2-((*E*)-(2,2-Dimethylhydrazono)methyl)tetrahydro-2*H*-pyran-3,4,5-triol 16

^{1}H NMR (600 MHz, MeOH-d$_4$)

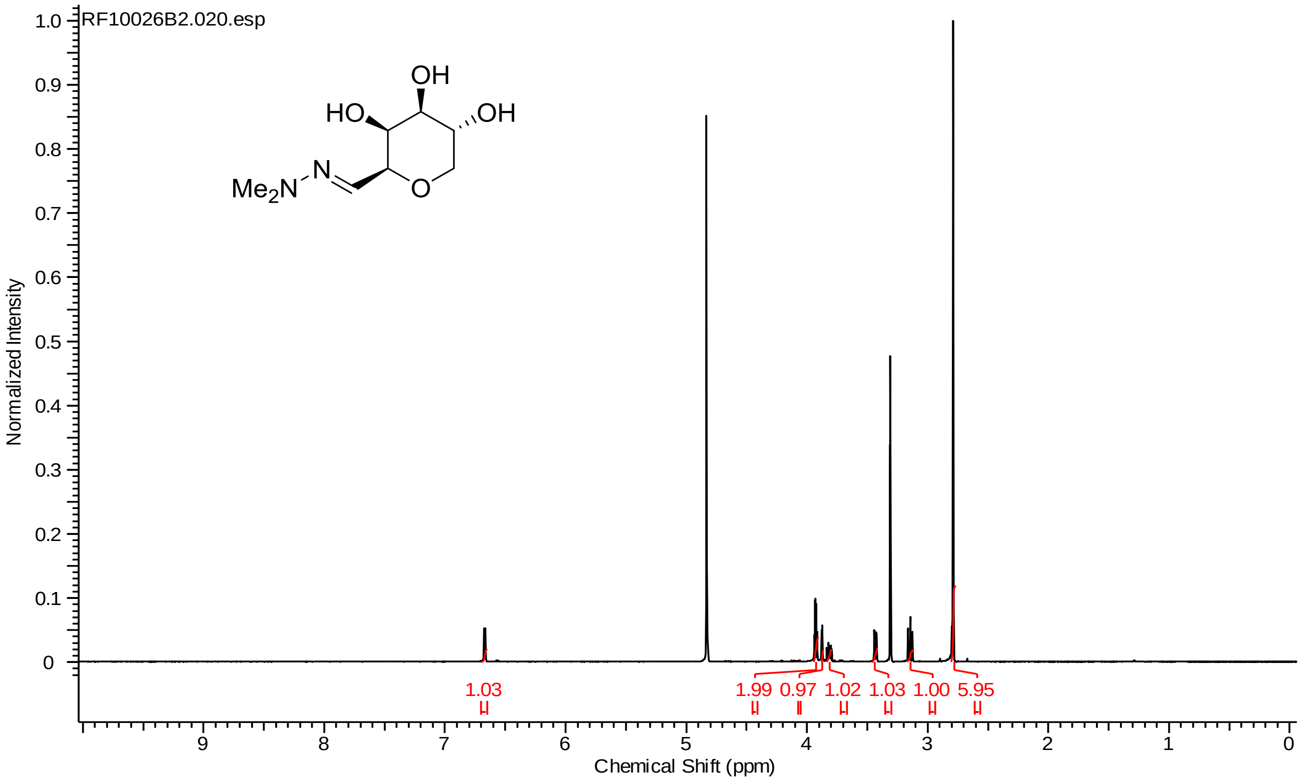

^{13}C NMR (150 MHz, MeOH-d$_4$)

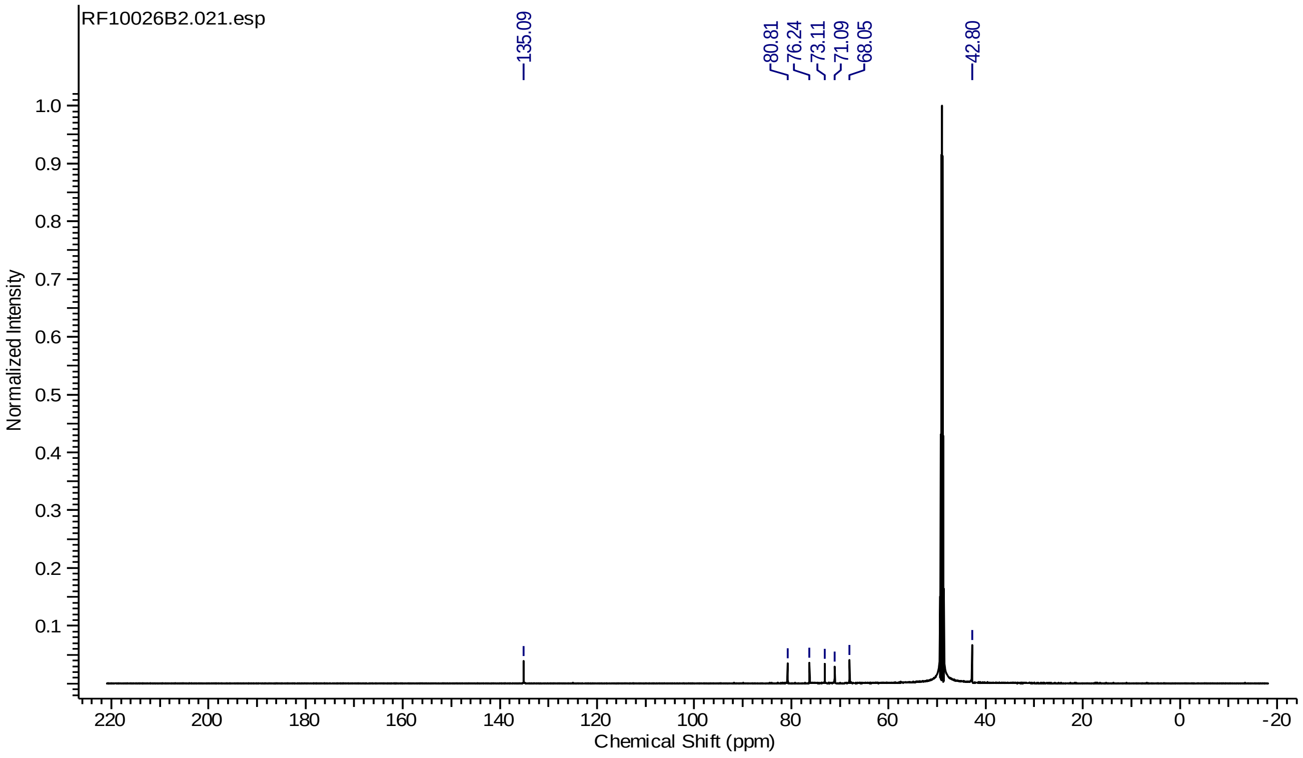

5. Crystallographic analysis of hydrazone *anti*-3a

Single X-ray diffraction data for hydrazone *anti*-**3a** was collected using an *Agilent SuperNova (Dual Source)* single crystal X-ray diffractometer equipped with an *Atlas CCD Detector*. The diffraction experiment was conducted at 150 K using CuK_α radiation (λ = 1.54184 Å). Data collection and processing was accomplished using the *CrysAlisPro* program.[5] Empirical absorption correction was performed using spherical harmonics implemented in the *SCALE3 ABSPACK* scaling algorithm.[5] Structure solution and refinement were accomplished using *SHELXS-97* and *SHELXL-97*, respectively.[6] The structure was solved using direct methods. All non-hydrogen atoms were refined anisotropically, while hydrogen atoms associated with carbon and oxygen atoms were refined isotropically in geometrically constrained positions. Crystallographic and refinement parameters are shown in Table S1.

Table S1. Crystallographic and refinement parameters for hydrazone *anti*-**3a**.

	hydrazone *anti*-**3a**
Empirical formula	$C_7H_{14}N_2O_3$
Formula weight / g mol^{-1}	174.2
Temperature / K	150.00(10)
Crystal system	orthorhombic
Space group	$P2_12_12_1$
a / Å	5.08850(10)
b / Å	17.8742(3)
c / Å	19.7947(3)
α / °	90
β / °	90
γ / °	90
Volume / Å^3	1800.38(5)
Z	8
ρ_{calc}/ g cm^{-3}	1.285
μ / mm^{-1}	0.843
F(000)	752
Crystal size / mm^3	0.31 × 0.04 × 0.03
Radiation	CuK_α (λ = 1.5418 Å)
Index ranges	$-3 \leq h \leq 6$, $-21 \leq k \leq 16$, $-23 \leq l \leq 17$
Reflections collected	4258
Unique reflections	2812
R_{int}	0.0197
Reflections with $I \geq 2\sigma(I)$	2639
Number of parameters	233
Final R indexes [$I \geq 2\sigma(I)$]	R_1 = 0.0281, wR_2 = 0.0659
Final R indexes [all data]	R_1 = 0.0309, wR_2 = 0.0677
Largest diff. peak/hole / e Å^{-3}	0.160 / -0.138
CCDC deposition number	1411520

Full List of Authors for reference 10a from the manuscript:

J. Eron, Jr., P. Yeni, J. Gathe, Jr., V. Estrada, E. DeJesus, S. Staszewski, P. Lackey, C. Katlama, B. Young, L. Yau, D. Sutherland-Phillips, P. Wannamaker, C. Vavro, L. Patel, J. Yeo, M. Shaefer, *The Lancet*, *368*, 476-482

---

[1] A. P. Tantsyrev, L. V. Kanitskaya, A. V. Rokhin, G. G. Levkovskaya, *Rus. J. Org. Chem.* **2004**, *40*, 1735–1736.

[2] J. F. Alfaro, T. Zhang, D. P. Wynn, E. L. Karschner, Z. S. Zhou, J. F. Alfaro, T. Zhang, D. P. Wynn, E. L. Karschner, Z. S. Zhou *Org. Lett.* **2004**, *6*, 3043–3046.

[3] S. J. Mantel1, P. S. Ford, D. J. Watkin, G. W. J. Fleet, D. Brown, *Tetrahedron* **1993**, *49*, 3343–3358.

[5] *CrysAlisPro*, Agilent Technologies, Version 1.171.37.34 (release 22-05-2014 CrysAlis171 .NET)

[6] G. M. Sheldrick, *Acta Crystallogr., Sect. A: Found. Crystallogr.* **2008**, *64*, 112–122.
